# Urbanization Impact on Regional Climate and Extreme Weather: Current Understanding, Uncertainties, and Future Research Directions

**DOI:** 10.1007/s00376-021-1371-9

**Published:** 2022-01-25

**Authors:** Yun Qian, T. C. Chakraborty, Jianfeng Li, Dan Li, Cenlin He, Chandan Sarangi, Fei Chen, Xuchao Yang, L. Ruby Leung

**Affiliations:** 1 Pacific Northwest National Laboratory, Richland, WA 99354 USA; 2 Yale University, New Haven, CT 06520 USA; 3 Department of Earth and Environment, Boston University, Boston, MA 02215 USA; 4 National Center for Atmospheric Research, Boulder, CO 80301 USA; 5 Indian Institute of Technology, Madras, Chennai, Tamil Nadu 600036 India; 6 Zhejiang University, Hangzhou, 310027 China

**Keywords:** urbanization, regional climate, extreme weather, urban heat island, urban flooding, 城市化, 区域气候, 极端天气, 城市热岛效应, 城市洪水

## Abstract

Urban environments lie at the confluence of social, cultural, and economic activities and have unique biophysical characteristics due to continued infrastructure development that generally replaces natural landscapes with built-up structures. The vast majority of studies on urban perturbation of local weather and climate have been centered on the urban heat island (UHI) effect, referring to the higher temperature in cities compared to their natural surroundings. Besides the UHI effect and heat waves, urbanization also impacts atmospheric moisture, wind, boundary layer structure, cloud formation, dispersion of air pollutants, precipitation, and storms. In this review article, we first introduce the datasets and methods used in studying urban areas and their impacts through both observation and modeling and then summarize the scientific insights on the impact of urbanization on various aspects of regional climate and extreme weather based on more than 500 studies. We also highlight the major research gaps and challenges in our understanding of the impacts of urbanization and provide our perspective and recommendations for future research priorities and directions.

## 摘 要

城市环境位于社会、文化和经济活动的结合点,城市下垫面具有独特的生物物理特征。在城市化过程中持续的基础设施建设导致自然景观被建筑物替代。在过去很长时间,绝大部分城市影响天气气候的研究和城市热岛效应(城市及上空温度高于周边地区)有关。除了城市热岛效应,城市化也影响大气湿度、风、边界层结构、云的形成、污染物扩散、降水和暴雨。在这篇综述文章中,我们阅览了超过五百篇文献,从观测和模拟两个方面,首先介绍了用于城市化及影响研究的数据资料和方法,总结了城市化影响区域气候和极端天气的各个领域的科学要点。我们也例举了在理解城市化影响方面目前存在的主要问题和挑战,提出了相应的未来研究重点和方向。

**Article Highlights:**

- As urban areas expand and populations grow, we urgently need to better understand cities and their interactions with weather and climate.
- Urbanization can impact heat waves, atmospheric moisture, clouds, wind patterns, air pollution, boundary-layer, precipitation, and storms.
- Research gaps due to complexity of urban areas and deficiencies in current methods are identified and future priorities are highlighted.

## 1. Introduction

Cities are the nexus of modern social, cultural, and economic activities, and they have unique biophysical, morphological, and thermodynamic properties due to the physical process of urbanization, where natural landscapes are replaced by buildings, roads, parking lots, etc. (built-up structures). Urban areas are also centers of resource utilization, with high energy and water consumption (Chen and Chen, 2016), which lead to anthropogenic heat fluxes (Zhang et al., 2013) and greenhouse gas and aerosols emissions (Fig. 1). These characteristics and activities give rise to unique urban climates from micro to regional scales (Oke, 1981; Arnfield, 2003; Kalnay and Cai, 2003; Rizwan et al., 2008; Feng et al., 2012; Yan et al., 2016; Chapman et al., 2017a; Oke et

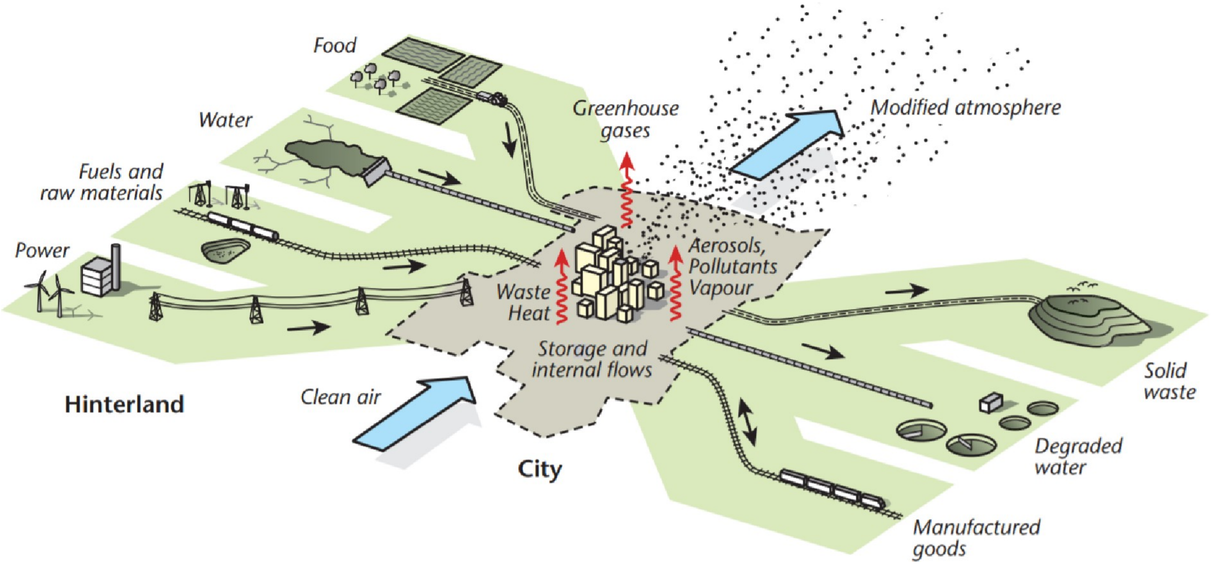

**Fig. 1.** Representation of inputs to and outputs from an urban ecosystem [Reprinted from Fig. 1.4 in *Urban Climates* by Oke et al. (2017), © 2017 Cambridge University Press. Reproduced with permission of The Licensor through PLSclear.].

al., 2017; Zhou et al., 2017; Chakraborty and Lee, 2019; Sharma et al., 2020). Currently, over 3.5 billion people, or half of the global population, live in urban areas. This percentage is projected to grow to around 68% by the middle of this century (over 89% for the United States) (UNDESA, 2018). More people in cities increase the demand for services, housing, and utilities, and changes of land cover and infrastructure features modulate local and regional weather and climate through both biophysical and biogeochemical processes (Niyogi et al., 2011; Zhong et al., 2015, 2017, 2018; Sarangi et al., 2018; Yang et al., 2019a).

The vast majority of studies on urban perturbation of local weather and climate have been centered on the urban heat island (UHI) effect, i.e., the relatively higher temperature in cities compared to their surroundings (Fig. 2). This is one of the oldest and most researched topics in urban climatology, dating back to the work by Howard (1833), which found that the air temperature in London's urban center was distinctly higher than its surroundings. Since this pioneering work, studies characterizing UHI intensity have been conducted using various methodologies for hundreds of cities (Bornstein, 1968; Oke, 1982a; Douglas, 1983; Wang et al., 1990; Changnon, 1992; Saitoh et al., 1996; Arnfield, 2003; Lin and Yu, 2005; Ren et al., 2007; Roth, 2007; Hamdi, 2010; Elagib, 2011; Camilloni and Barrucand, 2012; Ngie et al., 2014; Founda et al., 2015; Santamouris, 2015; Torres-Valcárcel et al., 2015; Sachindra et al., 2016; Deilami et al., 2018; Wu and Ren, 2019). Besides the UHI effect, urbanization generates or modulates other meteorological phenomena (Miao and Chen, 2008; Shao et al., 2013; Han et al., 2014b; Martilli, 2014; Song et al., 2014; Ma et al., 2016; Liang et al., 2018; Huang et al., 2019; Huszar et al., 2020; Hajmohammadi and Heydecker, 2021). These include the urban moisture island (UMI) or urban dry island (UDI) effect, a measure of the difference in moisture content between (for UDI, the generally lower moisture content in) urban air and the air of the rural background (Kratzer et al., 1962; Du et al., 2019), effects on boundary layer structure and winds, cloud formation and storm characteristics and propagation, dispersion of air pollutants (Giovannini et al., 2014; Zhong et al., 2018), and the potential urbanization-induced modulation of precipitation distribution, intensity, and frequency (Lowry, 1998; Liu and Niyogi, 2019).

Studying, understanding, and predicting urban meteorology and hydrology is particularly pressing in the context of extreme weather and climate change. Urban environments are highly vulnerable to extreme hydrometeorological events (including flash floods; Chang and Franczyk, 2008; Zhang et al., 2014c, 2018), which can result in widespread disruption to critical physical, social, and institutional systems (Cho and Chang, 2017) and whose patterns are expected to be altered by rising greenhouse gas concentrations. Flooding in urban environments can occur anywhere due to the prevalence of impervious surfaces (Jha et al., 2012), and flooding risks are not limited to floodplain or coastal bodies of water. The recent devastating floods during July 2021 in Zhengzhou city in central China, home to 12 million people, resulted in more than 300 deaths and incalculable dollars in property loss. Similarly, the New York City metropolitan area was struck by the remnants of Hurricane Ida in early September 2021, which flooded subways, roads, and homes and killed at least 52 people across the Northeast. However, the physical mechanisms, including the large-scale circulations and local-scale urban–atmosphere interactions, that are responsible for these extreme events remain uncertain and poorly understood.

In this review article focusing on interactions between urban areas and the atmosphere, weather, and climate, we first introduce the datasets and methodologies used for detecting urban impacts based on observations (section 2) and mod-

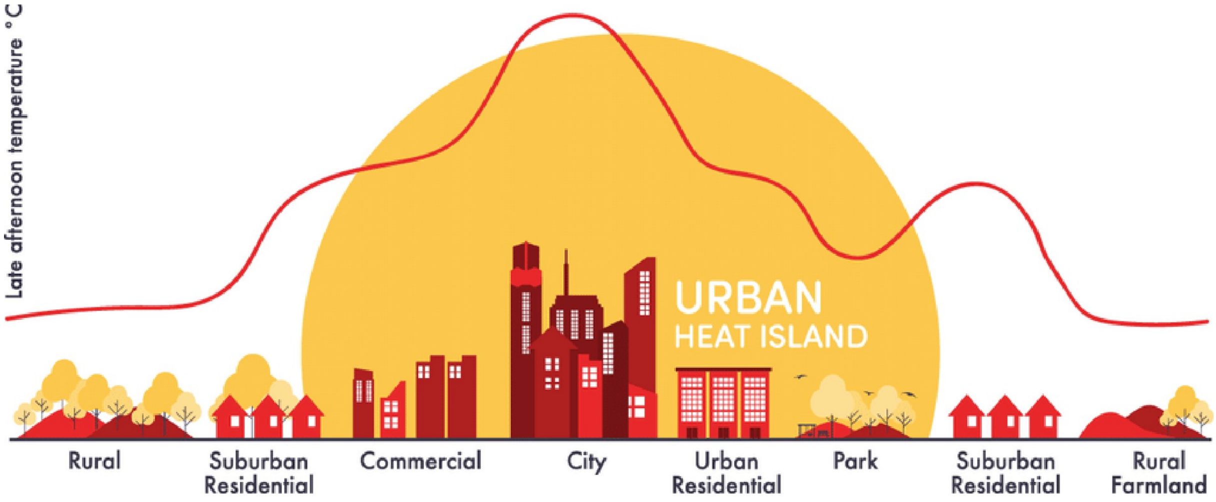

**Fig. 2.** Schematic picture of the urban heat island effect [https://community.wmo.int/activity-areas/urban/urban-heat-island. Courtesy of Kamyar Fuladlu. © 2020 World Meteorological Organization (WMO)].

eling tools (section 3). Then we summarize the research progress on the impact of urbanization on temperature and heat waves, based on observational evidence (section 4) and model simulations (section 5), on wind, boundary layer structure, clouds and air pollution (section 6), and on precipitation and extreme events (section 7), based on more than 500 studies. Finally, we highlight the current understanding and major research gaps and challenges and provide our perspective and recommendations for future research priorities and directions (section 8).

## 2. Methodologies for isolating the urban impact based on observations

### 2.1. *Datasets*

Urban impact on local climate is frequently isolated by comparative analysis of observations in an urban region and its peripheral rural regions. Separation of an urban region from rural areas can be done by analyzing spatial urban/rural metadata (Peterson and Vose, 1997; Peterson et al., 1999), satellite-measured nighttime lights data (Owen, 1998; Gallo et al., 1999; Hansen et al., 2001), population datasets at various scales (Ren et al., 2008; Fujibe, 2009), land cover data (Wang and Ge, 2012; Chrysanthou et al., 2014; Wang et al., 2015a; Demuzere et al., 2020; Jiang et al., 2020), etc. The UHI effect is quantified using in situ observations of ambient air temperature or remotely sensed estimates of radiometric skin temperature (Voogt, 2007; Stewart, 2011b; Chakraborty and Lee, 2019). However, several variations exist within each measurement typology. In situ measurements can be from stationary weather stations, including standard, research-grade, and crowdsourced observations (Muller et al., 2013; Chapman et al., 2017b; Venter et al., 2021), or via mobile sensors, either mounted on vehicles or incorporated into smart devices (Unger et al., 2001; Rodríguez et al., 2020; Nazarian et al., 2021). Remote measurements can be retrieved through drones, airplanes, or polar orbiting and geostationary satellites (Ngie et al., 2014; Naughton and McDonald, 2019; Zhou et al., 2019).

Similar to the UHI effect, the UDI effect can be measured using in situ observations, either stationary or mobile (Hass et al., 2016; Lokoshchenko, 2017). Unfortunately, remote estimates of humidity or moisture content in urban areas are columnar estimates, which do not provide ground-level estimates of humidity or its proxies without significant ancillary information (Hu et al., 2015). The influence of urbanization on precipitation is usually studied using in situ rain gauges (Golroudbary et al., 2017; Liang and Ding, 2017; Sarangi et al., 2018). Although the current spatial and temporal resolutions of satellites to monitor precipitation [0.1 degree spatial resolution for the Global Precipitation Measurement (GPM) satellite] are often not fine enough to completely isolate small to medium urban areas, they help in differentiating the precipitation distribution over the areas upwind and downwind of cities (Shepherd et al., 2002). Satellite observations of urban cloud cover have also been used as a proxy for the propensity of urban precipitation (Theeuwes et al., 2019). Satellite observations are also increasingly being used to monitor urban air quality in terms of overall aerosol loading and separate urban air pollutants (Engel-Cox et al., 2004, Chakraborty et al., 2021a; Vohra et al., 2021).

### 2.2. *Canopy urban heat islands (CUHI)*

Traditionally, the UHI was defined as the difference in near-surface air temperature between the urban core and its rural hinterland. This is now commonly known as the canopy UHI, or CUHI, since air temperature measurements are usually taken at 1.5 m to 2 m above the surface, well within the urban canopy (Oke, 1976, 2006). In situ measurements from weather stations can be used to measure the CUHI. For this, previously established stations set up for standard weather monitoring are generally insufficient, since they are usually at nearby airports, which do not represent the complete urban environment (WMO, 2008; Konstantinov et al.,

2018). Even when research-grade sensors are set up within and outside cities to measure the CUHI using urban–rural site pairs, they focus on the CUHI temporal variability. This is because it is difficult to set up sufficient observational points to fully capture the spatial variability of urban microclimate (Hass et al., 2016; Liu and Niyogi, 2019; Chakraborty et al., 2020).

To determine the spatial characteristics of the CUHI, one may rely on networks of weather stations or measurements taken during mobile transects. The first method requires significant logistical support and funding, with many past urban meteorological networks no longer operational (Muller et al., 2013). Recently, crowdsourced measurements from private weather stations have been used to monitor the CUHI (Chapman et al., 2017b; Venter et al., 2021). These have the advantage of wide spatial coverage within cities with station maintenance provided by the private owners instead of by researchers (Meier et al., 2017; Venter et al., 2021). The second method can take many forms, with the traditional approach involving driving vehicles with meteorological sensors within cities (Chakraborty et al., 2017; Yokoyama et al., 2018). Several individual and extended studies have now started collecting urban micro-climate data using sensors mounted on bicycles (Ziter et al., 2019; Cao et al., 2020; Rodríguez et al., 2020). A few studies have incorporated ambient measurements within smart devices (for instance, phones and wearables) to take advantage of the exponential rise in their use in recent years (Ueberham and Schlink, 2018; Nazarian et al., 2021). Similar methods can also be used to measure the UDI and UMI. For instance, Wang et al. (2021b) recently calculated the UMI using absolute humidity measurements from urban and rural stations over Hong Kong.

Another method to estimate urbanization-induced effects is to compare the climatic trend at urban stations with that at nearby rural stations. As it is difficult to find records specifically from rural sites in China, Jones et al. (2008) used sea surface temperature adjacent to east China as a reference and concluded that the urbanization-induced warming over China was about 0.1°C (10 yr)$^{-1}$ during 1951–2004. Kalnay and Cai (2003) proposed the "observation-minus-reanalysis" (OMR) method to estimate the impact of urbanization and other land cover changes on temperature changes over the US. The rationale for this method is that reanalysis datasets are insensitive to land surface properties and the changes in these properties, since surface (especially urban surface) observations are rarely assimilated. Therefore, comparing the temperature trends between observations and reanalysis can isolate the impact of urbanization and other land use changes. However, Wang et al. (2013a) found that the systematic bias in the multidecadal variability of the NCEP–NCAR reanalysis data, used by Kalnay and Cai (2003), could modulate the magnitude of OMR trends considerably during different periods.

### 2.3. *Surface urban heat islands (SUHI)*

The advent of satellite products has allowed researchers to remotely measure the radiometric land surface temperature (LST), or skin temperature, over urban areas (Gallo et al., 1995). This has led to a new category of the UHI effect called the surface UHI (SUHI), which is the difference in LST between the urban area and its surrounding rural area. Due to the worldwide coverage of polar orbiting satellites, both intra- and inter-urban variability in SUHI is much easier to estimate compared to the same for the CUHI. Moreover, since an individual satellite uses the same sensor to "view" all terrestrial surfaces, there are less issues with differences in sensor accuracy and drift when examining the SUHI. As such, almost all multi-city comparisons of UHI have used satellite-derived products (Imhoff et al., 2010; Peng et al., 2012; Clinton and Gong, 2013; Li et al., 2017; Chakraborty and Lee, 2019). Note that unlike in situ measurements of CUHI using stationary weather stations, only geostationary satellites or continuous measurements of outgoing longwave radiation can provide diurnal examination of the SUHI (Inamdar et al., 2008; Weng and Fu, 2014; Chakraborty et al., 2017), with polar orbiting satellites providing SUHI estimates only during specific overpass times (Chakraborty and Lee, 2019). Although less common, the SUHI and urban LST can also be examined using aircraft and drones (Lo et al., 1997; Naughton and McDonald, 2019). Similarly, ground-based observations of infrared radiation can also be used to estimate LST and SUHI (Johnson et al., 1991; Stoll and Brazel, 1992; Voogt and Oke, 1998; Chakraborty et al., 2017). Figure 3 provides an overview of the advantages and disadvantages of urban temperature measurements from weather stations and satellites.

### 2.4. *Other urban islands/ pollution dome*

Beyond CUHI and SUHI, UHIs have also been defined for the boundary layer, or the boundary layer UHI, and for the sub-surface. The positive SUHI affects both the boundary layer, with higher and warmer boundary layers over urban areas (Oke, 1976, 1995), and urban sub-surfaces (Ferguson and Woodbury, 2007; Menberg et al., 2013). Because of the dearth of simultaneous observations of the boundary layer temperature, which requires either radiosondes or ground-based microwave radiometers, most studies on the boundary layer UHI are based on numerical model simulations (Barlow, 2014; see section 4). Sub-surface UHIs have been detected using data from observational wells or from empirical models based on satellites (Menberg et al., 2013; Zhan et al., 2014).

### 2.5. *Moisture and precipitation*

Few studies have simultaneously measured air temperature and humidity within cities and provided their bulk impact on thermal heat stress (for instance, Harlan et al., 2006; Pantavou et al., 2018). For precipitation, observational studies are even more rare than studies on CUHI. The few studies on this quantity can be divided into those using rain gauges, either using networks within cities or urban–rural pairs, or indirect measurements from ground and satellite-mounted radars (Shepherd et al., 2002; Kisht-

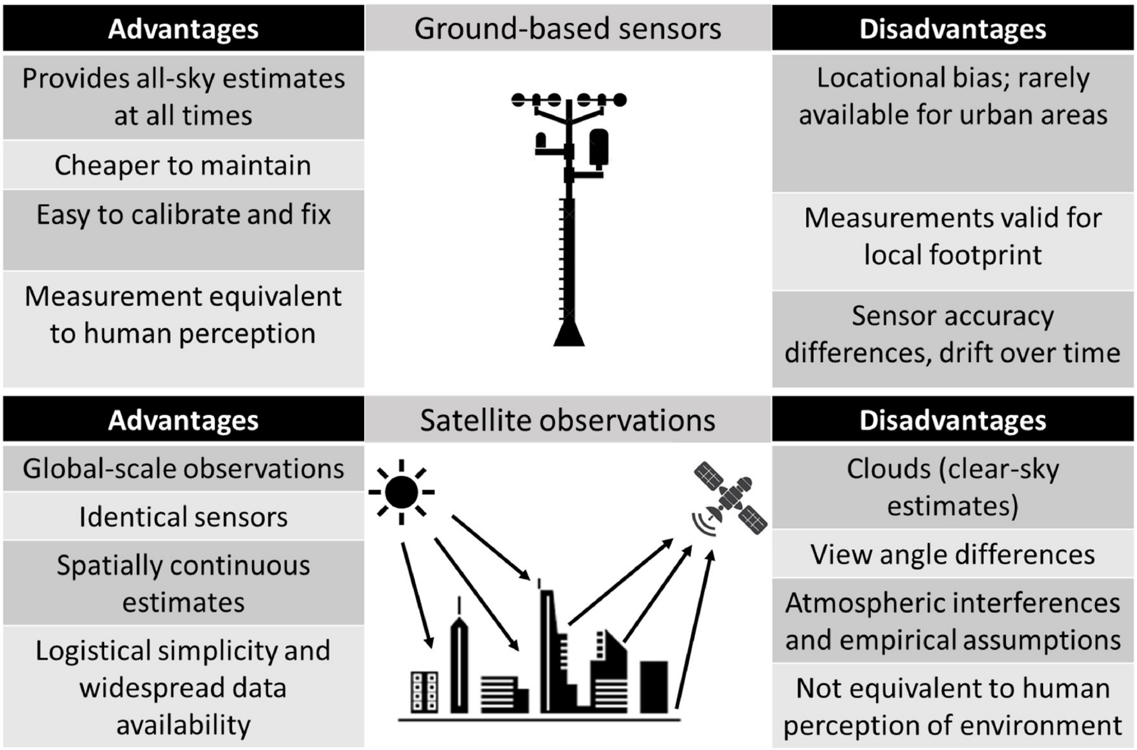

**Fig. 3.** Overview diagram of ground-based versus satellite observations of urban temperatures, their advantages, and their disadvantages.

awal et al., 2010; Yang et al., 2013; Golroudbary et al., 2017; Liang and Ding, 2017; Sarangi et al., 2018). Recently, based on radar and satellite data, Li et al. (2021) developed an hourly precipitation dataset with 4-km spatial resolution over the US for 2004–17. This dataset can separate different precipitation types, from mesoscale convective systems to isolated deep convection to non-convective precipitation, which provides an opportunity to quantify the urban effect on different types of precipitation and weather events.

## 3. An overview of modeling tools for urban study

### 3.1. *Numerical urban modeling embedded in bulk and mesoscale models*

In the past decades, several urban climate/weather models with either empirical or physics-based parameterizations have been developed to study urban areas (Fig. 4). Numerical modeling of urban energy balance has a long history, with the first attempts nearly half a century ago. However, most land and/or atmospheric models did not formally include urban parameterizations until the last two decades. Several studies have comprehensively reviewed different aspects of urban modeling (Brown, 2000; Masson, 2006; Baklanov et al., 2009; Garuma, 2018; Krayenhoff et al., 2021). Here, we briefly summarize the key features and advances in urban modeling.

Many simplified and empirical urban parameterizations were developed based on observation-derived statistical relationships (Oke and Cleugh, 1987; Grimmond et al., 1991; Grimmond and Oke, 2002; Offerle et al., 2003; Hoffmann et al., 2012; Yin et al., 2018; Oliveira et al., 2021). These models do not solve physical equations and require little input forcing data. They have several benefits, including computational efficiency, but it is unclear to what extent these empirical parameterizations can be generalized and applied across global cities and for different background conditions.

Many studies (Myrup, 1969; Bornstein, 1975; Kimura and Takahashi, 1991; Yoshikado, 1992; Avissar, 1996; Ichinose et al., 1999; Taha, 1999; Kusaka et al., 2000; Best, 2005; Dandou et al., 2005; Liu et al., 2006; Wang et al., 2013c; De Ridder et al., 2015) adopted simplified bulk urban parameterizations, or slab urban models embedded in standard surface-layer schemes, or land surface models to represent the zero-order effects of urban surfaces. These bulk/slab urban models typically include (1) a surface energy balance equation, (2) a heat equation to compute surface and soil temperatures, and (3) a traditional surface-layer scheme using the Monin-Obukhov similarity theory to estimate surface fluxes. The relevant model parameters have been further adapted to fit the urban environment, including (1) a prescribed surface roughness length to represent turbulence generated by urban roughness elements and building drags, (2) a low surface albedo to represent solar radiation trapping in urban canyons, (3) a prescribed heat capacity for urban surfaces (e.g., roads, walls, and roofs), (4) a prescribed soil thermal conductivity for urban roads and buildings, and (5) a reduced vegetation fraction. These models

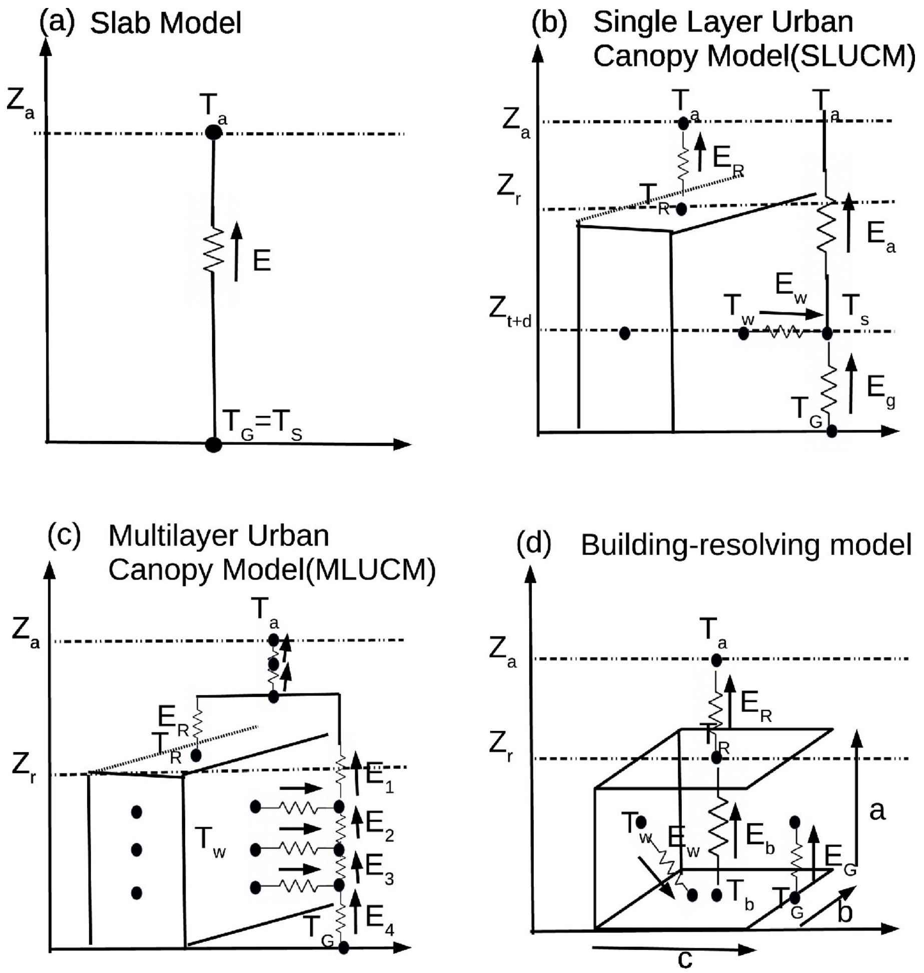

**Fig. 4.** Urban representation models: (a) The slab model, where surface temperature $T_s$ is equal to ground temperature $T_g$, (b) the single layer urban canopy model (SLUCM), (c) the multiple-layer urban canopy model, and (d) the building-resolving model [Reproduced from Garuma (2018), © 2017 Elsevier B.V.].

have a high computational efficiency due to their simplified urban treatments. However, they do not include specific urban features (e.g., building structures) and heavily rely on the surface-layer schemes or land surface models that already exist in mesoscale atmospheric models. It is worth noting that some recent studies (e.g., Wouters et al., 2016) proposed a semi-empirical urban canopy parametrization in order to bridge the gap between the bulk urban models and more sophisticated urban schemes.

The aforementioned empirical urban parameterizations and simplified bulk urban models may arguably be useful for specific applications where key model parameters can be optimized or tuned based on observations. However, they may not fully capture the fundamental urban physical processes, particularly the impact of urbanization on weather and climate (Piringer et al., 2002). This substantially limits these models' applicability and thus their suitability for inclusion in mesoscale or global models.

Some studies (e.g., Mills, 1997; Masson, 2000; Kusaka et al., 2001; Oleson et al., 2008; Li et al., 2016) proposed more sophisticated single-layer urban canopy models (SLUCMs), which generally consist of (1) a street canyon component parameterized to represent the urban geometry (including orientation) and artificial surface, (2) a radiation component parameterized to represent the canyon orientation, diurnal change of solar zenith angle, and shadowing effects from buildings and reflection and trapping of radiation, (3) an urban canopy flow component [e.g., Inoue (1963)'s model], (4) an energy component including energy balance and heat equations for the roof, wall, and road interior temperatures, (5) a surface flux scheme based on the Monin–Obukhov similarity theory, and (6) a thin bucket model for hydrological processes. The SLUCMs estimate the energy and momentum exchange between the urban surface and the atmosphere. Subsequent studies (e.g., Lemonsu and Masson, 2002; Chen et al., 2004; Kusaka and Kimura, 2004; Kusaka, 2009; Oleson et al., 2011; Trusilova et al., 2013) have coupled SLUCMs to mesoscale or global atmospheric models to evaluate urban impacts.

Going beyond SLUCMs, more advanced multi-layer urban canopy models (MLUCMs) have been developed to resolve vertical building effects (e.g., Ca et al., 1999; Martilli, 2002; Dupont et al., 2004; Otte et al., 2004; Kondo et al., 2005; Schubert et al., 2012). One of the most sophisticated MLUCMs is the BEP model (Martilli, 2002), which treats the urban surfaces in a three-dimensional (3D) way to account for the building vertical distribution of sources and sinks of momentum, heat, and moisture fluxes across the entire urban canopy layers. An urban quarter is represented by a horizontal building size, a street canyon width, and a building density as a function of height. Thus, it includes the impact of vertical (walls) and horizontal (streets and roofs) surfaces on momentum (drag force approach), potential temperature, and turbulent kinetic energy (TKE). Other MLUCMs (Dupont et al., 2004; Otte et al., 2004) made some simplifications by only considering 2D buildings (i.e., one horizontal dimension and one vertical dimension) instead of 3D building volume like BEP. Similar to SLUCMs, MLUCMs also account for the street canyon-induced solar radiation reflections and shadowing as well as the trapping of solar and longwave radiation. To leverage the multilayer treatment of MLUCMs, multiple model levels with high vertical resolution within the urban canopy are preferred.

However, most SLUCMs and MLUCMs, including BEP (Martilli, 2002), use a constant building internal temperature by default, which may not be realistic. Thus, further studies (e.g., Ashie et al., 1999; Kikegawa et al., 2003; Salamanca et al., 2010; Yaghoobian and Kleissl, 2012; Lipson et al., 2018; Zarrella et al., 2020) have developed more detailed urban building energy models to improve the simulation of energy exchange between the outdoor/ambient atmosphere and the building interior. The urban building energy models generally include (1) radiation exchanged through windows, (2) heat diffusion via walls, roofs, and floors, (3) heat generated from occupants and equipment, (4) longwave radiation exchanged between indoor surfaces, and (5) indoor heating, ventilation, and air conditioning. For example, Salamanca and Martilli (2010) incorporated a simple building energy model (BEM) into BEP. The BEP–BEM model also allows the consideration of multiple building floors with indoor moisture and temperature estimated for each floor. Pappaccogli et al. (2020) recently performed a sensitivity analysis of the BEP–BEM model over an idealized city. Bueno et al. (2012) integrated an urban canopy model with a building energy model to quantify the interactions between building energy and urban climate. Ferrando et al. (2020) provided a more comprehensive summary of the urban building energy modeling.

Recently, some advances have been made toward more detailed modeling of various important urban features and associated impacts on climate/weather. These advances include a more accurate classification of urban land types using local climate zone (LCZ) based on the World Urban Database and Access Portal Tool (WUDAPT) (Martilli et al., 2016; Ching et al., 2018; Zonato et al., 2020), rooftop mitigation schemes like green roofs and photovoltaic panels (Sharma et al., 2016; Zonato et al., 2021), and new building drag treatments (Santiago and Martilli, 2010; Gutiérrez et al., 2015), which have been recently incorporated into the widely used Weather Research and Forecasting (WRF) model. Moreover, Wang et al. (2013b) coupled a SLUCM with an urban hydrological model to allow interactions of urban water and energy. Krayenhoff et al. (2020) and Mussetti et al. (2020) coupled the multilayer BEP model with a tree-vegetated model to account for the interactions between street trees and urban elements. Meili et al. (2020) developed an urban ecohydrological model that combines ecosystem modelling with an urban canopy scheme to account for the biophysical and eco-physiological characteristics of roof vegetation, ground vegetation, and urban trees.

### 3.2. *Small-scale building resolving models and their integration with mesoscale models*

In addition to the aforementioned urban models that have been generally applied to regional and mesoscale simulations, several other small-scale (e.g., <100 m) building-resolved models have also been developed to explicitly resolve urban building airflows at local/city scales, including microscale urban transport and dispersion models and computational fluid dynamics (CFD)-based urban models (Fernando et al., 2001; Brown, 2004; Coirier et al., 2005; Hertwig et al., 2012; Miao et al., 2013; Allegrini et al., 2015). These fine-scale urban models often require mesoscale atmospheric models to provide meteorological initial and boundary conditions (Tewari et al., 2010). The CFD-urban models can explicitly resolve building structures and different urban aerodynamic features, including street-level flow, channeling, local turbulent mixing, and downwash. Some studies also adopted large eddy simulations (LES) coupled with mesoscale atmospheric models to predict airflows and transport in urban areas (e.g., Wyszogrodzki and Smolarkiewicz, 2009; Michioka et al., 2013). One of the recent developments of LES-urban models is the development of the Parallelized Large-Eddy Simulation Model for Urban Applications (PALM; Maronga et al., 2015; Resler et al., 2017; Winkler et al., 2020), which explicitly resolves the 3D geometry of buildings, vegetation, and terrain as well as detailed radiative transfer processes in urban canopy layers and chemical processes of urban air pollutants. This allows a broad application of PALM and its variations in urban climate and pollution studies.

Moreover, to build a community urban modeling tool, Chen et al. (2011b) developed an integrated urban modelling system coupled with WRF. The WRF/urban system includes (1) multiple approaches for urban modeling, such as a simplified bulk urban parameterization, a SLUCM, a multilayer BEP model, and a sophisticated BEP–BEM model, (2) the capability of coupling to fine-scale CFD-urban and LES-urban models for transport and dispersion applications, (3) a framework to incorporate high-resolution urban land use, building morphology, and anthropogenic heating data based on the National Urban Database and Access Portal Tool (NUDAPT; Ching et al., 2009), and (4) an urbanized high-resolution land data assimilation system. Grimmond et al. (2011) and Best and Grimmond (2015) showed results from intercomparison of many existing urban models, which provided the community with an integrated perspective of weaknesses and strengths of different urban models.

### 3.3. *Urban representation in global Earth System Models*

At larger scales, urban parameterizations are rare and most Earth System Models do not explicitly represent cities. Of note, the Community Land Model (Lawrence et al., 2019), the land module of the Community Earth System Model (Danabasoglu et al., 2020), includes an urban module, CLM Urban (CLMU) (Oleson and Feddema, 2020). Different versions of the model have been used to study the urban climate at regional to global scales (Fischer et al., 2012; Oleson, 2012; Zhao et al., 2014, 2017; Cao et al., 2016). However, given its global coverage, it has several simplifications, including, but not limited to, uncertainties emerging from internal variability (Zheng et al., 2021) and biases in prescribed urban parameters like surface emissivity (Chakraborty et al., 2021b), which is also a source of uncertainty in regional models, including WRF.

### 3.4. *Modeling urban air pollution*

In addition to physical urban climate/weather models, there are also some urban air pollution models developed specifically to predict urban pollutant transport. The Urban Airshed Model (UAM) is one common urban pollution model developed more than three decades ago (McRae et al., 1982; Scheffe and Morris, 1993), which simulates physical and chemical processes of air pollutants based on the atmospheric diffusion and continuity equations. The UAM generally accounts for pollutant emissions, transport, chemical reactions, diffusion, and removal processes. Some other urban pollutant diffusion models have also been developed (e.g., Turner, 1964; Berkowicz, 2000; Carruthers et al., 2000; Karppinen et al., 2000; Soulhac et al., 2011) by including the key vertical and horizontal diffusion/dispersion and chemical reactions to specifically investigate urban air pollutant transport and evolution. Another variation of the urban pollutant model is the Urban Forest Effects (UFORE) model (Nowak and Crane, 2000), which accounts for detailed species and structure information of urban vegetation and the interactions with air pollutants (e.g., dry deposition and organic emissions from trees). In addition, the WRF-chemistry model (WRF-Chem) coupled with a UCM is used to study the urbanization impact on air quality (Zhong et al., 2018).

## 4. Impact on temperature and heat waves: Observational evidence

### 4.1. *City-level and multicity observations*

The urban climatology literature is vast, with potentially thousands of studies (Oke, 1982b; Douglas, 1983; Changnon, 1992; Arnfield, 2003; Wienert and Kuttler, 2005; Roth, 2007; Zhang et al., 2009c; Robaa, 2013; Wu and Yang, 2013; Ngie et al., 2014; Alghamdi and Moore, 2015; Santamouris, 2015; Alizadeh-Choobari et al., 2016; Wang et al., 2016, 2017a, b; Ward et al., 2016; Arsiso et al., 2018; Bader et al., 2018; Deilami et al., 2018; Salamanca et al., 2018; Wu and Ren, 2019). Observational detection of the UHI effect is one of the most consistent results in the field. This has been seen using both direct and remote measurements. The UHI intensity has been quantified for almost all major cities, particularly those in developed nations. Most cities in the world show positive SUHI (Fig. 5) and CUHI, with the main exception being cities in arid climates (Rasul et al., 2017). Both observational (Fig. 5) and modeling evidence shows that SUHI is generally smaller in drier climates and larger in moist climates (Zhao et al., 2014; Chakraborty and Lee, 2019; Manoli et al., 2019). The presence of the negative UHI, or urban cool island, in arid areas has been seen for individual cities and also in satellite-derived multicity estimates (Rasul et al., 2016; Chakraborty and Lee, 2019; Fig. 5).

### 4.2. *Surface versus canopy urban heat islands*

It is important to note that LST and air temperature, and thus SUHI and CUHI, are not identical variables. This is a necessary distinction because many of the negative impacts of the UHI effect, including heat stress, are due to high air temperature, not LST (Harlan et al., 2006; Anderson et al., 2013; Ho et al., 2016). While LST and air temperature are coupled on an annual scale, significant differences are found between the two on the diurnal and seasonal scales (Garratt, 1995; Jin et al., 1997). Moreover, since urban areas do not usually have standard weather stations (WMO, 2008), the coupling between CUHI and SUHI in urban micro-climates has been rarely quantified. A majority of observational estimates have found that while SUHI is usually stronger during daytime, the CUHI is stronger at night (Zhang et al., 2014b; Chakraborty et al., 2017; Hu et al., 2019; Venter et al., 2021). In fact, from 1950 to 2000, urbanization-induced land use changes accounted for half the decrease in the diurnal temperature range, a consequence of the higher nighttime magnitude of CUHI (Kalnay and Cai, 2003). There are also differences in the SUHI and CUHI on seasonal time scales, as shown by Chakraborty et al. (2017). Of note, the SUHI has been found to be higher and more variable during summer and smaller during winter across multiple large-scale studies (Peng et al., 2012; Clinton and Gong, 2013; Chakraborty and Lee, 2019; Fig. 5).

### 4.3. *Determinants of the urban heat island intensity*

Earlier studies on the UHI effect primarily focused on simple observations of the urban–rural thermal anomaly, providing little understanding of the mechanisms dictating the phenomenon. Moreover, proper quantification of the factors causing the UHI has been difficult owing to vast differences in city structure, background climate, and other atmospheric forcing (Arnfield, 2003). Oke (1969) suggested a need for a proper rational understanding of the factors responsible for the UHI and outlined several possible reasons for the UHI effect (Oke, 1982a). A recent review of 88 papers on the UHI of 101 Asian cities found several major determinants of the UHI commonly examined in the literat-

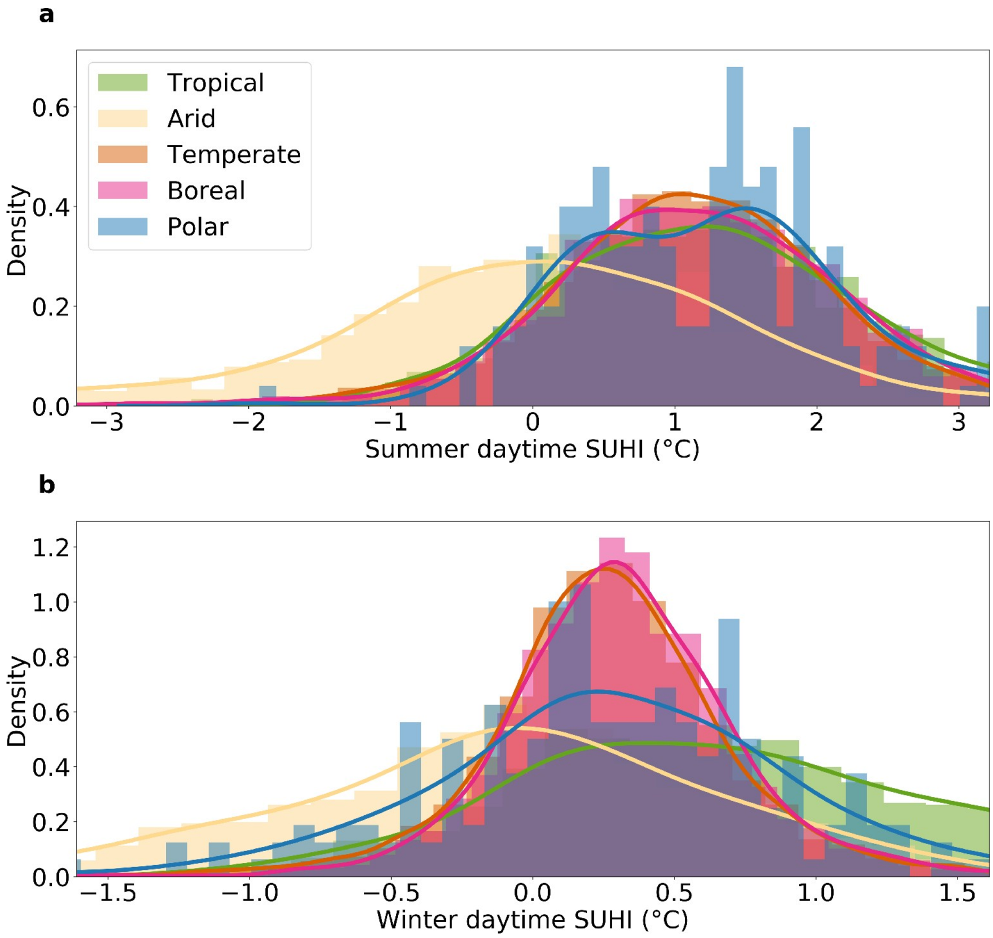

**Fig. 5.** Distribution of daytime surface urban heat island (SUHI) for (a) summer and (b) winter for over 10 000 urban clusters across the globe and across different Koppen-Geiger climate zones. Based on the Yale Center for Earth Observation (YCEO) Surface Urban Heat Island Dataset [modified from Chakraborty and Lee (2019)].

ure, including background climate conditions, city size, rainfall, cloud cover, and coastal feedback (Santamouris, 2015). Moreover, due to the wide variety of fields involved in UHI research including, but not limited to, geosciences, meteorology, climatology, environmental science and engineering, geography, ecology, urban planning and architecture, etc., there has been an inconsistency in the terminologies used in the literature (Oke, 2006).

A few large-scale studies have investigated the possible causes of the SUHI. A global study of 419 cities found higher daytime values, with no significant correlation between daytime and nighttime surface UHI (Peng et al., 2012). They also analyzed the statistical association of different biophysical parameters with the LST difference. The nighttime SUHI was correlated with urban–rural difference in shortwave reflectivity (or albedo) and night light, while the daytime values showed associations with urban–rural difference in vegetation cover. The influence of this urban–rural difference in vegetation cover on the SUHI, as well as CUHI, has also been seen in other large-scale studies (Chakraborty and Lee, 2019; Paschalis et al., 2021; Venter et al., 2021). The aerodynamic smoothness of cities can also influence the UHI by modulating convective cooling (Zhao et al., 2014), which is seen during summer days over 342 urban clusters in Europe (Venter et al., 2021), providing observational evidence of previously modeled results. The coupling between UHI and urban aerosols has also been examined in observational studies, with various degrees of control found through the radiative effect of aerosols (Li et al., 2018b; Zheng et al., 2018; Ulpiani, 2020; Chakraborty et al., 2021a). More recently, studies have explored not just the difference in vegetation cover in cities, but also changes in plant physiological and biophysical characteristics in urban micro-climates and how they relate to local urban thermal anomalies (Meng et al., 2020; Paschalis et al., 2021). As a bulk control, multiple studies have shown that the SUHI intensity is associated with the background climate in which a city exists (Zhao et al., 2014; Chakraborty and Lee, 2019; Li et al., 2019; Manoli et al., 2019; Fig. 5).

### 4.4. *Intra-urban variability in urban temperatures*

Due to the spatial continuity of satellite-derived observations, it has been easier to study intra-urban variability in urban LST (and thus the SUHI). Large spatial variability has been found, generally controlled by the degree of urbanization and the fraction of impervious surfaces (Hass et al., 2016; Wang et al., 2017a; Naughton and McDonald, 2019; Chakraborty et al., 2020). Of note, the SUHI literature has recently focused on systematic disparities in LST and SUHI intensity by race and income across multiple cities (Chakraborty et al., 2019, 2020; Hoffman et al., 2020; Hsu et al., 2021; Benz and Burney, 2021). Although similar multicity studies for CUHI and to examine the overall heat stress across population groups are difficult, results from single-city studies suggest higher urban heat stress in poorer neighborhoods, at least in the United States (Harlan et al., 2006; Voelkel et al., 2018).

### 4.5. *Long-term changes in the urban heat island intensity*

Although recent studies have investigated the long-term changes in the SUHI for individual cities, multicity observational estimates are still sparse (Scott et al., 2018; Chakraborty and Lee, 2019; Yao et al., 2019). Of note, for developed regions, the general trend seems to be a stagnation of the SUHI intensity or a slight reduction. However, cities in tropical areas in developing countries have shown an increasing trend in SUHI intensity, particularly at night (Chakraborty and Lee, 2019). Though different studies do not agree on the magnitude of this change (Chakraborty and Lee, 2019; Yao et al., 2019), it is important to note that differences in both methodology and sample sizes (with Yao et al. (2019) focusing on larger cities) could contribute to this.

Regionally, long-term air temperature observations over China have suggested an increase in the frequency of extreme heat events due to urbanization (Yang et al., 2017). As also noted in Yang et al. (2017), estimating the actual long-term change in UHI (both SUHI and CUHI) is difficult due to the urban contamination of 'rural' sites due to urban expansion, which can also impact regional climate beyond the urban boundary (Zhou et al., 2004; Tao et al., 2015; Gough, 2020; Güneralp et al., 2020). In fact, long-term climatological records can also be affected by urban signatures (Li et al., 2004; Hua et al., 2008; Hausfather et al., 2013; Lin et al., 2016). Most long-term studies on the UHI effect do not consider the effect of the conversion of stations from rural to urban on local climate, and the class of the stations remains fixed throughout the analysis period. In China, rapid urbanization can lead to a transition of a station from rural to urban within a short period. Yang et al. (2011) dynamically categorized urban and rural stations in east China based on the DMSP/OLS nighttime light data of 1992–2007 and found an evident UHI effect on warming trends. Follow-up studies dynamically classified the stations into urban and rural types based on the time-varying land use data (Liao et al., 2017; Lin et al., 2018, 2020). Using an optimal fingerprinting technique, Sun et al. (2016a) suggested that urbanization effect accounted for about a third of the observed warming in China.

## 5. Impact on temperature and heat waves: Modeling results

### 5.1. *Urban heat island intensity*

Modeling urban temperature and the UHI by simulating their complex interplay at different time-space scales has been valuable in understanding the underlying governing factors (Chen et al., 2004; Kusaka and Kimura, 2004; Lo et al., 2007; Lin et al., 2008b; Salamanca et al., 2011; Rajagopalan et al., 2014; Sun and Augenbroe, 2014; Magli et al., 2015; Kim et al., 2016; Zhao et al., 2018; Mughal et al., 2019; Wang and Li, 2021). Salamanca et al. (2011) compared all the urban canopy schemes in Houston and observed a temperature rise (~2°C) in higher density areas, indicating that the complex canopy schemes are critical in evaluating energy consumption of air conditioning at city scales. Similar results have also been observed in China, where more realistic urban representations are effective in better estimating temperature and wind speed (Liao et al., 2014). However, some studies also reported insignificant improvements when using more complex models due to the lack of reference data (Karlický et al., 2018). Moreover, the temporal and spatial scales of the targeted research question should determine the sophistication of models employed, given that model approximations are scale dependent. For instance, building energy modules are needed when UHI interactions are aimed at canyon scale, i.e., ~10 meters (Sun and Augenbroe, 2014; Magli et al., 2015). Conversely, only multilayer UCM urban canopy models should be employed for representing UHI interactions at the urban canopy scale (~10 km) (Kusaka and Kimura, 2004; Kondo et al., 2005; Holt and Pullen, 2007).

With advancements in computational capabilities, large-eddy simulations are also utilized to solve Computational Fluid Dynamics and aid in understanding UHI distribution and associated circulations (Wang and Li, 2016). These models are useful for simulating UHI-induced urban flows (Catalano et al., 2012), mesoscale circulations due to horizontal UHI heterogeneity (Zhang et al., 2014a; Zhu et al., 2016), and UHI–sea breeze interactions (Shen et al., 2018). These urban modules are often accompanied by a mesoscale weather model to simulate the impact of UHI on precipitation intensity (Lin et al., 2008a, 2011), local circulation (Lo et al., 2007), sea breeze interaction (Shen et al., 2018), background warming (Chen et al., 2014), etc., at large spatiotemporal scales. In addition, statistical modeling approaches are also employed as an alternative to numerical models for explaining the UHI phenomenon. However, these approaches have limited ability in explaining the underlying mechanisms, which can be examined using physics-based models (Su et al., 2012; Doick et al., 2014; Ho et al., 2014; Ivajnšič et al., 2014; Quan et al., 2014; Guo et al., 2015; Kotharkar et al., 2019).

Overall, modeling studies agree on the diurnal pattern of dominance of CUHI during nighttime in most cities. Higher values of ground heat fluxes in urban surfaces are responsible for strengthening this primarily nocturnal phenomenon (Van Weverberg et al., 2008; Bohnenstengel et al., 2011; Yang et al., 2012). Similar results have been found in Greece (Giannaros et al., 2013) and Singapore (Mughal et al., 2019), with an exception in a complex topographic region where dominating sensible heat fluxes led to high daytime CUHI (Lin et al., 2008b). The diurnal patterns for SUHI are also related to those of CUHI (Yang et al., 2012; Giannaros et al., 2013). However, the peak-value timings for SUHI and CUHI are diverse (Yang et al., 2012; Chakraborty et al., 2017; Venter et al., 2021). This finding is different from that based on satellite observations (discussed above), where SUHI is generally larger during daytime. Future studies should aim to better understand this discrepancy.

In terms of seasonal variations, similar patterns for CUHI and SUHI have been found across the seasons in China, but differences between dry and wet seasons have been observed over a subtropical city (Chakraborty et al., 2017). High solar elevation and extensive vegetation cover (and thus, larger urban–rural differential in vegetation cover) resulted in maximum UHI values in summer and minimum values in winter (Zhang et al., 2010). However, seasonal variations are not dependent only on the aforementioned factors. In Mexico, the enhancement of atmospheric stability in the dry season is directly interlinked with high values of nighttime SUHI. On the other hand, daytime SUHI is influenced by vegetation fraction diversity between rural and urban areas. Atmospheric stability has also been found to influence nighttime CUHI, but daytime values display seasonal consistency, suggesting a lack of dependence on vegetation fraction (Cui and De Foy, 2012). Similar consistent patterns of daytime CUHI have been observed in London (Wilby, 2003; Zhou et al., 2016). Zhou et al (2016) found a hysteresis-like seasonality in SUHI, but not in CUHI, for the city of London.

### 5.2. *Urban heat stress and extremes*

Climate change has increased the frequency, duration, intensity, and spatial extent of extreme heat episodes (Meehl and Tebaldi, 2004). However, the extent to which urban warming has affected the extreme temperature trends remains an open question (Ren and Zhou, 2014). In the United States, DeGaetano and Allen (2002) suggested that trends in high maximum and minimum temperature extremes across the country were strongly influenced by urbanization. The trends of extreme temperatures were more prominent over suburban stations that have undergone greater rates of urban expansion than the steady metropolitan core areas (Sen Roy and Yuan, 2009). In addition, the increasing trend of heatwave episodes was higher in more sprawling, compared to compact, metropolitan regions (Stone et al., 2010). Recent studies have focused on China, which has experienced rapid economic development and urbanization over the past 40 years. All these studies at regional scales identified significant urbanization contribution to the increasing trends for both extreme temperature indices (Ren and Zhou, 2014; Luo and Lau, 2017; Yang et al., 2017; Lin et al., 2018, 2020; Wu et al., 2020, 2021) and human-perceived heat stress (Luo and Lau, 2017, 2021; Wang et al., 2021a), with the urbanization contributions generally ranging from 20% to 50%. Based on an optimal fingerprinting method, urbanization contributed one-third of the increase of nighttime extremes (Sun et al., 2019) and 29.6% of human-perceived heat wave intensity (Kong et al., 2020). Moreover, the contribution of urbanization to the increasing trends of heat waves and extreme heat stress are greater in wet climates (Liao et al., 2018; Luo and Lau, 2021). Summertime heat stress increased faster than air temperature in urban areas across China (Luo and Lau, 2018; Wang et al., 2021a).

The response of UHI to extreme heat episodes has also been modeled by many studies. In general, simulated CUHI values during a heat wave have intensified extensively relative to pre-heat wave conditions, mostly due to the lower moisture and reduced wind speed in urban areas during the heat wave. Moreover, the intensity monitored during nighttime was significantly higher, validating its typical nocturnal predominance (Li and Bou-Zeid, 2013; Zhao et al., 2018; Ao et al., 2019; He et al., 2020). However, coastal cities where sea-breeze circulations are dominant exhibited higher daytime responses of UHI to heat waves. The contrasting trends observed during sea breezes and land breezes are a probable explanation for this difference (Ao et al., 2019). The heat wave effect on the enhancement of SUHI has been reported by Li and Bou-Zeid (2013). Alternatively, a few cities in the US have not shown any clear association between CUHI and heat waves. A huge difference in soil moisture between industrialized and suburban land cover during pre-heat wave conditions is known. This difference does not change during heat waves, and as a result, a stable moisture deficit is maintained between the urban–suburban gradient (Ramamurthy and Bou-Zeid, 2017). No synergistic interactions between CUHI and heat waves were observed over Singapore, suggesting that the associations may be small in tropical cities compared to midlatitude cities (Chew et al., 2021). Note that positive correlations for SUHI and CUHI during heat wave conditions have been seen in modeling studies over temperate cities in the US (Zhao et al., 2018), China (Chen et al., 2014), and Europe.

UHI-associated micrometeorological changes have adverse consequences on human thermal comfort (Yang et al., 2019a). Furthermore, energy consumption has intensified with increased UHI in Vienna (Berger et al., 2014), Modena (Magli et al., 2015), Delhi (Kumari et al., 2021), and multiple cities across the US (Sun and Augenbroe, 2014). Indices such as mean radiant temperature (MRT), physiologically equivalent temperature (PET), universal thermal climate index (UTCI), and percentage mean vote (PMV) have been employed for quantitative estimation of UHI-associated heat- and energy-stress in Colombo, Phoenix (Emmanuel and Fernando, 2007), Stuttgart (Ketterer and Matzarakis, 2014), Rotterdam (Van Hove et al., 2015), De

Bilt (Taleghani et al., 2015), Bahrain (Radhi et al., 2013, 2015), and Ho Chi Minh City (Doan et al., 2016).

### 5.3. *Urban heat mitigation*

Several modeling studies have focused on testing the efficiency of different approaches to mitigating the UHI (Sun et al., 2016b; Zhao et al., 2017). Urban greening (more vegetative cover) has been found to be quite effective in various cities (Ambrosini et al., 2014; Ketterer and Matzarakis, 2015; O'Malley et al., 2015; Sharma et al., 2018; Tsilini et al., 2015; De Munck et al., 2018; Zonato et al., 2021). Specifically, a significant reduction in surface temperature (5°C) and PET (average of 2.2°C) has been observed in Chania (Tsilini et al., 2015) and Stuttgart (Ketterer and Matzarakis, 2015), respectively. Tehran has witnessed a reduction in nocturnal UHI by 0.92°C due to urban greening (Sodoudi et al., 2014), while a 10% increment of greening in Toronto can reduce air temperature and PET up to 0.8°C (Wang et al., 2015c). A positive correlation of urban greening and sky view factor (SVF) has been seen in Hong Kong, where a high SVF resulted in a reduction in air temperature by 1.5°C. On the other hand, surface brightening (use of high albedo materials; also see Akbari et al., 2009; Oleson et al., 2010; Scherba et al., 2011; Kolokotroni et al., 2013; Li et al., 2014; Ma et al., 2014; Santamouris, 2014) has also been effective in independent case studies over Singapore where air temperature reductions of 2.5°C (Priyadarsini et al., 2008) and 1.3°C (Mughal et al., 2020) have been calculated. The usage of high albedo roof and pavement materials led to cooling of 1.7°C and 6.5°C in Venice (Peron et al., 2015) and Serres (Dimoudi et al., 2014), respectively. However, mitigating the UHI through albedo management has been found to increase ground-level air pollution in modeling studies (Chen et al., 2018; Zhong et al., 2021). Some studies argued that the net effectiveness strengthens when amalgamation of multiple approaches are implemented (Zhao et al., 2017). For instance, overall cooling effects of 3.0°C (Sodoudi et al., 2014) and 1.1°C (Peron et al., 2015) in their respective study regions have supported these arguments. Along with albedo management, building urban water bodies (O'Malley et al., 2015; Radhi et al., 2015) and changes in urban forms (Emmanuel and Fernando, 2007; Priyadarsini et al., 2008; Taleb and Abu-Hijleh, 2013; Rajagopalan et al., 2014; Zhao et al., 2014; Lai et al., 2019) are also proposed as UHI-mitigating measures.

Urban greening, depending on the species of vegetation used, can be effective for overall temperatures and mildly effective or not effective at all for heat stress (Hass et al., 2016). Note that during winter, if trees (deciduous) shed their leaves, they have a lower cooling potential during this season, which is better for most cities in colder climates. Greening can also effectively reduce wind chill (Giometto et al., 2017) and provide shading (Armson et al., 2012), which white roofs cannot do. However, there is a potentially higher cost of tree planting and maintenance plus water requirements, which would become even more expensive in the future due to water stress in many cities around the world (He et al., 2021). Moreover, other urban structures can also provide shade, with many being more effective than urban vegetation (Middel et al., 2021).

## 6. Impacts on wind, boundary layer structure, clouds, and air pollution

Urbanization's impact on the atmosphere extends beyond the urban canopy layer. Above the canopy layer, the influence of urbanization remains important. As mentioned earlier, another type of UHI is the boundary layer UHI (Oke, 1995), which describes the higher air temperature in the urban boundary layer compared to its rural counterpart. This boundary layer (about 1–2 km above the ground) UHI effect has been observed (Oke and East, 1971) and simulated (Uno et al., 1989; Martilli, 2002). However, the urban boundary layer in general remains understudied (Barlow, 2014), especially when compared to the urban canopy layer, for which there is a wealth of weather station and satellite data. For instance, the urban boundary layer heat budget has not been systematically quantified (Wang and Li, 2019). The impact of urbanization also goes beyond temperature, as urbanization also affects wind patterns and circulations, the boundary layer height, moisture conditions, clouds, and precipitation.

### 6.1. *Urban wind patterns and interactions with mesoscale circulations*

Depending on the strength of the ambient wind, the wind pattern in the urban boundary layer can be classified as an urban dome (when the ambient wind is weak or absent) or an urban plume (when the ambient wind is strong) (Zhu et al., 2016; Oke et al., 2017; Omidvar et al., 2020; Fig. 6). The wind field in the urban canopy layer and roughness sublayer is more complicated and is greatly affected by the details of building morphology (Louka et al., 2000; Oke et al., 2017). As a whole, urban areas typically have larger roughness elements (mostly buildings) than the surrounding rural areas (Bottema, 1997). Due to this dynamic effect, wind speed tends to be reduced as the air moves towards urban areas (Zhu et al., 2016) or as urbanization continues (Hou et al., 2013). However, urban areas often have higher sensible heat fluxes than rural areas, which may induce a self-contained UHI circulation (i.e., urban dome). The UHI thermal circulation might result in an acceleration of air flow as it approaches urban areas. Observational evidence for both dynamic (roughness-induced reduction in wind speed) and thermodynamic (thermally-direct circulation driven acceleration of winds) effects on winds over urban areas have been presented in Bornstein and Johnson (1977).

The interactions between urban-induced circulations with meso- to regional-scale circulations like mountain–plain circulations and land–sea or lake breezes are the subject of active research. Using radar observations, Keeler and Kristovich (2012) explored how the UHI effect of Chicago affects lake breezes and found that deceleration of lake-

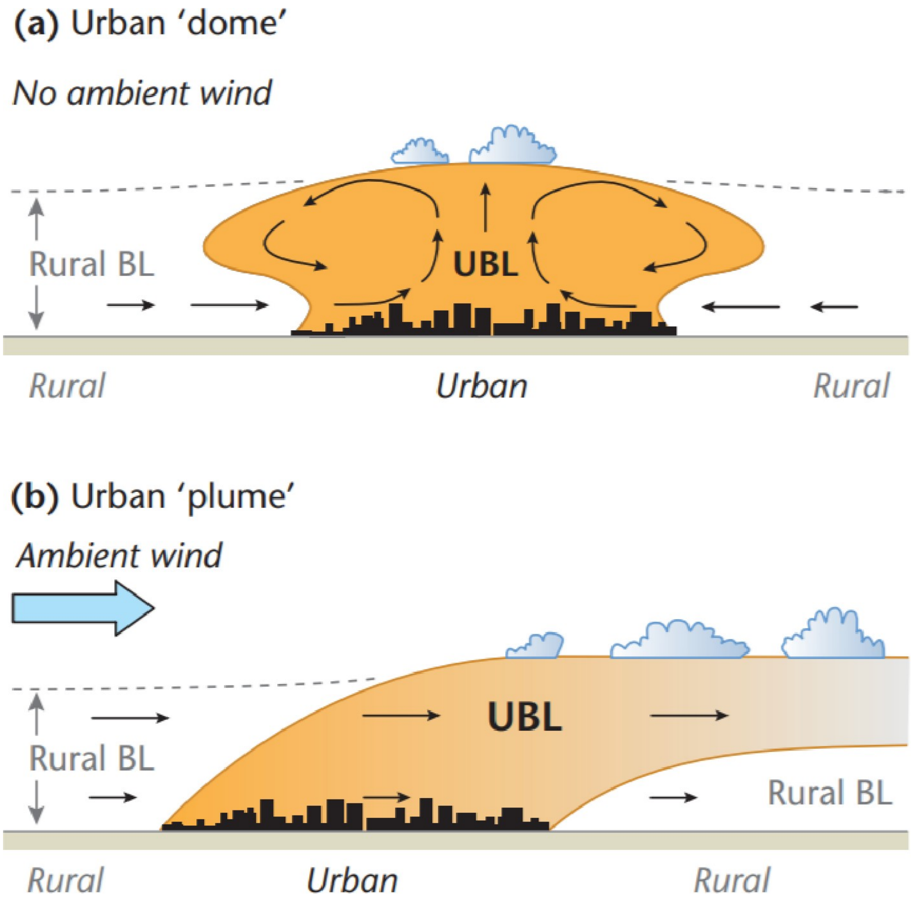

**Fig. 6.** Typical overall form of urban boundary layers at the mesoscale: (a) urban "dome" when regional flow is nearly calm, and (b) urban internal boundary layer and downwind "plume" in moderate regional airflow [Reprinted from Fig. 2.12 in *Urban Climates* by Oke et al. (2017), © 2017 Cambridge University Press. Reproduced with permission of The Licensor through PLSclear.].

breeze frontal movement inland of Chicago's urban center was highly correlated with the maximum nighttime UHI magnitude. Numerical models also provide important insights on how urban forcing interacts with the broad geographical features in shaping the wind field. An early study by Yoshik Ado (1992) investigated the daytime urban effect and its interaction with the sea breeze using a two-dimensional hydrostatic model. Recent studies have extended these idealized investigations using three-dimensional large-eddy simulation models (Jiang et al., 2017). In terms of weather modeling, Miao et al. (2009) investigated the interactions between the mountain–valley flows and UHI circulations over the Beijing area using the WRF model and observational data. Chen et al. (2011a) and Ribeiro et al. (2018) examined the interactions between the sea breeze and urban forcing in Houston, Texas, US and Sao Paulo, Brazil, respectively. Also using the WRF model, Miao et al. (2015) studied how urbanization, topography, and proximity to the sea together shape the wind field over the Beijing–Tianjin–Hebei area in China. These studies underscore the multiscale nature of the wind fields over urban areas and demonstrate that the urban effect on air flows, while being important, is compounded by the broad geographical features and associated circulations.

### 6.2. *Turbulence and mixing*

In addition to the mean wind field, the impact of urbanization on turbulence and turbulent transport is also important. Field studies of urban effects on turbulence have relied on aircraft (Hildebrand and Ackerman, 1984) and sonic anemometers (Rotach, 1993a, b). Aircraft can provide data across the entire urban boundary layer and beyond (Zhang et al., 2019b, 2020a, b), while sonic anemometers usually give results only in the canopy layer and roughness sublayer, except when they are mounted on balloons (Uno et al., 1988, 1992). Comprehensive reviews of turbulence over cities are provided by Roth (2000) and Barlow and Coceal (2009), the latter focusing on turbulence in the roughness sublayer.

Turbulent kinetic energy is generated by both shear and buoyancy forces. Given that urban areas are characterized by increased roughness and sensible heat flux, the turbulent kinetic energy, especially the vertical component, is often higher in cities. The stronger turbulent kinetic energy leads to stronger mixing in the boundary layer and stronger entrain-

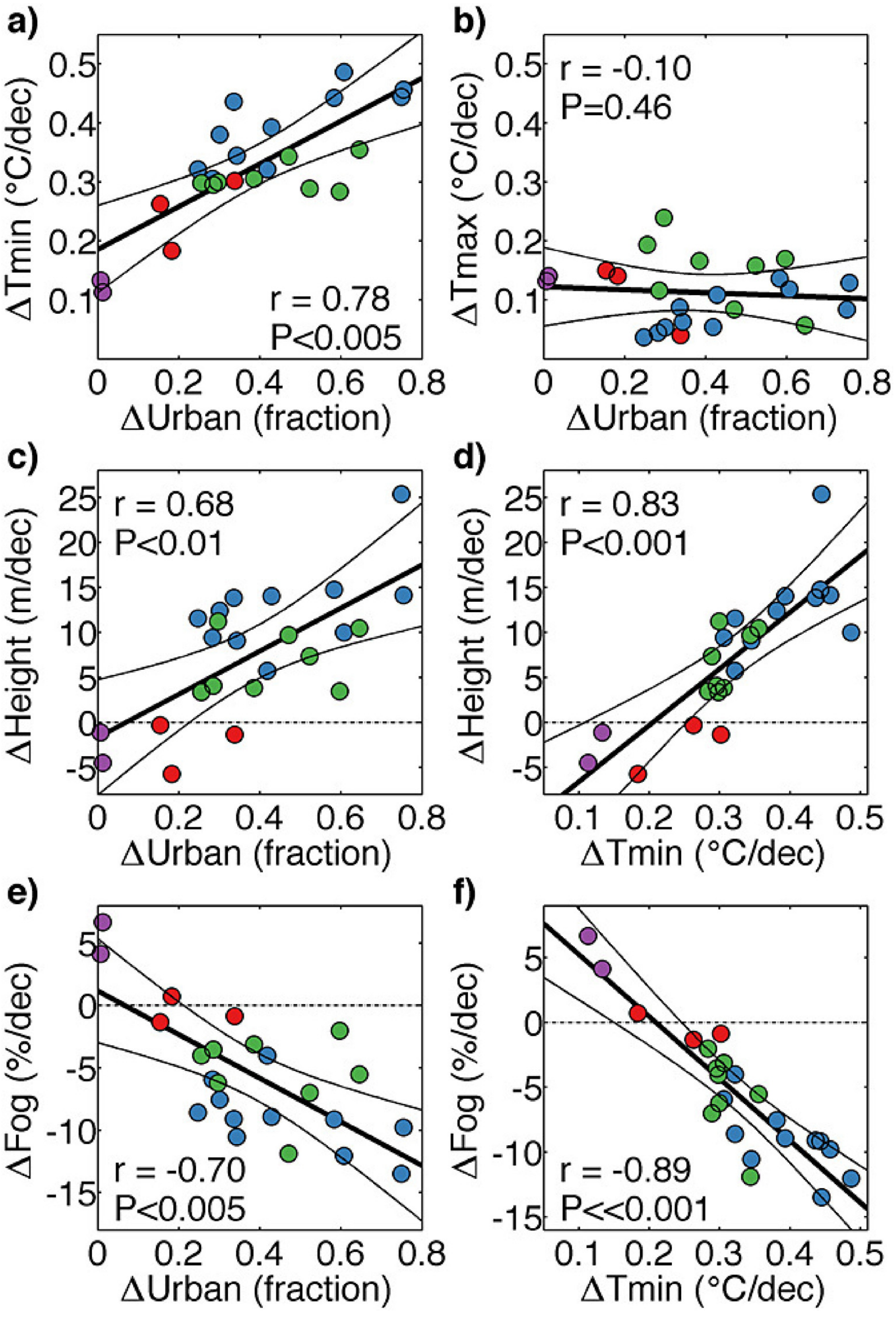

**Fig. 7.** (a–f) Airfield-specific relationship between changes in temperature, urban cover, 0700 (LST) stratus cloud base height, and 0700 (LST) fog frequency. Each dot represents one of 24 Coastal Southern California (CSCA) airfields, and dot colors indicate subregion (red: SB, blue: LA, green: SD, and purple: Islands). Bold black lines: regression lines. Correlation significance values (*P* values) and 95% confidence intervals around regression lines reflect uncertainties due to spatial autocorrelation and measurement errors [Reproduced from Williams et al. (2015). ©2015. American Geophysical Union.].

ment, which further results in a faster-growing and deeper boundary layer over urban areas during daytime. This has been well observed and documented (Spanton and Williams, 1988; Dupont et al., 1999; Angevine et al., 2003). Interestingly, the contrast of turbulent kinetic energy and mixing between urban and rural areas has stronger impacts on the boundary layer structure (e.g., the inversion strength, the boundary layer height) at night (Clarke, 1969; Godowitch et al., 1985; Dupont et al., 1999) and/or in the winter season (Sang et al., 2000), when the rural land experiences stable conditions while the urban land still generates positive sensible heat flux (Fig. 7).

### 6.3. *Moisture and clouds*

Although the urban atmosphere is usually hotter and thus has a higher water demand (Zipper et al., 2017), evapotranspiration in urban areas is often lower than that in rural areas because of the lack of soil moisture and vegetation. As a result, the daytime urban boundary layer often has a lower amount of water vapor, especially in the lowest few hundred meters (Tapper, 1990). This, together with the higher air temperature, results in a lower relative humidity in urban areas, or the UDI effect. Similar to the UHI, the presence of the UDI has been seen in several studies (Holmer and Eliasson, 1999; Kuttler et al., 2007; Moriwaki et al., 2013; Du et al., 2019; Wang et al., 2021b). This has also generally been linked to the lack of urban vegetation and pervious surfaces in cities. Of note, Hass et al. (2016) found that the urban areas with trees in Knoxville, Tennessee in the United States had higher magnitudes of heat index than downtown areas and areas with less trees, due to the competing impact of air temperature and relative humidity on heat stress.

Given the relation between the cloud base height (e.g., characterized by the lifting condensation level) and the relative humidity, all else remaining equal, urban areas are expected to have higher cloud bases, which has been widely observed (Semonin, 1981; Williams et al., 2015). Higher cloud tops over urban areas are also observed in the METROMEX campaign (Braham and Wilson, 1978). In terms of cloud frequency and duration, observational studies suggest that low-level clouds (like shallow cumulus clouds) are more frequently formed over cities and last longer during the daytime (Inoue and Kimura, 2004; Theeuwes et al., 2019). While this might be counterintuitive considering that urban surfaces release a lower amount of moisture, it is the stronger mixing caused by the higher sensible heat flux in urban areas that leads to the stronger vertical transport of moisture and thus extended periods of cloud coverage and stronger convection over urban areas (Zhu et al., 2016).

### 6.4. *Air pollution*

Urban areas are important sources of aerosols and their corresponding precursor gases [e.g., nitrogen oxides ($NO_x$), sulfur dioxide ($SO_2$), organic carbon (OC), and black carbon (BC)] due to intensive human activities and industrial productions (Wang et al., 2009; Yu et al., 2012). Urbanization-induced UHI and aerosols have opposite effects on air quality. Using the coupled WRF–Chem–Urban regional model, Zhong et al. (2018) investigated the individual and combined UHI and aerosol effects induced by urbanization on air quality. They found that while the UHI effect destabilizes the lower atmosphere and increases ventilation over the urban area (thus favoring the dispersion of pollutants from urbanized areas to their immediate vicinities), increased aerosol emissions from cities outweigh the UHI effect, resulting in a net increase (i.e., accumulation) in surface pollutants. Since urban areas can have higher pollutant concentrations than their surroundings (Han et al., 2020), several studies have examined the interactions between the urban pollution island (UPI) and the UHI intensity (Ulpiani, 2021). Li et al. (2018b) found a negative relationship between the UHI and the UPI at nighttime for Berlin. Similarly, for Delhi, Pandey et al. (2014) found strong negative relationships between UHI and aerosol optical depth (AOD) at the monthly scale. Cao et al. (2016) showed that the background climate had an influence on the interactions between air pollution and the SUHI, with nighttime SUHI increasing with higher aerosol loading for arid cities due to the longwave radiative forcing of coarser mineral dust aerosols. Recently, Chakraborty et al. (2021a) tested the impact of decreased aerosol loading during COVID-19 lockdowns over north India on the SUHI intensity and found the impact to be negligible compared to the contribution from changes in surface vegetation.

## 7. Impact on precipitation and extreme storms

Since the pioneering work of Horton (1921), which noticed a tendency for thunderstorm generation over some cities rather than their immediate surrounding country in 1921, great efforts have been made to investigate the impact of urbanization on precipitation, or the so-called urban rain effect (URE), such as the investigation of the controversial "La Porte Anomaly" in northwestern Indiana about 50 km east of the Chicago–Gary industrial region (Changnon, 1968; Ogden, 1969; Landsberg, 1970; Ashby and Fritts, 1972; Changnon, 1973, 1980) and the famous Metropolitan Meteorological Experiment (METROMEX) undertaken at St. Louis, Missouri in the 1970s (Changnon et al., 1971, 1976; Huff and Changnon, 1972; Schickedanz, 1974; Huff, 1975). Due to the availability of more completed and advanced observation systems (e.g., radar and satellite) and the development and popularity of more sophisticated models, more studies have been carried out to investigate URE theoretically and practically for cities around the globe in the recent decades (Shepherd et al., 2002; Chen et al., 2007; Simpson et al., 2008; Halfon et al., 2009; Hand and Shepherd, 2009; Shem and Shepherd, 2009; Niyogi et al., 2011; Ashley et al., 2012; Kusaka et al., 2014; Zhong et al., 2015, 2017; Yang et al., 2016; Liang and Ding, 2017; Wu et al., 2019; Fan et al., 2020; Marelle et al., 2020; Georgescu et al., 2021). However, unlike the UHI effect, which is relatively well studied and understood, the knowledge about URE is still evolving due to the involvement of many dynamic, thermodynamic, and microphysical processes and the temporospatial discontinuity of precipitation. Currently, the following four major mechanisms are believed to be associated with URE (Huff and Changnon, 1973; Han et al., 2014a; Mitra and Shepherd, 2015; Liu and Niyogi, 2019).

### 7.1. *UHI–thermal perturbation of the boundary layer*

UHI acts as a low-level heat source and induces convergence at low levels and divergence aloft in the urban area, forming a "dome-shaped" upper boundary, which is named the UHI circulation (UHIC) (Fan et al., 2017). Under the effect of environmental wind advection, the convergence center is diverted to downwind areas (Han and Baik, 2008). The upward motion associated with the downwind convergence may facilitate the initiation of moist convection under favorable conditions and thus enhance precipitation downwind of a city (Baik et al., 2001; Han and Baik, 2008). The UHI-associated boundary layer perturbation is thought to be the dominant factor for precipitation enhancement in urban downwind areas in some studies (Thielen et al., 2000; Rozoff et al., 2003; Shem and Shepherd, 2009). However, it is noteworthy that URE is not only related to the intensity and horizontal structure of UHI (Han and Baik, 2008; Miao et al., 2011) but is also influenced by environmental conditions. Stronger downwind updrafts are necessary for convective storm initiation under less favorable thermodynamic conditions (Baik et al., 2001). In addition, UHIC itself is also influenced by environmental factors. By conducting 2D numerical experiments, Baik et al. (2001) found that downwind updrafts decreased as environmental wind speed increased, thus suppressing convection initiation and enhancing downwind precipitation. Moreover, Baik et al. (2007) showed that when the boundary layer was more stable, both the strength and the vertical extent of UHIC-induced downwind updrafts decreased. Therefore, further investigations under various environmental conditions are necessary to fully understand the effect of UHIC on URE.

### 7.2. *Enhanced convergence due to increased urban surface roughness*

The urban vertical structure of cities generally increases their aerodynamic roughness, resulting in the enhancement of surface drag and wake turbulence. Urban-increased drag and turbulence reduce wind speeds more significantly than the drag and turbulence found in surrounding rural areas. If these effects occur at a large spatial scale, a local convergent wind field may be generated under the impact of the Coriolis force (Collier, 2006; Shem and Shepherd, 2009). Convergence causes the rise of the inflowing air, and when the inflow possibly reaches the level of free convection (LFC), deep convection is triggered. Therefore, enhanced convergence due to increased urban surface roughness can enhance precipitation in some areas of a city. Thielen et al. (2000) suggested a positive relationship between urban roughness length and downwind precipitation based on 2D simulation experiments. Escourrou (1991) estimated that the effect of roughness enhanced precipitation downwind of the Paris downtown area by 10%. However, through sensitivity simulations of a deep convection event over St. Louis, Missouri, Rozoff et al. (2003) demonstrated that the surface roughness-induced convergence zone was on the windward side of the city and was insufficient to invigorate deep convection. In contrast, it slowed wind and dampened downwind convergence caused by UHIC. Due to the lack of extensive studies quantifying the isolated contribution of surface roughness on URE, we cannot make any robust conclusions about the relative importance of this mechanism compared to other pathways. Further studies are necessary to investigate the details.

### 7.3. *Modification of existing precipitating systems by the urban landscape*

The presence of buildings and the generation of UHIC over urban areas may act as barriers altering the structure and propagation of precipitating systems passing over a city. Based on high-resolution WRF simulations of a heavy summer rainfall event in Beijing, China, Miao et al. (2011) demonstrated that urban land cover might cause a squall line to split into convective cells over the urban area. This does not necessarily lead to reduced precipitation but may increase it if the water vapor availability is high. The increase in average precipitation downwind of cities as well as extreme precipitation around cities are found from in situ measurements (Huff and Changnon, 1973; Kishtawal et al., 2010). By analyzing radar characteristics of 91 summertime thunderstorms around Indianapolis, Indiana, Niyogi et al. (2011) found that some storms bifurcated when approaching the urban region and remerged downwind of the city. In comparison, Lin et al. (2021) and Ganeshan and Murtugudde (2015) argued that enhanced urban-induced convergence in the urban area and around the urban–rural boundaries could attract convective storms towards a city. It is apparent that the alteration of the structure and propagation of existing precipitating systems affect rainfall over urban areas (Bornstein and Lin, 2000; Burian et al., 2004, Dou et al., 2015); however, few studies have tried to quantify whether this causes decreased or increased precipitation. A previous satellite-based estimate found increases in monthly warm-season rainfall both downwind and over cities in the US (Shepherd et al., 2002).

### 7.4. *Urban aerosol effect on local precipitation*

Urban aerosols can affect precipitation through two different pathways. The first is through the alteration of urban radiation balance (aerosol–radiation interaction). Aerosols scatter and absorb radiation and thus cool and stabilize the atmosphere, somewhat opposing the thermodynamic influence of the UHI (Jin et al., 2010). It is important to note that aerosol-induced cooling may suppress UHI-induced URE. The second pathway is based on the role of aerosol particles as cloud condensation nuclei (CCN) in cloud formation (aerosol–cloud interaction). However, whether urban aerosols inhibit or enhance precipitation depends on many factors (e.g., cloud type, aerosol size, aerosol concentrations, and environmental conditions), and the involved mechanisms are still being debated (Rosenfeld, 2000; Soriano and de Pablo, 2002; Borys et al., 2003; Givati and Rosenfeld, 2004; Rosenfeld and Givati, 2006; Lynn et al., 2007; Bell et al., 2008; Choi et al., 2008; Khain et al., 2008; Rosenfeld et al.,

2008; Lacke et al., 2009; Ntelekos et al., 2009; Svoma and Balling, 2009; Han et al., 2012; Fan et al., 2020; Lin et al., 2021). For example, Svoma and Balling (2009) found that winter precipitation frequency in the Phoenix metropolitan area was inversely related to aerosol concentrations. Bell et al. (2008) found enhanced precipitation on high aerosol concentration days in summer in the southeast United States. Carrió and Cotton (2011) and Carrió et al. (2010) showed that downwind precipitation efficiency first increased and then decreased with increasing aerosol concentrations in Houston. However, Jin et al. (2005) observed no clear relationships between aerosol concentrations and summertime rainfall in New York and Houston. Anthropogenic aerosols have been shown to increase peak precipitation rate and frequency of large rainfall events over Houston (Fan et al., 2020). Using the coupled WRF–Chem–Urban regional model at convection-permitting resolution, Zhong et al. (2015) and Zhong et al. (2017) simulated the individual and combined UHI and aerosol effects induced by urbanization on summer storms in two megacity clusters of China. While the UHI effect increases heat stress and storm frequency in summer, urbanization-induced aerosols, depending on the synoptic conditions of the storm event, tend to suppress the UHI effect on precipitation, leading to an overall suppression effect of urbanization on precipitation. These controversial results urge further studies for a comprehensive understanding of cloud–aerosol interactions, particularly over urban areas.

The above four pathways do not work alone but generally take effect simultaneously. A conceptual schematic based on previous studies is shown in Fig. 8, which highlights the roles of each process in URE and their interactions with other factors. However, the schematic will evolve as the understanding of URE improves with further studies in the future.

Due to the synergistic impact of the above mechanisms (Shepherd, 2013) and their uncertainties and interactions with various environmental factors (e.g., moisture availability, topography, and sea breezes) (Chen et al., 2007; Lin et al., 2008a; Simpson et al., 2008; Kusaka et al., 2014; Wang et al., 2015b; Freitag et al., 2018; Fan et al., 2020), comprehensive quantification of URE is challenging. Since the beginning, the research focus has been on verifying whether there is a general precipitation enhancement downwind of urban areas. Based on various observations and model simulations, many studies found an enhancement in precipitation, precipitation intensity, or extreme precipitation in urban downwind areas, on the periphery of the urban core, or over the urban areas (Huff and Changnon, 1973; Shepherd et al., 2002; Burian and Shepherd, 2005; Chen et al., 2007; Mote et al., 2007; Van Den Heever and Cotton, 2007; Simpson et al., 2008; Carrió et al., 2010; Schlünzen et al., 2010; Shepherd et al., 2010; Yang et al., 2012, 2014; Kusaka et al., 2014; Dou et al., 2015; Haberlie et al., 2015; Yu and Liu, 2015; Daniels et al., 2016; Ryu et al., 2016b; Liang and Ding, 2017; McLeod et al., 2017; Niyogi et al., 2017; Schmid and Niyogi, 2017; Sarangi et al., 2018; Wu et al., 2019; Fan et al., 2020; Kremer, 2020; Li et al., 2020b; Marelle et al., 2020; Singh et al., 2020; Lin et al., 2021).

Meanwhile many other studies argued that the enhancement in urban or downwind precipitation or precipitation intensity was insignificant, and some studies even showed a reduction in precipitation over or downwind of urban areas, possibly due to evaporation inhibition and moisture deficit (Block et al., 2004; Guo et al., 2006; Kaufmann et al., 2007; Zhang et al., 2009; Miao et al., 2011; Wang et al., 2012; Ganeshan and Murtugudde, 2015; Yang et al., 2016; Wang et al., 2018). Moreover, Li et al. (2011) found that the Pearl River

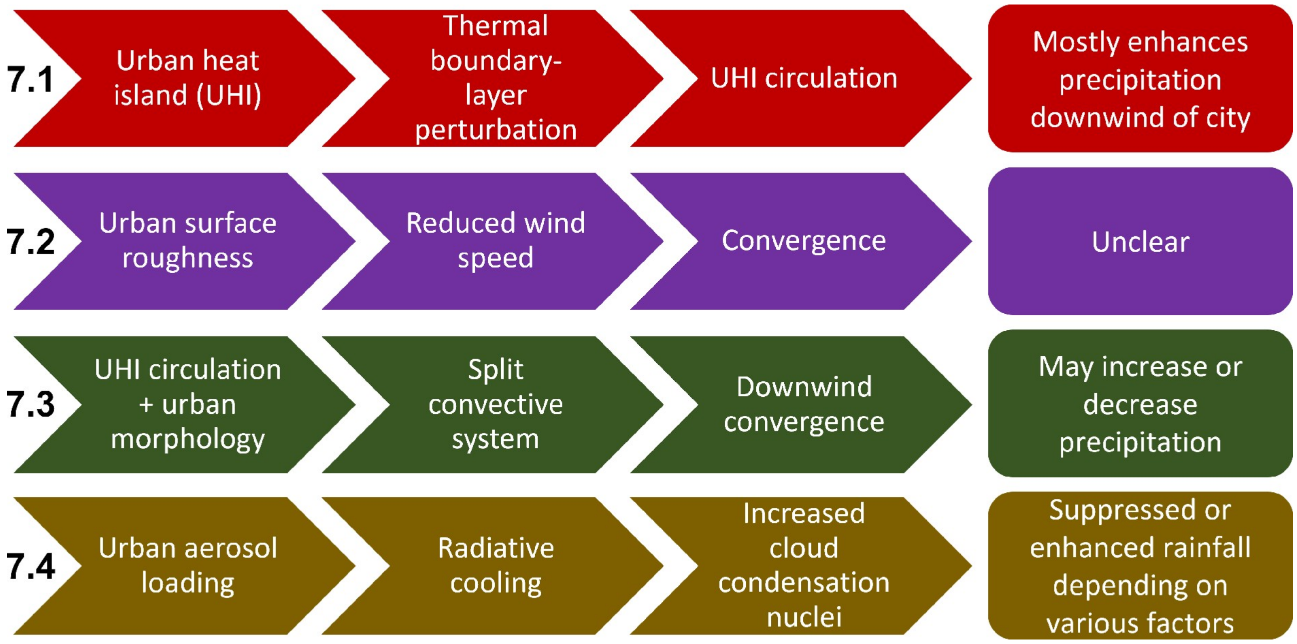

**Fig. 8.** Conceptual understanding of urban rain effect (URE).

Delta metropolitan regions of China experienced more strong precipitation but less weak precipitation than surrounding non-urban areas. There is no doubt that spatiotemporal variations in climatology, meteorology, topography, the degree of urbanization, and aerosol emissions can cause different URE in different studies. However, the different methods used in previous studies can also lead to discrepancies.

Three approaches are widely used to examine URE. a) The first is based on the comparison of two numerical simulations with and without urbanization (Chen et al., 2016; Fan et al., 2020; Lin et al., 2021); b) The second is through the comparison of precipitation over urban areas and surrounding non-urban areas (Mote et al., 2007; Haberlie et al., 2015; Daniels et al., 2016); c) The third is to compare precipitation during pre-urbanization periods and post-urbanization periods (Huff and Changnon, 1973; Burian and Shepherd, 2005; Niyogi et al., 2017). Comparison between weekdays and weekends is also used in some studies to examine the potential impact of human activity on urban precipitation (Jin et al., 2005; Gong et al., 2007; Haberlie et al., 2015). Strictly speaking, these methods reflect different aspects of URE. Lowry (1977) empirically discussed the limitations of these methods and recommended further substantial examinations of URE for different synoptic weather systems, periods, and urban regions, highlighting the importance of the aforementioned studies to investigate URE around the globe. Although previous studies have shown inconsistent results, a meta-analysis conducted by Liu and Niyogi (2019) demonstrated a robust conclusion about the existence of URE. Following the suggestion of Lowry (1977), more studies are necessary to thoroughly understand the impact of urbanization on precipitation.

## 8. Summary and future direction

### 8.1. *Summary of current understanding*

Below we provide a summary of our current understanding of different aspects of the impact of urbanization on regional climate and extreme weather based on observations and modeling (Fig. 9).

#### 8.1.1. *Impact on temperature and heat waves*

Observations across cities show that urban areas are generally warmer than their surroundings, particularly during summer nights. The magnitude of this thermal anomaly depends on the biophysical and morphological properties of the city, including fraction of urban green space, the reflectivity of urban surfaces, and the vertical structure of the city, but also on the land use/land cover (LULC) of the reference rural area. Notably, the magnitude of the UHI is quite different depending on how it is measured, using in situ sensors (CUHI) or satellite observations (SUHI). The diurnality of the SUHI and CUHI also seems to flip in most cities, with the CUHI higher at night and the SUHI higher during the day. Satellite observations, unlike observations from ground-based sensors, can detect the intra-urban variability of the thermal environment. The long-term trends in the climatological mean SUHI and CUHI have not increased substantially over the last few decades, except for in regions of Asia and Africa, with increasing urbanization. However, the trend in extreme heat events in cities is more uncertain due to the possibility of compound events (Argüeso et al., 2014; Founda and Santamouris, 2017).

#### 8.1.2. *Impact on PBL, clouds, and air pollution*

The UHI effect tends to induce self-contained circulations (urban dome) centered around urban areas when the ambient wind is weak. On the other hand, the flow pattern resembles a plume (urban plume) when the ambient wind is strong. When cities are located in coastal or mountainous regions, the wind pattern is more complex. Not only does the urban land affect the mean wind field, turbulence and turbulent transport in the boundary layer are also strongly modulated by the enhanced roughness and stronger buoyancy flux over urban areas, yielding a faster-growing and higher boundary layer under daytime conditions. Recent studies also suggest that the stronger turbulent transport is responsible for the extended periods of low-level clouds over cities. The UHI effect favors the dispersion of pollutants from urbanized areas to their immediate vicinities by destabilizing the lower atmosphere and increasing ventilation over the urban area.

#### 8.1.3. *Precipitation and storms*

Substantial evidence shows that urbanization can affect precipitation; urban areas are generally drier than their surroundings, with the exception of cities in arid environments. However, how urbanization affects precipitation is still under debate due to the involvement of various dynamical and thermodynamic processes. Four major mechanisms are proposed to explain the observed or simulated URE for cities around the globe: 1) UHI–thermal perturbation of the boundary layer; 2) enhanced convergence due to increased urban surface roughness; 3) modification of propagating precipitating systems by the urban landscape; 4) urban aerosol effect on local precipitation. These mechanisms generally occur simultaneously and interact with many other environmental factors, impeding a comprehensive qualification and quantification of URE and urging further studies to improve our understanding of URE.

### 8.2. *Uncertainties and challenges*

Substantial scientific advances have been made in understanding and modeling the impacts of urbanization on regional and global weather and climate in the past few decades. However, important uncertainties and challenges still exist in the current knowledge, observations, and modeling capabilities. Our current understanding of urban physical, hydrological, and biogeochemical processes and associated interactions with other Earth system components is still limited. Most previous studies have focused on urban thermodynamic processes and impacts on the mean state and trend of temperature and precipitation, whereas few efforts have been put into other important aspects like urban hydrology (e.g., groundwater, flooding, drought, water quality), urban biogeochemical cycle, spatial heterogeneity in urban morphology, impact of detailed urban energy and building structures, complex feedbacks to regional and large-scale hydroclimate, urban vegetation, urban heat stress, and urban mitigation and adaption to climate change. It is worth noting that some recent studies have begun to look at urbanization and its relationship with extreme precipitation and heat waves, which is a good start for improving our understanding of the mechanisms involved. The uncertainties and insufficiency in observations and modeling capabilities are important contributors to the limitations in the current knowledge.

#### 8.2.1. *Limitations in measurements*

The types of datasets used to study the urban climate have their own weaknesses. For example, land cover data convey little information on surface geometry. The use of population metadata may lead to biased results because they may not be representative of the immediate vicinity of the stations. In addition, cultural and regional discrepancies in urban–rural threshold values for population and nighttime lights further undermine their appropriateness for station classification (Stewart, 2011a). Although more and more measurements over urban areas have become available, measurements focusing on specific urban processes are still scarce or without enough spatiotemporal details.

There are numerous regularly maintained meteorological stations in urban areas around the world, but they have some key limitations.

(1) Most observations are traditional, near-surface measurements without vertical profiles across the urban column. Vertical profiles are particularly useful in areas of tall buildings or significantly varying building heights. Some recent efforts (e.g., Nadeau et al., 2009) expand the ability of atmospheric profiling.

(2) Few urban stations include observations with long-term records (e.g., precipitation and pollutants), limiting the investigations of the long-term urbanization impact on weather/climate, pollution, and extreme events.

(3) Many urban stations are sited for easy access but not necessarily to capture the spatial heterogeneity of the entire urban region. For instance, Peterson (2003) found no statistically significant difference between temperature measurements from urban and rural weather stations in the contiguous US at the annual scale, which he postulated was because urban stations were more likely in urban parks, not over warmer industrial regions.

(4) Traditional urban measurements only include a few common meteorological variables (e.g., temperature, precipitation, and humidity) often without a full suite of other complementary observations (e.g., air pollutants and urban-boundary layer exchange flux), which makes a better understanding of urban-climate/weather/pollution interactions and feedbacks particularly challenging.

(5) Recently, some new urban observations (e.g., lidar and aircraft campaign) are becoming available, but they are still limited in terms of spatiotemporal coverage. Compared with ground-based measurements, satellite observations are especially useful due to the large spatial coverage, but often they do not have sufficient high spatial resolution or long-term records for urban studies. The challenges and uncertainties in observations also partially limit the improvement of urban modeling capabilities.

(6) Time-space completeness: While in situ, stationary measurements inform us on temporal variability, mobile observations and polar orbiting satellites contribute spatial information from local to regional scales. Geostationary satellites and crowd sourcing approaches provide optimal observations at both spatial and temporal scales. However, geostationary satellites can provide observations of SUHI only, while crowd sourcing approaches provide observations of CUHI only.

(7) When dealing with in situ observations, another major limitation has been consistency in the choice of urban and rural weather stations and their placements when trying to quantify the CUHI (Stewart, 2011a). Stewart (2011b) provided a systematic critique of the literature and suggested that approximately half of the reported CUHI magnitudes lacked robustness. Many studies do not control for weather factors and do not provide sufficient metadata about the instrumentation and field characteristics during the study period, thus indicating poor scientific practice. In the same vein, Peterson and Owen (2005) emphasized the importance of the metadata used in UHI studies. Stewart and Oke (2012) created the LCZ classification framework, which was designed to standardize the description of observation sites and thereby ease the process of station selection in UHI studies.

(8) The lack of metadata for in situ stations is even more apparent for crowd sourced data (Muller et al., 2015). Many private weather stations do not include radiation shields in the default sensor configuration (Venter et al., 2021). All else remaining equal, the lack of a radiation shield would cause sensors to overestimate air temperature and underestimate relative humidity (Da Cunha, 2015). Although quality control procedures have been developed for some of these crowd sourced datasets, particularly for air temperature, they generally rely on statistical tests and cannot account for systematic biases due to the lack of radiation shields (Napoly et al., 2018). Moreover, these statistical tests are meant to improve estimates of overall air temperature, not the urban–rural differential in air temperature (or CUHI), which is a much smaller signal.

(9) Since mobile measurements do not necessarily take simultaneous measurements at multiple points, the temporal variation of temperature needs to be accounted for when using them to estimate the spatial patterns of CUHI (Stewart, 2011a). Moreover, measurements using sensors mounted on vehicles are restricted to ambient temperature over roads. This issue is mitigated somewhat when using meteorological sensors in smart devices, which can also be active over urban non-road surfaces. However, uncertainties

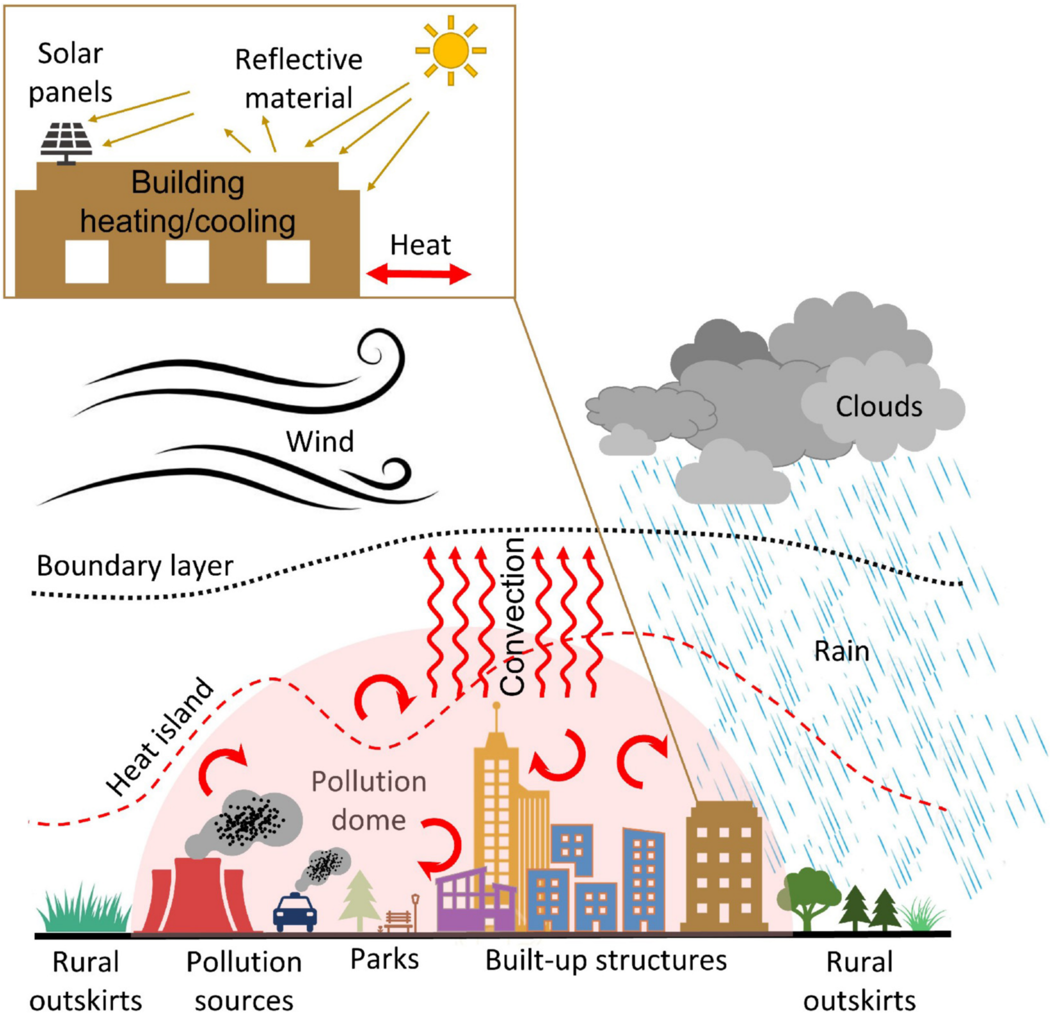

**Fig. 9.** Schematic picture of urban impact on weather and climate.

arise from contamination by radiated body heat, though this may be frequently intended (Nazarian et al., 2021).

(10) Similarly, there are several uncertainties in remote estimates of the SUHI, and multiple methods have been proposed to estimate the SUHI intensity at various scales (Rozenfeld et al., 2008; Peng et al., 2012; Li et al., 2018a; Chakraborty and Lee, 2019; Zhou et al., 2019; Chakraborty et al., 2021b). How to define the rural background temperature has been a point of contention in the literature (Martin-Vide et al., 2015; Zhang et al., 2019a). In many cases, the rural background is defined using a constant buffer around the urban boundary (Clinton and Gong, 2013). More recently, Chakraborty et al. (2021a) defined the rural reference using a normalized buffer weighted by the area of the urban cluster. However, studies have shown that there is a footprint of the SUHI that can extend well into these buffers (Zhou et al., 2015a; Yang et al., 2019b). This is relevant because, due to its traditional definition, the SUHI intensity is modulated by changes in both urban and rural land cover (Chakraborty et al., 2017; Kumar et al., 2017; Martilli et al., 2020; Chakraborty et al., 2021a). Among many others, Chakraborty and Lee (2019) have argued for the use of physical urban clusters when defining the SUHI instead of the administrative boundaries of cities. However, this solution is not trivial to implement. Even with the availability of several global urban extent datasets (Zhou et al., 2015b; Esch et al., 2017; Melchiorri et al., 2018; Li et al., 2020a), an order of magnitude difference has been seen between them (Potere and Schneider, 2007).

(11) The methods of temporal compositing and LST-retrieval algorithm used to derive the SUHI have also both been shown to influence the estimates (Hu and Brunsell, 2013; Yao et al., 2020; Chakraborty et al., 2020). Benz et al. (2021) recently derived global anomalies in LST due to urbanization on continuous surfaces rather than by using predefined urban geometries, though this definition makes it harder to compare the LST of adjacent pixels. On a more fundamental note, LST-retrieval methods are constrained by assumptions of surface emissivity ($\varepsilon$) since LST and $\varepsilon$ cannot be simultaneously solved for using analytical methods (Dash et al., 2002). Methods used to prescribe $\varepsilon$ are frequently used over urban areas even though the original empirical methods were intended for natural surfaces (Chakraborty et al., 2021b). Estimating the bulk $\varepsilon$ over cities is confounded by several factors, from the variability in building material to the presence of urban vegetation to surface morphology (Voogt and Oke, 1998). Thermal anisotropy due to the vertical structure of urban areas also contributes to large uncertainties in satellite-observed LST and SUHI (Hu et al., 2016). As a general note, satellite-derived LST and SUHI are only valid for clear-sky conditions.

8.2.2. *Uncertainties in modeling*

Many urban parameterizations and their coupling with weather/climate models have been developed for various applications in the past decades, and they are generally able to capture the major surface–atmosphere exchange processes in urban areas. However, important urban modeling challenges remain.

(1) Most existing urban models only represent the thermodynamic and dynamical effects of buildings on the turbulent fluxes and mean flows, whereas few include the explicit treatment of urban vegetation and its interactions with buildings and street canyons (Lee et al., 2016). The lack of urban vegetation, which plays an important role in modulating urban radiation, heat fluxes, and the hydrological cycle, could lead to uncertainties in urban climate/weather modeling.

(2) Uncertainties also exist in various urban model parameters (Chen et al., 2021), including those related to anthropogenic heat (e.g., air conditioning; De Munck et al., 2013; Xu et al., 2018), landscape irrigation, building wall function (determining indoor–outdoor heat exchange), and urban spatial heterogeneity (e.g., building/vegetation horizontal and vertical distribution). Recent efforts in developing high-resolution urban-model parameters such as LCZ (Stewart et al., 2014) and WUDAPT (Ching et al., 2019) are an excellent step forward. However, many of the critical urban morphological parameters are not well represented for developing cities in these models (Sun et al., 2021), which is an important area for future research.

(3) Many important urban processes and associated mutual interactions are still missing in most models, such as urban hydrology and inundation, biogeochemistry, and interactions between pollution and hydroclimate, which are key to improved water resource management and public health maintenance. An integrated urban system model will include many processes associated with natural earth system and human activities and their interactions (Fig. 10).

(4) As urban modeling is applied to multiple scales in different applications, the scale-dependence nature of existing urban parameterizations has yet to be explored.

(5) The coupling of urban models with mesoscale or global weather/climate models poses another challenge, where efficient techniques are needed to optimize the integration of detailed 3D urban-surface heterogeneity and large-scale boundary layer at coarse resolution. The coupling between urban atmosphere and its surrounding rural counterpart is also critical, and their uncertainties are often entangled with each other. The compatibility of urban physics with other model physics (particularly surface and boundary layer physics) during coupling could also introduce uncertainty.

(6) Comprehensive urban model evaluation remains a

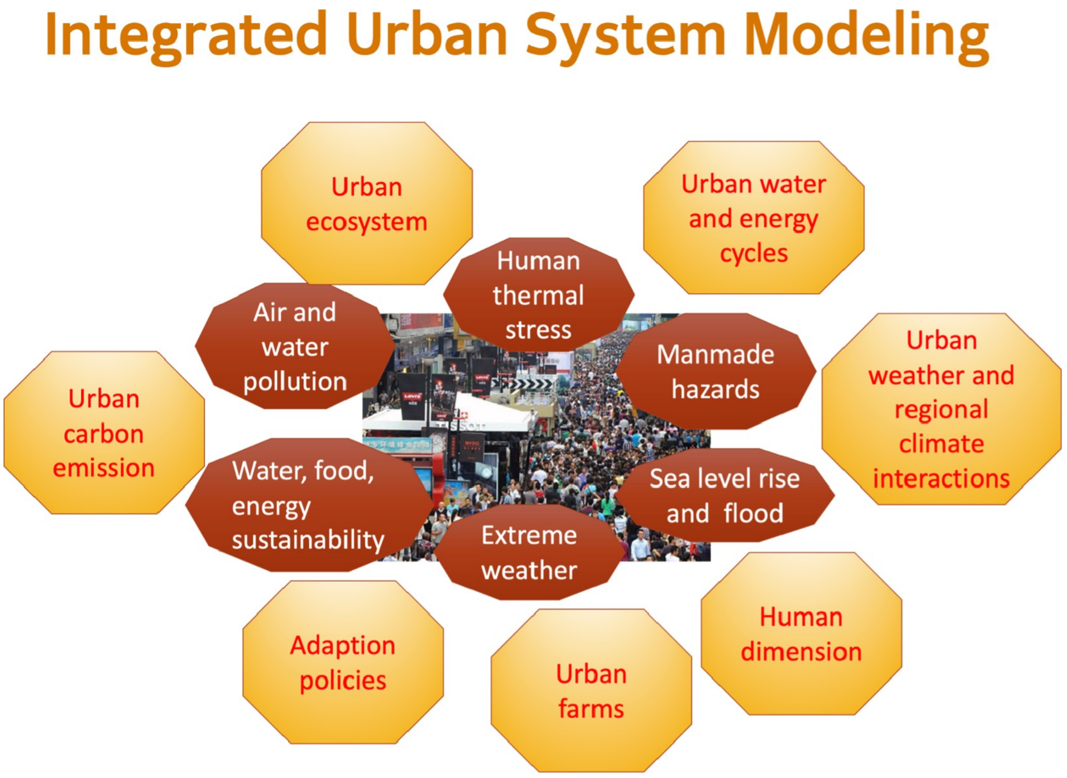

**Fig. 10.** A schematic figure of an integrated urban system model.

challenge, partially due to the lack of necessary measurements. Existing urban model assessments are limited in specific areas and applications, which leave questions about the applicability of these models.

#### 8.2.3. *Model–observation integration*

Urban observational and modeling communities have traditionally lacked sufficient communications and collaborations, which prevents an improved understanding in this field. Discussions between scientists focusing on measurements and modeling can offer mutual benefits. For example, modeling studies can provide information for urban hotspots and potentially best places to employ measurements, while measurements can guide the development and improvement of urban model parameterizations. Better observation–model integration is needed to make progress in urban studies.

#### 8.2.4. *Researchers–stakeholder communication and crowd sourcing campaigns/public participation*

Finally, a lack of connections between end users and researchers also slows down the progress in urban studies. End users and stakeholders often can provide important information regarding the urgent direction and application of urban investigations, while the scientific community offers guidance for users to better tackle urban impacts on public health and adaptation to extreme weather. Without a mutual understanding from both groups, the effectiveness of urban studies will be significantly reduced. Currently, finding an effective platform or framework for a close collaboration between end users and the scientific community remains a challenge, but efforts are being made along this line. In the future, co-production of knowledge that takes into account the perspectives of the end users would be relevant to effective design of urban heat mitigation strategies

### 8.3. *Recommendation of future research priorities and directions*

Some thoughts about future urban research priorities were identified in Chen et al. (2012) and Sharma et al. (2020). Hu et al. (2020) used big data analytics to identify emerging fields in urban climatology. Here, we provide a short summary of these findings and our perspectives regarding future research directions and priorities.

(1) Establish a coordinated network of long-term comprehensive urban observations

Improving knowledge of different urban processes, particularly their interactions and feedbacks, requires urban measurements to have relatively long-term (e.g., multiple years that allow a climatological signal to emerge from background noise) records and be well characterized in terms of meteorology, air pollution, and biogeochemical cycles. Such long-term comprehensive urban measurements can be built on existing urban observations and networks. Areas that are associated with strong urban impact and interactions with weather, climate, and pollution (i.e., urban hotspots), or urban regions that are historically under-studied should be prioritized for future measurements. It is also important to leverage aircraft campaigns and satellite observations along with ground-based measurements, which can complement site measurements by offering global and large-scale constraints for urban processes.

(2) Synthesize, quality control, and publicize existing urban datasets

Advancing process-based urban knowledge requires accessible standardized data across different sites and quantification of data uncertainty. Although a substantial volume of urban data has been generated by previous observational studies, the data often go through different data quality control procedures, are scattered across different platforms, or are not accessible online. Future efforts are needed to collect and synthesize existing urban datasets, conduct consistent data quality control and uncertainty quantification procedures following a standard data protocol (e.g., data formatting, filtering, and correction), and make the datasets publicly available with sufficient metadata. Crowdsourced measurements, both from private weather stations and smart devices, can contribute to better monitoring of urban areas, though there is much work to be done to establish quality control procedures for these emerging data sources.

(3) Reduce uncertainties in cross-scale modeling of key urban processes and coupling with climate models

Several key urban processes and components, like urban hydrology and vegetation, and their interactions and feedbacks to urban weather and climate are still missing or simplistic in current models. Improving the description of urban landscapes and urban model parameters are important for mitigating urban modeling uncertainties. Developing or improving scale-aware urban parameterizations will also be helpful to more accurately predict urban impacts across scales. In the last few years, several attempts at incorporating vegetation and hydrology in urban canopy models have shown promising results (Lee and Park, 2008; Yang et al., 2015; Ryu et al., 2016a; Liu et al., 2017; Meili et al., 2020; Mussetti et al., 2020; Krayenhoff et al., 2020; Redon et al., 2020; Li and Liu, 2021). Since model selection depends heavily on science and application purposes, the continued development of simple urban models that fit specific engineering and urban-planning applications should be encouraged.

(4) Evaluate coupled atmosphere–urban modeling systems for extreme conditions

Most urban models coupled to weather and climate models were developed and evaluated with measurements of climatological conditions and may not work for extreme conditions. Therefore, a systematic assessment of these coupled models in extreme conditions (extreme heat, hurricane, extreme precipitation, etc.) is necessary. When trying to isolate the urban signal, it is also important to specifically evaluate the magnitude of the signal (i.e., the difference in the variable of interest in the urban area and that in its background climate) instead of evaluating the overall variables (as in, evaluating the UHI versus the urban temperature).

(5) Better integration of urban observations and modeling

Future efforts on better combining urban measurements and models to facilitate process-level understanding of urban physics, chemistry, and impacts on other earth system components are needed. For example, current urban models are associated with nontrivial uncertainties. How to leverage suitable measurements to pinpoint urban model deficiencies and reduce uncertainties in related urban parameters and physics will be extremely helpful. The modeling groups also need to inform observational groups with specific requirements to facilitate the model–observation synthesis. Overall, close collaborations between urban modeling and measurement groups are strongly encouraged, which will help advance the knowledge transfer between observations and urban modeling. Satellite observations can be particularly useful in this regard since they provide spatially continuous estimates of the radiative and thermodynamic properties of the urban surface, which are currently poorly constrained in models.

(6) Application of machine learning (ML) and data assimilation techniques

Future studies can adopt machine learning (ML) as an efficient tool for studying and understanding the complexities of the urban environment. With the data currently available from satellites, existing weather stations, and crowdsourced measurements, these methods can be leveraged to emulate the spatial variability of the urban environment, detect relevant morphological features within cities, isolate the urban contribution to local meteorological variables, and classify urban LULC into further subcategories. Moreover, ML can be combined with existing physics-based models to both downscale model results at coarse resolutions and develop new data-driven parameterizations to capture currently unresolved urban interactions. As more and more urban observations become available, urban data assimilation is also a promising way to constrain estimates of urban pollution, weather, and climate. Only a handful of studies have focused on urban-specific data assimilation, which should be explored more in the future. Given the large uncertainties in urban-scale observations due to station inhomogeneities, sampling biases, and urban surface anisotropy, ML techniques can be indispensable tools for bias correcting, gap filling, and quality controlling these observations.

(7) Facilitate connections and collaborations between scientists and end users

Meaningful collaborations between scientists and end users (e.g., stakeholders and policymakers) can inspire new research needs and directions and lead to evidence-based decision making related to urban problems, including public heath, climate adaptation, mitigation to extreme weather, and urban water and energy resource management. Mutual understanding between scientists and end users is also beneficial for developing or improving urban application tools and ultimately fostering actionable urban science. Thus, platforms and opportunities that promote connections between scientists and end users are needed.

***Acknowledgements***. This research has been supported by the US Department of Energy, Office of Science, Biological and Environmental Research program, as part of the Regional and Global Modeling and Analysis (RGMA) program, Multi-sector Dynamics Modeling (MSD) program, and Earth System Model Development (ESMD) program, through the collaborative, multiprogram Integrated Coastal Modeling (ICoM) project, HyperFACETS project, and COMPASS-GLM project. Pacific Northwest National Laboratory is operated for the Department of Energy by Battelle Memorial Institute under contract DE-AC05-76RL01830.

***Open Access*** This article is licensed under a Creative Commons Attribution 4.0 International License, which permits use, sharing, adaptation, distribution and reproduction in any medium or format, as long as you give appropriate credit to the original author(s) and the source, provide a link to the Creative Commons licence, and indicate if changes were made. The images or other third party material in this article are included in the article's Creative Commons licence, unless indicated otherwise in a credit line to the material. If material is not included in the article's Creative Commons licence and your intended use is not permitted by statutory regulation or exceeds the permitted use, you will need to obtain permission directly from the copyright holder. To view a copy of this licence, visit http://creativecommons.org/licenses/by/4.0/.

1520-0442(1999)012<1344:TTOTUS>2.0.CO;2.

Ganeshan, M., and R. Murtugudde, 2015: Nocturnal propagating thunderstorms may favor urban "hot-spots": A model-based study over Minneapolis. *Urban Climate*, **14**, 606–621, https://doi.org/10.1016/j.uclim.2015.10.005.

Garratt, J. R., 1995: Observed screen (air) and GCM surface/screen temperatures: Implications for outgoing longwave fluxes at the surface. *J. Climate*, **8**, 1360–1368, https://doi.org/10.1175/1520-0442(1995)008<1360:OSAGST>2.0.CO;2.

Garuma, G. F., 2018: Review of urban surface parameterizations for numerical climate models. *Urban Climate*, **24**, 830–851, https://doi.org/10.1016/j.uclim.2017.10.006.

Georgescu, M., A. M. Broadbent, M. Wang, E. S. Krayenhoff, and M. Moustaoui, 2021: Precipitation response to climate change and urban development over the continental United States. *Environmental Research Letters*, **16**, 044001, https://doi.org/10.1088/1748-9326/abd8ac.

Giannaros, T. M., D. Melas, I. A. Daglis, I. Keramitsoglou, and K. Kourtidis, 2013: Numerical study of the urban heat island over Athens (Greece) with the WRF model. *Atmos. Environ.*, **73**, 103–111, https://doi.org/10.1016/j.atmosenv.2013.02.055.

Giometto, M. G., A. Christen, P. E. Egli, M. F. Schmid, R. T. Tooke, N. C. Coops, and M. B. Parlange, 2017: Effects of trees on mean wind, turbulence and momentum exchange within and above a real urban environment. *Advances in Water Resources*, **106**, 154–168, https://doi.org/10.1016/j.advwatres.2017.06.018.

Giovannini, L., D. Zardi, M. de Franceschi, and F. Chen, 2014: Numerical simulations of boundary-layer processes and urban-induced alterations in an Alpine valley. *International Journal of Climatology*, **34**, 1111–1131, https://doi.org/10.1002/joc.3750.

Givati, A., and D. Rosenfeld, 2004: Quantifying precipitation suppression due to air pollution. *J. Appl. Meteorol. Climatol.*, **43**, 1038–1056, https://doi.org/10.1175/1520-0450(2004)043<1038:QPSDTA>2.0.CO;2.

Godowitch, J. M., J. K. S. Ching, and J. F. Clarke, 1985: Evolution of the nocturnal inversion layer at an urban and nonurban location. *J. Appl. Meteorol. Climatol.*, **24**, 791–804, https://doi.org/10.1175/1520-0450(1985)024<0791:EOTNIL>2.0.CO;2.

Golroudbary, V. R., Y. J. Zeng, C. M. Mannaerts, and Z. B. Su, 2017: Detecting the effect of urban land use on extreme precipitation in the Netherlands. *Weather and Climate Extremes*, **17**, 36–46, https://doi.org/10.1016/j.wace.2017.07.003.

Gong, D.-Y., C. H.-Ho, D. L. Chen, Y. Qian, Y.-S. Choi, and J. Kim, 2007: Weekly cycle of aerosol-meteorology interaction over China. *J. Geophys. Res.*, **112**, D22202, https://doi.org/10.1029/2007JD008888.

Gough, W. A., 2020: Thermal signatures of peri-urban landscapes. *J. Appl. Meteorol. Climatol.*, **59**, 1443–1452, https://doi.org/10.1175/JAMC-D-19-0292.1.

Grimmond, C. S. B., and T. R. Oke, 2002: Turbulent heat fluxes in urban areas: Observations and a local-scale urban meteorological parameterization scheme (LUMPS). *J. Appl. Meteorol. Climatol.*, **41**, 792–810, https://doi.org/10.1175/1520-0450(2002)041<0792:THFIUA>2.0.CO;2.

Grimmond, C. S. B., H. A. Cleugh, and T. R. Oke, 1991: An objective urban heat storage model and its comparison with other schemes. *Atmospheric Environment. Part B. Urban Atmosphere*, **25**, 311–326, https://doi.org/10.1016/0957-1272(91)90003-W.

Grimmond, C. S. B., and Coauthors, 2011: Initial results from Phase 2 of the international urban energy balance model comparison. *International Journal of Climatology*, **31**, 244–272, https://doi.org/10.1002/joc.2227.

Güneralp, B., M. Reba, B. U. Hales, E. A. Wentz, and K. C. Seto, 2020: Trends in urban land expansion, density, and land transitions from 1970 to 2010: A global synthesis. *Environmental Research Letters*, **15**, 044015, https://doi.org/10.1088/1748-9326/ab6669.

Guo, G. H., Z. F. Wu, R. B. Xiao, Y. B. Chen, X. N. Liu, and X. S. Zhang, 2015: Impacts of urban biophysical composition on land surface temperature in urban heat island clusters. *Landscape and Urban Planning*, **135**, 1–10, https://doi.org/10.1016/j.landurbplan.2014.11.007.

Guo, X. L., D. H. Fu, and J. Wang, 2006: Mesoscale convective precipitation system modified by urbanization in Beijing City. *Atmospheric Research*, **82**, 112–126, https://doi.org/10.1016/j.atmosres.2005.12.007.

Gutiérrez, E., A. Martilli, J. L. Santiago, and J. E. González, 2015: A mechanical drag coefficient formulation and urban canopy parameter assimilation technique for complex urban environments. *Bound.-Layer Meteorol.*, **157**, 333–341, https://doi.org/10.1007/s10546-015-0051-7.

Haberlie, A. M., W. S. Ashley, and T. J. Pingel, 2015: The effect of urbanisation on the climatology of thunderstorm initiation. *Quart. J. Roy. Meteor. Soc.*, **141**, 663–675, https://doi.org/10.1002/qj.2499.

Hajmohammadi, H., and B. Heydecker, 2021: Multivariate time series modelling for urban air quality. *Urban Climate*, **37**, 100834, https://doi.org/10.1016/j.uclim.2021.100834.

Halfon, N., Z. Levin, and P. Alpert, 2009: Temporal rainfall fluctuations in Israel and their possible link to urban and air pollution effects. *Environmental Research Letters*, **4**, 025001, https://doi.org/10.1088/1748-9326/4/2/025001.

Hamdi, R., 2010: Estimating urban heat island effects on the temperature series of Uccle (Brussels, Belgium) using remote sensing data and a land surface scheme. *Remote Sensing*, **2**, 2773–2784, https://doi.org/10.3390/rs2122773.

Han, J.-Y., and J.-J. Baik, 2008: A theoretical and numerical study of urban heat island–induced circulation and convection. *J. Atmos. Sci.*, **65**, 1859–1877, https://doi.org/10.1175/2007JAS2326.1.

Han, J.-Y., J.-J. Baik, and A. P. Khain, 2012: A numerical study of urban aerosol impacts on clouds and precipitation. *J. Atmos. Sci.*, **69**, 504–520, https://doi.org/10.1175/JAS-D-11-071.1.

Han, J.-Y., J.-J. Baik, and H. Lee, 2014a: Urban impacts on precipitation. *Asia-Pacific Journal of Atmospheric Sciences*, **50**, 17–30, https://doi.org/10.1007/s13143-014-0016-7.

Han, W. C., Z. Q. Li, J. P. Guo, T. N. Su, T. M. Chen, J. Wei, and M. Cribb, 2020: The urban–rural heterogeneity of air pollution in 35 metropolitan regions across China. *Remote Sensing*, **12**(14), 2320, https://doi.org/10.3390/rs12142320.

Han, Z. Q., Z. W. Yan, Z. Li, W. D. Liu, and Y. C. Wang, 2014b: Impact of urbanization on low-temperature precipitation in Beijing during 1960–2008. *Adv. Atmos. Sci.*, **31**, 48–56, https://doi.org/10.1007/s00376-013-2211-3.

Hand, L. M., and J. M. Shepherd, 2009: An investigation of warm-season spatial rainfall variability in Oklahoma City: Possible linkages to urbanization and prevailing wind. *J. Appl.*

*Meteorol. Climatol.*, **48**, 251–269, https://doi.org/10.1175/2008JAMC2036.1.

Hansen, J., R. Ruedy, M. Sato, M. Imhoff, W. Lawrence, D. Easterling, T. Peterson, and T. Karl, 2001: A closer look at United States and global surface temperature change. *J. Geophys. Res.*, **106**, 23 947–23 963, https://doi.org/10.1029/2001JD000354.

Harlan, S. L., A. J. Brazel, L. Prashad, W. L. Stefanov, and L. Larsen, 2006: Neighborhood microclimates and vulnerability to heat stress. *Social Science & Medicine*, **63**, 2847–2863, https://doi.org/10.1016/j.socscimed.2006.07.030.

Hass, A. L., K. N. Ellis, L. Reyes Mason, J. M. Hathaway, and D. A. Howe, 2016: Heat and humidity in the city: Neighborhood heat index variability in a mid-sized city in the southeastern United States. *International Journal of Environmental Research and Public Health*, **13**, 117, https://doi.org/10.3390/ijerph13010117.

Hausfather, Z., M. J. Menne, C. N. Williams, T. Masters, R. Broberg, and D. Jones, 2013: Quantifying the effect of urbanization on U. *S. Historical Climatology Network temperature records. J. Geophys. Res.*, **118**, 481–494, https://doi.org/10.1029/2012JD018509.

He, C. Y., Z. F. Liu, J. G. Wu, X. H. Pan, Z. H. Fang, J. W. Li, and B. A. Bryan, 2021: Future global urban water scarcity and potential solutions. *Nature Communications*, **12**, 4667, https://doi.org/10.1038/s41467-021-25026-3.

He, X. D., J. Wang, J. M. Feng, Z. W. Yan, S. G. Miao, Y. Z. Zhang, and J. J. Xia, 2020: Observational and modeling study of interactions between urban heat island and heatwave in Beijing. *Journal of Cleaner Production*, **247**, 119169, https://doi.org/10.1016/j.jclepro.2019.119169.

Hertwig, D., G. C. Efthimiou, J. G. Bartzis, and B. Leitl, 2012: CFD-RANS model validation of turbulent flow in a semi-idealized urban canopy. *Journal of Wind Engineering and Industrial Aerodynamics*, **111**, 61–72, https://doi.org/10.1016/j.jweia.2012.09.003.

Hildebrand, P. H., and B. Ackerman, 1984: Urban effects on the convective boundary layer. *J. Atmos. Sci.*, **41**, 76–91, https://doi.org/10.1175/1520-0469(1984)041<0076:UEOTCB>2.0.CO;2.

Ho, H. C., A. Knudby, P. Sirovyak, Y. M. Xu, M. Hodul, and S. B. Henderson, 2014: Mapping maximum urban air temperature on hot summer days. *Remote Sensing of Environment*, **154**, 38–45, https://doi.org/10.1016/j.rse.2014.08.012.

Ho, H. C., A. Knudby, Y. M. Xu, M. Hodul, and M. Aminipouri, 2016: A comparison of urban heat islands mapped using skin temperature, air temperature, and apparent temperature (Humidex), for the greater Vancouver area. *Science of the Total Environment*, **544**, 929–938, https://doi.org/10.1016/j.scitotenv.2015.12.021.

Hoffman, J. S., V. Shandas, and N. Pendleton, 2020: The effects of historical housing policies on resident exposure to intra-urban heat: A study of 108 US urban areas. *Climate*, **8**, 12, https://doi.org/10.3390/cli8010012.

Hoffmann, P., O. Krueger, and K. H. Schlünzen, 2012: A statistical model for the urban heat island and its application to a climate change scenario. *International Journal of Climatology*, **32**, 1238–1248, https://doi.org/10.1002/joc.2348.

Holmer, B., and I. Eliasson, 1999: Urban–rural vapour pressure differences and their role in the development of urban heat islands. *International Journal of Climatology*, **19**, 989–1009, https://doi.org/10.1002/(SICI)1097-0088(199907)19:9<989::AID-JOC410>3.0.CO;2-1.

Holt, T., and J. Pullen, 2007: Urban canopy modeling of the New York City metropolitan area: A comparison and validation of single- and multilayer parameterizations. *Mon. Wea. Rev.*, **135**, 1906–1930, https://doi.org/10.1175/MWR3372.1.

Horton, R. E., 1921: Thunderstorm-breeding spots. *Mon. Wea. Rev.*, **49**, 193, https://doi.org/10.1175/1520-0493(1921)49<193a:TS>2.0.CO;2.

Hou, A. Z., G. H. Ni, H. B. Yang, and Z. D. Lei, 2013: Numerical analysis on the contribution of urbanization to wind stilling: An example over the Greater Beijing Metropolitan Area. *J. Appl. Meteorol. Climatol.*, **52**, 1105–1115, https://doi.org/10.1175/JAMC-D-12-013.1.

Howard, L., 1833: *The Climate of London: Deduced from Meteorological Observations Made in the Metropolis and at Various Places around It*. Cambridge University Press.

Hsu, A., G. Sheriff, T. Chakraborty, and D. Manya, 2021: Disproportionate exposure to urban heat island intensity across major US cities. *Nature Communications*, **12**, 2721, https://doi.org/10.1038/s41467-021-22799-5.

Hu, L. Q., and N. A. Brunsell, 2013: The impact of temporal aggregation of land surface temperature data for surface urban heat island (SUHI) monitoring. *Remote Sensing of Environment*, **134**, 162–174, https://doi.org/10.1016/j.rse.2013.02.022.

Hu, L. Q., A. J. Monaghan, and N. A. Brunsell, 2015: Investigation of urban air temperature and humidity patterns during extreme heat conditions using satellite-derived data. *J. Appl. Meteorol. Climatol.*, **54**, 2245–2259, https://doi.org/10.1175/JAMC-D-15-0051.1.

Hu, L. Q., A. Monaghan, J. A. Voogt, and M. Barlage, 2016: A first satellite-based observational assessment of urban thermal anisotropy. *Remote Sensing of Environment*, **181**, 111–121, https://doi.org/10.1016/j.rse.2016.03.043.

Hu, L. Q., Y. Sun, G. Collins, and P. Fu, 2020: Improved estimates of monthly land surface temperature from MODIS using a diurnal temperature cycle (DTC) model. *ISPRS Journal of Photogrammetry and Remote Sensing*, **168**, 131–140, https://doi.org/10.1016/j.isprsjprs.2020.08.007.

Hu, Y. H., M. T. Hou, G. S. Jia, C. L. Zhao, X. J. Zhen, and Y. H. Xu, 2019: Comparison of surface and canopy urban heat islands within megacities of eastern China. *ISPRS Journal of Photogrammetry and Remote Sensing*, **156**, 160–168, https://doi.org/10.1016/j.isprsjprs.2019.08.012.

Hua, L. J., Z. G. Ma, and W. D. Guo, 2008: The impact of urbanization on air temperature across China. *Theor. Appl. Climatol.*, **93**, 179–194, https://doi.org/10.1007/s00704-007-0339-8.

Huang, M., Z. Q. Gao, S. G. Miao, and F. Chen, 2019: Sensitivity of urban boundary layer simulation to urban canopy models and PBL schemes in Beijing. *Meteorol. Atmos. Phys.*, **131**, 1235–1248, https://doi.org/10.1007/s00703-018-0634-1.

Huff, F. A., 1975: Urban effects on the distribution of heavy convective rainfall. *Water Resour. Res.*, **11**, 889–896, https://doi.org/10.1029/WR011i006p00889.

Huff, F. A., and S. A. Changnon Jr., 1972: Climatological assessment of urban effects on precipitation at St. *Louis. J. Appl. Meteorol. Climatol.*, **11**, 823–842, https://doi.org/10.1175/1520-0450(1972)011<0823:CAOUEO>2.0.CO;2.

Huff, F. A., and S. A. Changnon Jr., 1973: Precipitation modification by major urban areas. *Bull. Amer. Meteor. Soc.*, **54**,

1220–1233, https://doi.org/10.1175/1520-0477(1973)054<1220:PMBMUA>2.0.CO;2.

Huszar, P., and Coauthors, 2020: The impact of urban land-surface on extreme air pollution over central Europe. *Atmospheric Chemistry and Physics*, **20**, 11 655–11 681, https://doi.org/10.5194/acp-20-11655-2020.

Ichinose, T., K. Shimodozono, and K. Hanaki, 1999: Impact of anthropogenic heat on urban climate in Tokyo. *Atmos. Environ.*, **33**, 3897–3909, https://doi.org/10.1016/S1352-2310(99)00132-6.

Imhoff, M. L., P. Zhang, R. E. Wolfe, and L. Bounoua, 2010: Remote sensing of the urban heat island effect across biomes in the continental USA. *Remote Sensing of Environment*, **114**, 504–513, https://doi.org/10.1016/j.rse.2009.10.008.

Inamdar, A. K., A. French, S. Hook, G. Vaughan, and W. Luckett, 2008: Land surface temperature retrieval at high spatial and temporal resolutions over the southwestern United States. *J. Geophys. Res.*, **113**, D07107, https://doi.org/10.1029/2007JD009048.

Inoue, E., 1963: On the turbulent structure of airflow within. *J. Meteor. Soc. Japan*, **41**, 317–326, https://doi.org/10.2151/jmsj1923.41.6_317.

Inoue, T., and F. Kimura, 2004: Urban effects on low-level clouds around the Tokyo metropolitan area on clear summer days. *Geophys. Res. Lett.*, **31**, L05103, https://doi.org/10.1029/2003GL018908.

Ivajnšič, D., M. Kaligarič, and I. Žiberna, 2014: Geographically weighted regression of the urban heat island of a small city. *Applied Geography*, **53**, 341–353, https://doi.org/10.1016/j.apgeog.2014.07.001.

Jha, A. K., R. Bloch, and J. Lamond, 2012: *Cities and Flooding: A Guide to Integrated Urban Flood Risk Management for the 21st Century*. World Bank.

Jiang, P., Z. P. Wen, W. M. Sha, and G. X. Chen, 2017: Interaction between turbulent flow and sea breeze front over urban-like coast in large-eddy simulation. *J. Geophys. Res.*, **122**, 5298–5315, https://doi.org/10.1002/2016JD026247.

Jiang, S. J., K. C. Wang, and Y. N. Mao, 2020: Rapid local urbanization around most meteorological stations explains the observed daily asymmetric warming rates across China from 1985 to 2017. *J. Climate*, **33**, 9045–9061, https://doi.org/10.1175/JCLI-D-20-0118.1.

Jin, M. L., R. E. Dickinson, and A. M. Vogelmann, 1997: A comparison of CCM2–BATS skin temperature and surface-air temperature with satellite and surface observations. *J. Climate*, **10**, 1505–1524, https://doi.org/10.1175/1520-0442(1997)010<1505:ACOCBS>2.0.CO;2.

Jin, M. L., J. M. Shepherd, and M. D. King, 2005: Urban aerosols and their variations with clouds and rainfall: A case study for New York and Houston. *J. Geophys. Res.*, **110**, D10S20, https://doi.org/10.1029/2004JD005081.

Jin, M. L., J. M. Shepherd, and W. Z. Zheng, 2010: Urban surface temperature reduction via the urban aerosol direct effect: A remote sensing and WRF model sensitivity study. *Advances in Meteorology*, **2010**, 681587, https://doi.org/10.1155/2010/681587.

Johnson, G. T., Oke, T. R., Lyons, T. J., Steyn, D. G., Watson, I. D., and Voogt, J. A., 1991: Simulation of surface urban heat islands under 'IDEAL' conditions at night part 1: Theory and tests against field data. *Boundary-Layer Meteorology*, **56**(3), 275–294, https://doi.org/10.1007/BF00120424.

Jones, P. D., D. H. Lister, and Q. Li, 2008: Urbanization effects in large-scale temperature records, with an emphasis on China. *J. Geophys. Res.*, **113**, D16122, https://doi.org/10.1029/2008JD009916.

Kalnay, E., and M. Cai, 2003: Impact of urbanization and land-use change on climate. *Nature*, **423**, 528–531, https://doi.org/10.1038/nature01675.

Karlický, J., P. Huszár, T. Halenka, M. Belda, M. Žák, P. Pišoft, and J. Mikšovský, 2018: Multi-model comparison of urban heat island modelling approaches. *Atmospheric Chemistry and Physics*, **18**, 10 655–10 674, https://doi.org/10.5194/acp-18-10655-2018.

Karppinen, A., J. Kukkonen, T. Elolähde, M. Konttinen, T. Koskentalo, and E. Rantakrans, 2000: A modelling system for predicting urban air pollution: Model description and applications in the Helsinki metropolitan area. *Atmos. Environ.*, **34**, 3723–3733, https://doi.org/10.1016/S1352-2310(00)00074-1.

Kaufmann, R. K., K. C. Seto, A. Schneider, Z. T. Liu, L. M. Zhou, and W. L. Wang, 2007: Climate response to rapid urban growth: Evidence of a human-induced precipitation deficit. *J. Climate*, **20**, 2299–2306, https://doi.org/10.1175/JCLI4109.1.

Keeler, J. M., and D. A. R. Kristovich, 2012: Observations of urban heat island influence on lake-breeze frontal movement. *J. Appl. Meteorol. Climatol.*, **51**, 702–710, https://doi.org/10.1175/JAMC-D-11-0166.1.

Ketterer, C., and A. Matzarakis, 2014: Human-biometeorological assessment of the urban heat island in a city with complex topography–The case of Stuttgart, Germany. *Urban Climate*, **10**, 573–584, https://doi.org/10.1016/j.uclim.2014.01.003.

Ketterer, C., and A. Matzarakis, 2015: Comparison of different methods for the assessment of the urban heat island in Stuttgart, Germany. *International Journal of Biometeorology*, **59**, 1299–1309, https://doi.org/10.1007/s00484-014-0940-3.

Khain, A. P., N. BenMoshe, and A. Pokrovsky, 2008: Factors determining the impact of aerosols on surface precipitation from clouds: An attempt at classification. *J. Atmos. Sci.*, **65**, 1721–1748, https://doi.org/10.1175/2007JAS2515.1.

Kikegawa, Y., Y. Genchi, H. Yoshikado, and H. Kondo, 2003: Development of a numerical simulation system toward comprehensive assessments of urban warming countermeasures including their impacts upon the urban buildings' energy-demands. *Applied Energy*, **76**, 449–466, https://doi.org/10.1016/S0306-2619(03)00009-6.

Kim, H., Y.-K. Kim, S.-K. Song, and H. W. Lee, 2016: Impact of future urban growth on regional climate changes in the Seoul Metropolitan Area, Korea. *Science of the Total Environment*, **571**, 355–363, https://doi.org/10.1016/j.scitotenv.2016.05.046.

Kimura, F., and S. Takahashi, 1991: The effects of land-use and anthropogenic heating on the surface temperature in the Tokyo metropolitan area: A numerical experiment. *Atmospheric Environment. Part B. Urban Atmosphere*, **25**, 155–164, https://doi.org/10.1016/0957-1272(91)90050-O.

Kishtawal, C. M., D. Niyogi, M. Tewari, R. A. Pielke Sr, and J. M. Shepherd, 2010: Urbanization signature in the observed heavy rainfall climatology over India. *International Journal of Climatology*, **30**, 1908–1916, https://doi.org/10.1002/joc.2044.

Kolokotroni, M., B. L. Gowreesunker, and R. Giridharan, 2013: Cool roof technology in London: An experimental and modelling study. *Energy and Buildings*, **67**, 658–667, https://doi.org/10.1016/j.enbuild.2011.07.011.

Kondo, H., Y. Genchi, Y. Kikegawa, Y. Ohashi, H. Yoshikado, and H. Komiyama, 2005: Development of a multi-layer urban canopy model for the analysis of energy consumption in a big city: Structure of the urban canopy model and its basic performance. *Bound.-Layer Meteorol.*, **116**, 395–421, https://doi.org/10.1007/s10546-005-0905-5.

Kong, D. D., X. H. Gu, J. F. Li, G. Y. Ren, and J. Y. Liu, 2020: Contributions of global warming and urbanization to the intensification of human-perceived heatwaves over China. *J. Geophys. Res.*, **125**, e2019JD032175, https://doi.org/10.1029/2019JD032175.

Konstantinov, P., M. Varentsov, and I. Esau, 2018: A high density urban temperature network deployed in several cities of Eurasian Arctic. *Environmental Research Letters*, **13**, 075007, https://doi.org/10.1088/1748-9326/aacb84.

Kotharkar, R., A. Bagade, and A. Ramesh, 2019: Assessing urban drivers of canopy layer urban heat island: A numerical modeling approach. *Landscape and Urban Planning*, **190**, 103586, https://doi.org/10.1016/j.landurbplan.2019.05.017.

Kratzer, P. A., F. Vieweg, and S. Braunschweig, 1962: The climate of cities**,** American Meteorological Society.

Krayenhoff, E. S., and Coauthors, 2020: A multi-layer urban canopy meteorological model with trees (BEP-Tree): Street tree impacts on pedestrian-level climate. *Urban Climate*, **32**, 100590, https://doi.org/10.1016/j.uclim.2020.100590.

Krayenhoff, E. S., and Coauthors, 2021: Cooling hot cities: A systematic and critical review of the numerical modelling literature. *Environmental Research Letters*, **16**, 053007, https://doi.org/10.1088/1748-9326/abdcf1.

Kremer, A., 2020: The effect of urbanization on the intensity of heavy rainfall in the midwestern United States. Bachelor of Science, State University of New York College at Brockport, 39 pp.

Kumar, R., V. Mishra, J. Buzan, R. Kumar, D. Shindell, and M. Huber, 2017: Dominant control of agriculture and irrigation on urban heat island in India. *Scientific Reports*, **7**, 14054, https://doi.org/10.1038/s41598-017-14213-2.

Kumari, P., V. Garg, R. Kumar, and K. Kumar, 2021: Impact of urban heat island formation on energy consumption in Delhi. *Urban Climate*, **36**, 100763, https://doi.org/10.1016/j.uclim.2020.100763.

Kusaka, H., 2009: Performance of the WRF model as a high resolution regional climate model: Model intercomparison study. Preprints, *Proc. 7th Int. Conf. for Urban Climate*.

Kusaka, H., and F. Kimura, 2004: Coupling a single-layer urban canopy model with a simple atmospheric model: Impact on urban heat island simulation for an idealized case. *J. Meteor. Soc. Japan*, **82**, 67–80, https://doi.org/10.2151/jmsj.82.67.

Kusaka, H., F. Kimura, H. Hirakuchi, and M. Mizutori, 2000: The effects of land-use alteration on the sea breeze and daytime heat island in the Tokyo metropolitan area. *J. Meteor. Soc. Japan*, **78**, 405–420, https://doi.org/10.2151/jmsj1965.78.4_405.

Kusaka, H., H. Kondo, Y. Kikegawa, and F. Kimura, 2001: A simple single-layer urban canopy model for atmospheric models: Comparison with multi-layer and slab models. *Bound.-Layer Meteorol.*, **101**, 329–358, https://doi.org/10.1023/A:1019207923078.

Kusaka, H., K. Nawata, A. Suzuki-Parker, Y. Takane, and N. Furuhashi, 2014: Mechanism of precipitation increase with urbanization in Tokyo as revealed by ensemble climate simulations. *J. Appl. Meteorol. Climatol.*, **53**, 824–839, https://doi.org/10.1175/JAMC-D-13-065.1.

Kuttler, W., S. Weber, J. Schonnefeld, and A. Hesselschwerdt, 2007: Urban/rural atmospheric water vapour pressure differences and urban moisture excess in Krefeld, Germany. *International Journal of Climatology*, **27**, 2005–2015, https://doi.org/10.1002/joc.1558.

Lacke, M. C., T. L. Mote, and J. M. Shepherd, 2009: Aerosols and associated precipitation patterns in Atlanta. *Atmos. Environ.*, **43**, 4359–4373, https://doi.org/10.1016/j.atmosenv.2009.04.022.

Lai, D. Y., W. Y. Liu, T. T. Gan, K. X. Liu, and Q. Y. Chen, 2019: A review of mitigating strategies to improve the thermal environment and thermal comfort in urban outdoor spaces. *Science of the Total Environment*, **661**, 337–353, https://doi.org/10.1016/j.scitotenv.2019.01.062.

Landsberg, H. E., 1970: Man-made climatic changes: Man's activities have altered the climate of urbanized areas and may affect global climate in the future. *Science*, **170**, 1265–1274, https://doi.org/10.1126/science.170.3964.1265.

Lawrence, D. M., and Coauthors, 2019: The Community Land Model version 5: Description of new features, benchmarking, and impact of forcing uncertainty. *Journal of Advances in Modeling Earth Systems*, **11**, 4245–4287, https://doi.org/10.1029/2018MS001583.

Lee, S.-H., and S.-U. Park, 2008: A vegetated urban canopy model for meteorological and environmental modelling. *Bound.-Layer Meteorol.*, **126**, 73–102, https://doi.org/10.1007/s10546-007-9221-6.

Lee, S.-H., H. Lee, S.-B. Park, J.-W. Woo, D.-I. Lee, and J.-J. Baik, 2016: Impacts of in-canyon vegetation and canyon aspect ratio on the thermal environment of street canyons: Numerical investigation using a coupled WRF-VUCM model. *Quart. J. Roy. Meteor. Soc.*, **142**, 2562–2578, https://doi.org/10.1002/qj.2847.

Lemonsu, A., and V. Masson, 2002: Simulation of a summer urban breeze over Paris. *Bound.-Layer Meteorol.*, **104**, 463–490, https://doi.org/10.1023/A:1016509614936.

Li, D., and E. Bou-Zeid, 2013: Synergistic interactions between urban heat islands and heat waves: The impact in cities is larger than the sum of its parts. *J. Appl. Meteorol. Climatol.*, **52**, 2051–2064, https://doi.org/10.1175/JAMC-D-13-02.1.

Li, D., E. Bou-Zeid, and M. Oppenheimer, 2014: The effectiveness of cool and green roofs as urban heat island mitigation strategies. *Environmental Research Letters*, **9**, 055002, https://doi.org/10.1088/1748-9326/9/5/055002.

Li, D., S. Malyshev, and E. Shevliakova, 2016: Exploring historical and future urban climate in the Earth System Modeling framework: 1. Model development and evaluation. *Journal of Advances in Modeling Earth Systems*, **8**, 917–935, https://doi.org/10.1002/2015MS000578.

Li, D., W. L. Liao, A. J. Rigden, X. P. Liu, D. G. Wang, S. Malyshev, and E. Shevliakova, 2019: Urban heat island: Aerodynamics or imperviousness. *Science Advances*, **5**, eaau4299, https://doi.org/10.1126/sciadv.aau4299.

Li, H. D., Y. Y. Zhou, X. M. Li, L. Meng, X. Wang, S. Wu, and S. Sodoudi, 2018a: A new method to quantify surface urban heat island intensity. *Science of the Total Environment*, **624**, 262–272, https://doi.org/10.1016/j.scitotenv.2017.11.360.

Li, H. D., F. Meier, X. Lee, T. Chakraborty, J. F. Liu, M. Schaap, and S. Sodoudi, 2018b: Interaction between urban heat island and urban pollution island during summer in Berlin. *Science of the Total Environment*, **636**, 818−828, https://doi.org/10.1016/j.scitotenv.2018.04.254.

Li, J. F., Z. Feng, Y. Qian, and L. R. Leung, 2021: A high-resolution unified observational data product of mesoscale convective systems and isolated deep convection in the United States for 2004–2017. *Earth System Science Data*, **13**, 827–856, https://doi.org/10.5194/essd-13-827-2021.

Li, Q., H. Zhang, X. Liu, and J. Huang, 2004: Urban heat island effect on annual mean temperature during the last 50 years in China. *Theor. Appl. Climatol.*, **79**, 165−174, https://doi.org/10.1007/s00704-004-0065-4.

Li, W. B., and Coauthors, 2011: Urbanization signatures in strong versus weak precipitation over the Pearl River Delta metropolitan regions of China. *Environmental Research Letters*, **6**, 034020, https://doi.org/10.1088/1748-9326/6/3/034020.

Li, X. C., and Coauthors, 2020a: Mapping global urban boundaries from the global artificial impervious area (GAIA) data. *Environmental Research Letters*, **15**, 094044, https://doi.org/10.1088/1748-9326/ab9be3.

Li, X. M., Y. Y. Zhou, G. R. Asrar, M. Imhoff, and X. C. Li, 2017: The surface urban heat island response to urban expansion: A panel analysis for the conterminous United States. *Science of the Total Environment*, **605–606**, 426–435, https://doi.org/10.1016/j.scitotenv.2017.06.229.

Li, X.-X., and X. Liu, 2021: Effect of tree evapotranspiration and hydrological processes on urban microclimate in a tropical city: A WRF/SLUCM study. *Urban Climate 40*, 101009, https://doi.org/10.1016/j.uclim.2021.101009.

Li, Y. F., and Coauthors, 2020b: Strong intensification of hourly rainfall extremes by urbanization. *Geophys. Res. Lett.*, **47**, e2020GL088758, https://doi.org/10.1029/2020GL088758.

Liang, P., and Y. H. Ding, 2017: The long-term variation of extreme heavy precipitation and its link to urbanization effects in Shanghai during 1916–2014. *Adv. Atmos. Sci.*, **34**, 321−334, https://doi.org/10.1007/s00376-016-6120-0.

Liang, X., and Coauthors, 2018: SURF: Understanding and predicting urban convection and haze. *Bull. Amer. Meteor. Soc.*, **99**, 1391−1413, https://doi.org/10.1175/BAMS-D-16-0178.1.

Liao, J. B., T. J. Wang, X. M. Wang, M. Xie, Z. Q. Jiang, X. X. Huang, and J. L. Zhu, 2014: Impacts of different urban canopy schemes in WRF/Chem on regional climate and air quality in Yangtze River Delta, China. *Atmospheric Research*, **145–146**, 226–243, https://doi.org/10.1016/j.atmosres.2014.04.005.

Liao, W. L., D. G. Wang, X. P. Liu, G. L. Wang, and J. B. Zhang, 2017: Estimated influence of urbanization on surface warming in Eastern China using time-varying land use data. *International Journal of Climatology*, **37**, 3197–3208, https://doi.org/10.1002/joc.4908.

Liao, W. L., and Coauthors, 2018: Stronger contributions of urbanization to heat wave trends in wet climates. *Geophys. Res. Lett.*, **45**, 11 310–311 317, https://doi.org/10.1029/2018GL079679.

Lin, C.-Y., W.-C. Chen, S. C. Liu, Y. A. Liou, G. R. Liu, and T. H. Lin, 2008a: Numerical study of the impact of urbanization on the precipitation over Taiwan. *Atmos. Environ.*, **42**, 2934–2947, https://doi.org/10.1016/j.atmosenv.2007.12.054.

Lin, C.-Y., F. Chen, J. C. Huang, W.-C. Chen, Y.-A. Liou, W.-N. Chen, and S.-C. Liu, 2008b: Urban heat island effect and its impact on boundary layer development and land–sea circulation over northern Taiwan. *Atmos. Environ.*, **42**, 5635–5649, https://doi.org/10.1016/j.atmosenv.2008.03.015.

Lin, C.-Y., W.-C. Chen, P.-L. Chang, and Y.-F. Sheng, 2011: Impact of the urban heat island effect on precipitation over a complex geographic environment in northern Taiwan. *J. Appl. Meteorol. Climatol.*, **50**, 339–353, https://doi.org/10.1175/2010JAMC2504.1.

Lin, L. J., E. J. Ge, X. P. Liu, W. L. Liao, and M. Luo, 2018: Urbanization effects on heat waves in Fujian Province, Southeast China. *Atmospheric Research*, **210**, 123−132, https://doi.org/10.1016/j.atmosres.2018.04.011.

Lin, L. J., T. Gao, M. Luo, E. J. Ge, Y. J. Yang, Z. Liu, Y. Q. Zhao, and G. C. Ning, 2020: Contribution of urbanization to the changes in extreme climate events in urban agglomerations across China. *Science of the Total Environment*, **744**, 140264, https://doi.org/10.1016/j.scitotenv.2020.140264.

Lin, S., J. M. Feng, J. Wang, and Y. H. Hu, 2016: Modeling the contribution of long-term urbanization to temperature increase in three extensive urban agglomerations in China. *J. Geophys. Res.*, **121**, 1683−1697, https://doi.org/10.1002/2015JD024227.

Lin, X.-C., and S.-Q. Yu, 2005: Interdecadal changes of temperature in the Beijing region and its heat island effect. *Chinese Journal of Geophysics*, **48**, 47–54, https://doi.org/10.1002/cjg2.624.

Lin, Y., J. W. Fan, J.-H. Jeong, Y. W. Zhang, C. R. Homeyer, and J. Y. Wang, 2021: Urbanization-induced land and aerosol impacts on storm propagation and hail characteristics. *J. Atmos. Sci.*, **78**, 925–947, https://doi.org/10.1175/JAS-D-20-0106.1.

Lipson, M. J., M. Thatcher, M. A. Hart, and A. Pitman, 2018: A building energy demand and urban land surface model. *Quart. J. Roy. Meteor. Soc.*, **144**, 1572−1590, https://doi.org/10.1002/qj.3317.

Liu, J., and D. Niyogi, 2019: Meta-analysis of urbanization impact on rainfall modification. *Scientific Reports*, **9**, 7301, https://doi.org/10.1038/s41598-019-42494-2.

Liu, X., X.-X. Li, S. Harshan, M. Roth, and E. Velasco, 2017: Evaluation of an urban canopy model in a tropical city: The role of tree evapotranspiration. *Environmental Research Letters*, **12**, 094008, https://doi.org/10.1088/1748-9326/aa7ee7.

Liu, Y. B., F. Chen, T. Warner, and J. Basara, 2006: Verification of a mesoscale data-assimilation and forecasting system for the Oklahoma City area during the Joint Urban 2003 field project. *J. Appl. Meteorol. Climatol.*, **45**(7), 912–929, https://doi.org/10.1175/JAM2383.1.

Lo, C. P., D. A. Quattrochi, and J. C. Luvall, 1997: Application of high-resolution thermal infrared remote sensing and GIS to assess the urban heat island effect. *Int. J. Remote Sens.*, **18**, 287–304, https://doi.org/10.1080/014311697219079.

Lo, J. C. F., A. K. H. Lau, F. Chen, J. C. H. Fung, and K. K. M. Leung, 2007: Urban modification in a mesoscale model and the effects on the local circulation in the Pearl River Delta region. *J. Appl. Meteorol. Climatol.*, **46**, 457–476, https://doi.org/10.1175/JAM2477.1.

Lokoshchenko, M. A., 2017: Urban heat island and urban dry island in Moscow and their centennial changes. *J. Appl. Meteorol. Climatol.*, **56**, 2729–2745, https://doi.org/10.1175/JAMC-D-16-0383.1.

Louka, P., S. E. Belcher, and R. G. Harrison, 2000: Coupling between air flow in streets and the well-developed boundary layer aloft. *Atmos. Environ.*, **34**, 2613–2621, https://doi.org/10.1016/S1352-2310(99)00477-X.

Lowry, W. P., 1977: Empirical estimation of urban effects on climate: A problem analysis. *J. Appl. Meteorol. Climatol.*, **16**, 129–135, https://doi.org/10.1175/1520-0450(1977)016<0129:EEOUEO>2.0.CO;2.

Lowry, W. P., 1998: Urban effects on precipitation amount. *Progress in Physical Geography: Earth and Environment*, **22**, 477–520, https://doi.org/10.1177/030913339802200403.

Luo, M., and N.-C. Lau, 2017: Heat waves in southern China: Synoptic behavior, long-term change, and urbanization effects. *J. Climate*, **30**, 703–720, https://doi.org/10.1175/JCLI-D-16-0269.1.

Luo, M., and N.-C. Lau, 2018: Increasing heat stress in urban areas of eastern China: Acceleration by urbanization. *Geophys. Res. Lett.*, **45**, 13 060–13 069, https://doi.org/10.1029/2018GL080306.

Luo, M., and N.-C. Lau, 2021: Increasing human-perceived heat stress risks exacerbated by urbanization in China: A comparative study based on multiple metrics. *Earth's Future*, **9**, e2020EF001848, https://doi.org/10.1029/2020EF001848.

Lynn, B., A. Khain, D. Rosenfeld, and W. L. Woodley, 2007: Effects of aerosols on precipitation from orographic clouds. *J. Geophys. Res.*, **112**, D10225, https://doi.org/10.1029/2006JD007537.

Ma, H. Y., H. Y. Shao, and J. Song, 2014: Modeling the relative roles of the foehn wind and urban expansion in the 2002 Beijing heat wave and possible mitigation by high reflective roofs. *Meteorol. Atmos. Phys.*, **123**, 105–114, https://doi.org/10.1007/s00703-013-0289-x.

Ma, H. Y., Z. H. Jiang, J. Song, A. G. Dai, X. Q. Yang, and F. Huo, 2016: Effects of urban land-use change in East China on the East Asian summer monsoon based on the CAM5.1 model. *Climate Dyn.*, **46**, 2977–2989, https://doi.org/10.1007/s00382-015-2745-4.

Magli, S., C. Lodi, L. Lombroso, A. Muscio, and S. Teggi, 2015: Analysis of the urban heat island effects on building energy consumption. *International Journal of Energy and Environmental Engineering*, **6**, 91–99, https://doi.org/10.1007/s40095-014-0154-9.

Manoli, G., and Coauthors, 2019: Magnitude of urban heat islands largely explained by climate and population. *Nature*, **573**, 55–60, https://doi.org/10.1038/s41586-019-1512-9.

Marelle, L., G. Myhre, B. M. Steensen, Ø. Hodnebrog, K. Alterskjær, and J. Sillmann, 2020: Urbanization in megacities increases the frequency of extreme precipitation events far more than their intensity. *Environmental Research Letters*, **15**, 124072, https://doi.org/10.1088/1748-9326/abcc8f.

Maronga, B., and Coauthors, 2015: The Parallelized Large-Eddy Simulation Model (PALM) version 4.0 for atmospheric and oceanic flows: Model formulation, recent developments, and future perspectives. *Geoscientific Model Development*, **8**, 2515–2551, https://doi.org/10.5194/gmd-8-2515-2015.

Martilli, A., 2002: Numerical study of urban impact on boundary layer structure: Sensitivity to wind speed, urban morphology, and rural soil moisture. *J. Appl. Meteorol. Climatol.*, **41**, 1247–1266, https://doi.org/10.1175/1520-0450(2002)041<1247:NSOUIO>2.0.CO;2.

Martilli, A., 2014: An idealized study of city structure, urban climate, energy consumption, and air quality. *Urban Climate*, **10**, 430–446, https://doi.org/10.1016/j.uclim.2014.03.003.

Martilli, A., O. Brousse, and J. Ching, 2016: Urbanized WRF modeling using WUDAPT. Preprints, Technical Report March, Cientro de Investigaciones Energeticas MedioAmbientales y Tecnologicas (CIEMAT), 8. [Available online at http://www.wudapt.org/wp-content/uploads/2016/05/Urbanized-WRF-modeling-using-WUDAPT-web-version-March2016.pdf.]

Martilli, A., E. S. Krayenhoff, and N. Nazarian, 2020: Is the urban heat island intensity relevant for heat mitigation studies. *Urban Climate*, **31**, 100541, https://doi.org/10.1016/j.uclim.2019.100541.

Martin-Vide, J., P. Sarricolea, and M. C. Moreno-García, 2015: On the definition of urban heat island intensity: The "rural" reference. *Frontiers in Earth Science*, **3**, 24, https://doi.org/10.3389/feart.2015.00024.

Masson, V., 2000: A physically-based scheme for the urban energy budget in atmospheric models. *Bound.-Layer Meteorol.*, **94**, 357–397, https://doi.org/10.1023/A:1002463829265.

Masson, V., 2006: Urban surface modeling and the meso-scale impact of cities. *Theor. Appl. Climatol.*, **84**, 35–45, https://doi.org/10.1007/s00704-005-0142-3.

McLeod, J., M. Shepherd, and C. E. Konrad II, 2017: Spatio-temporal rainfall patterns around Atlanta, Georgia and possible relationships to urban land cover. *Urban Climate*, **21**, 27–42, https://doi.org/10.1016/j.uclim.2017.03.004.

McRae, G. J., W. R. Goodin, and J. H. Seinfeld, 1982: Development of a second-generation mathematical model for urban air pollution—I. *Model formulation. Atmos. Environ.*, **16**, 679–696, https://doi.org/10.1016/0004-6981(82)90386-9.

Meehl, G. A., and C. Tebaldi, 2004: More intense, more frequent, and longer lasting heat waves in the 21st century. *Science*, **305**, 994–997, https://doi.org/10.1126/science.1098704.

Meier, F., D. Fenner, T. Grassmann, M. Otto, and D. Scherer, 2017: Crowdsourcing air temperature from citizen weather stations for urban climate research. *Urban Climate*, **19**, 170–191, https://doi.org/10.1016/j.uclim.2017.01.006.

Meili, N., and Coauthors, 2020: An urban ecohydrological model to quantify the effect of vegetation on urban climate and hydrology (UT&C v1.0). *Geoscientific Model Development*, **13**, 335–362, https://doi.org/10.5194/gmd-13-335-2020.

Melchiorri, M., A. J. Florczyk, S. Freire, M. Schiavina, M. Pesaresi, and T. Kemper, 2018: Unveiling 25 years of planetary urbanization with remote sensing: Perspectives from the global human settlement layer. *Remote Sensing*, **10**, 768, https://doi.org/10.3390/rs10050768.

Menberg, K., P. Bayer, K. Zosseder, S. Rumohr, and P. Blum, 2013: Subsurface urban heat islands in German cities. *Science of the Total Environment*, **442**, 123–133, https://doi.org/10.1016/j.scitotenv.2012.10.043.

Meng, L., and Coauthors, 2020: Urban warming advances spring phenology but reduces the response of phenology to temperature in the conterminous United States. *Proceedings of the National Academy of Sciences of the United States of America*, **117**, 4228–4233, https://doi.org/10.1073/pnas.1911117117.

Miao, S. G., and F. Chen, 2008: Formation of horizontal convective rolls in urban areas. *Atmospheric Research*, **89**, 298–304, https://doi.org/10.1016/j.atmosres.2008.02.013.

Miao, S. G., F. Chen, M. A. LeMone, M. Tewari, Q. C. Li, and Y. C. Wang, 2009: An observational and modeling study of characteristics of urban heat island and boundary layer structures in Beijing. *J. Appl. Meteorol. Climatol.*, **48**, 484–501, https://doi.org/10.1175/2008JAMC1909.1.

Miao, S. G., F. Chen, Q. C. Li, and S. Y. Fan, 2011: Impacts of urban processes and urbanization on summer precipitation:

A case study of heavy rainfall in Beijing on 1 August 2006. *J. Appl. Meteorol. Climatol.*, **50**, 806–825, https://doi.org/10.1175/2010JAMC2513.1.

Miao, Y. C., S. H. Liu, B. C. Chen, B. H. Zhang, S. Wang, and S. Y. Li, 2013: Simulating urban flow and dispersion in Beijing by coupling a CFD model with the WRF model. *Adv. Atmos. Sci.*, **30**, 1663–1678, https://doi.org/10.1007/s00376-013-2234-9.

Miao, Y. C., S. H. Liu, Y. J. Zheng, S. Wang, and B. C. Chen, 2015: Numerical study of the effects of topography and urbanization on the local atmospheric circulations over the Beijing-Tianjin-Hebei, China. *Advances in Meteorology*, **2015**, 397070, https://doi.org/10.1155/2015/397070.

Michioka, T., A. Sato, and K. Sada, 2013: Large-eddy simulation coupled to mesoscale meteorological model for gas dispersion in an urban district. *Atmos. Environ.*, **75**, 153–162, https://doi.org/10.1016/j.atmosenv.2013.04.017.

Middel, A., S. Alkhaled, F. A. Schneider, B. Hagen, and P. Coseo, 2021: 50 Grades of Shade. *Bull. Amer. Meteor. Soc.*, E1805–E1820, https://doi.org/10.1175/BAMS-D-20-0193.1.

Mills, G., 1997: An urban canopy-layer climate model. *Theor. Appl. Climatol.*, **57**, 229–244, https://doi.org/10.1007/BF00863615.

Mitra, C., and J. M. Shepherd, 2015: Urban precipitation: A global perspective. *The Routledge Handbook of Urbanization and Global Environmental Change*, K. C.-Y. Seto, W. Solecki and C. Griffith, Eds., Routledge, 176–192, https://doi.org/10.4324/9781315849256.

Moriwaki, R., K. Watanabe, and K. Morimoto, 2013: Urban dry island phenomenon and its impact on cloud base level. *Journal of JSCE*, **1**, 521–529, https://doi.org/10.2208/journalofjsce.1.1_521.

Mote, T. L., M. C. Lacke, and J. M. Shepherd, 2007: Radar signatures of the urban effect on precipitation distribution: A case study for Atlanta, Georgia. *Geophys. Res. Lett.*, **34**, L20710, https://doi.org/10.1029/2007GL031903.

Mughal, M. O., X.-X. Li, T. G. Yin, A. Martilli, O. Brousse, M. A. Dissegna, and L. K. Norford, 2019: High-resolution, multilayer modeling of Singapore's urban climate incorporating local climate zones. *J. Geophys. Res.*, **124**, 7764–7785, https://doi.org/10.1029/2018JD029796.

Mughal, M. O., X.-X. Li, and L. K. Norford, 2020: Urban heat island mitigation in Singapore: Evaluation using WRF/multilayer urban canopy model and local climate zones. *Urban Climate*, **34**, 100714, https://doi.org/10.1016/j.uclim.2020.100714.

Muller, C. L., L. Chapman, C. S. B. Grimmond, D. T. Young, and X. M. Cai, 2013: Sensors and the city: A review of urban meteorological networks. *International Journal of Climatology*, **33**, 1585–1600, https://doi.org/10.1002/joc.3678.

Muller, C. L., L. Chapman, S. Johnston, C. Kidd, S. Illingworth, G. Foody, A. Overeem, and R. R. Leigh, 2015: Crowdsourcing for climate and atmospheric sciences: Current status and future potential. *International Journal of Climatology*, **35**, 3185–3203, https://doi.org/10.1002/joc.4210.

Mussetti, G., and Coauthors, 2020: COSMO-BEP-Tree v1.0: A coupled urban climate model with explicit representation of street trees. *Geoscientific Model Development*, **13**, 1685–1710, https://doi.org/10.5194/gmd-13-1685-2020.

Myrup, L. O., 1969: A numerical model of the urban heat island. *J. Appl. Meteorol. Climatol.*, **8**, 908–918, https://doi.org/10.1175/1520-0450(1969)008<0908:ANMOTU>2.0.CO;2.

Nadeau, D. F., and Coauthors, 2009: Estimation of urban sensible heat flux using a dense wireless network of observations. *Environmental Fluid Mechanics*, **9**, 635–653, https://doi.org/10.1007/s10652-009-9150-7.

Napoly, A., T. Grassmann, F. Meier, and D. Fenner, 2018: Development and application of a statistically-based quality control for crowdsourced air temperature data. *Frontiers in Earth Science*, **6**, 118, https://doi.org/10.3389/feart.2018.00118.

Naughton, J., and W. McDonald, 2019: Evaluating the variability of urban land surface temperatures using drone observations. *Remote Sensing*, **11**, 1722, https://doi.org/10.3390/rs11141722.

Nazarian, N., and Coauthors, 2021: Project Coolbit: Can your watch predict heat stress and thermal comfort sensation. *Environmental Research Letters*, **16**, 034031, https://doi.org/10.1088/1748-9326/abd130.

Ngie, A., K. Abutaleb, F. Ahmed, A. Darwish, and M. Ahmed, 2014: Assessment of urban heat island using satellite remotely sensed imagery: A review. *South African Geographical Journal*, **96**, 198–214, https://doi.org/10.1080/03736245.2014.924864.

Niyogi, D., P. Pyle, M. Lei, S. P. Arya, C. M. Kishtawal, M. Shepherd, F. Chen, and B. Wolfe, 2011: Urban modification of thunderstorms: An observational storm climatology and model case study for the Indianapolis urban region. *J. Appl. Meteorol. Climatol.*, **50**, 1129–1144, https://doi.org/10.1175/2010JAMC1836.1.

Niyogi, D., M. Lei, C. Kishtawal, P. Schmid, and M. Shepherd, 2017: Urbanization impacts on the summer heavy rainfall climatology over the eastern United States. *Earth Interactions*, **21**, 1–17, https://doi.org/10.1175/EI-D-15-0045.1.

Nowak, D. J., and D. E. Crane, 2000: The Urban Forest Effects (UFORE) Model: Quantifying urban forest structure and functions. *Integrated Tools for Natural Resources Inventories in the Twenty-first Century*, M. Hansen and T. Burk, Eds., US Department of Agriculture, Forest Service, North Central Forest Experiment Station, 714–720.

Ntelekos, A. A., J. A. Smith, L. Donner, J. D. Fast, W. I. Gustafson Jr., E. G. Chapman, and W. F. Krajewski, 2009: The effects of aerosols on intense convective precipitation in the northeastern United States. *Quart. J. Roy. Meteor. Soc.*, **135**, 1367–1391, https://doi.org/10.1002/qj.476.

O'Malley, C., P. Piroozfar, E. R. P. Farr, and F. Pomponi, 2015: Urban Heat Island (UHI) mitigating strategies: A case-based comparative analysis. *Sustainable Cities and Society*, **19**, 222–235, https://doi.org/10.1016/j.scs.2015.05.009.

Offerle, B., C. S. B. Grimmond, and T. R. Oke, 2003: Parameterization of net all-wave radiation for urban areas. *J. Appl. Meteorol. Climatol.*, **42**, 1157–1173, https://doi.org/10.1175/1520-0450(2003)042<1157:PONARF>2.0.CO;2.

Ogden, T. L., 1969: The effect of rainfall on a large steelworks. *J. Appl. Meteorol. Climatol.*, **8**, 585–591, https://doi.org/10.1175/1520-0450(1969)008<0585:TEOROA>2.0.CO;2.

Oke, T., 1969: Towards a more rational understanding of the urban heat island. *McGill Climatological Bulletin*, **3**, 1–21.

Oke, T. R., 1976: The distinction between canopy and boundary-layer urban heat islands. *Atmosphere*, **14**, 268–277, https://doi.org/10.1080/00046973.1976.9648422.

Oke, T. R., 1981: Canyon geometry and the nocturnal urban heat island: Comparison of scale model and field observations. *J. Climatol.*, **1**, 237–254, https://doi.org/10.1002/joc.33700

10304.

Oke, T. R., 1982a: The energetic basis of the urban heat island. *Quart. J. Roy. Meteor. Soc.*, **108**, 1–24, https://doi.org/10.1002/qj.49710845502.

Oke, T. R., 1982b: *Bibliography of Urban Climate 1977–1980*. World Meteorological Organization.

Oke, T. R., 1995: The heat island of the urban boundary layer: Characteristics, causes and effects. *Wind Climate in Cities*, J. E. Cermak et al., Eds., Springer, 81–107, https://doi.org/10.1007/978-94-017-3686-2_5.

Oke, T. R., 2006: Towards better scientific communication in urban climate. *Theor. Appl. Climatol.*, **84**, 179–190, https://doi.org/10.1007/s00704-005-0153-0.

Oke, T. R., and C. East, 1971: The urban boundary layer in Montreal. *Bound.-Layer Meteorol.*, **1**, 411–437, https://doi.org/10.1007/BF00184781.

Oke, T. R., and H. A. Cleugh, 1987: Urban heat storage derived as energy balance residuals. *Bound.-Layer Meteorol.*, **39**, 233–245, https://doi.org/10.1007/BF00116120.

Oke, T. R., G. Mills, A. Christen, and J. A. Voogt, 2017: *Urban Climates*. Cambridge University Press, https://doi.org/10.1017/9781139016476.

Oleson, K., 2012: Contrasts between urban and rural climate in CCSM4 CMIP5 climate change scenarios. *J. Climate*, **25**, 1390–1412, https://doi.org/10.1175/JCLI-D-11-00098.1.

Oleson, K., and J. Feddema, 2020: Parameterization and surface data improvements and new capabilities for the Community Land Model Urban (CLMU). *Journal of Advances in Modeling Earth Systems*, **12**, e2018MS001586, https://doi.org/10.1029/2018MS001586.

Oleson, K. W., G. B. Bonan, J. Feddema, M. Vertenstein, and C. Grimmond, 2008: An urban parameterization for a global climate model. Part I: Formulation and evaluation for two cities. *J. Appl. Meteorol. Climatol.*, **47**, 1038–1060, https://doi.org/10.1175/2007JAMC1597.1.

Oleson, K. W., G. B. Bonan, and J. Feddema, 2010: Effects of white roofs on urban temperature in a global climate model. *Geophys. Res. Lett.*, **37**, L03701, https://doi.org/10.1029/2009GL042194.

Oleson, K. W., G. B. Bonan, J. Feddema, and T. Jackson, 2011: An examination of urban heat island characteristics in a global climate model. *International Journal of Climatology*, **31**, 1848–1865, https://doi.org/10.1002/joc.2201.

Oliveira, A., A. Lopes, E. Correia, S. Niza, and A. Soares, 2021: An urban climate-based empirical model to predict present and future patterns of the Urban Thermal Signal. *Science of the Total Environment*, **790**, 147710, https://doi.org/10.1016/j.scitotenv.2021.147710.

Omidvar, H., E. Bou-Zeid, Q. Li, J.-P. Mellado, and P. Klein, 2020: Plume or bubble. *Mixed-convection flow regimes and city-scale circulations. J. Fluid Mech.*, **897**, A5, https://doi.org/10.1017/jfm.2020.360.

Otte, T. L., A. Lacser, S. Dupont, and J. K. S. Ching, 2004: Implementation of an urban canopy parameterization in a mesoscale meteorological model. *J. Appl. Meteorol. Climatol.*, **43**, 1648–1665, https://doi.org/10.1175/JAM2164.1.

Owen, T. W., 1998: Using DMSP-OLS light frequency data to categorize urban environments associated with US climate observing stations. *Int. J. Remote Sens.*, **19**, 3451–3456, https://doi.org/10.1080/014311698214127.

Pandey, A. K., and Coauthors, 2014: Spatio–temporal variations of urban heat island over Delhi. *Urban Climate*, **10**, 119–133, https://doi.org/10.1016/j.uclim.2014.10.005.

Pantavou, K., S. Lykoudis, M. Nikolopoulou, and I. X. Tsiros, 2018: Thermal sensation and climate: A comparison of UTCI and PET thresholds in different climates. *International Journal of Biometeorology*, **62**, 1695–1708, https://doi.org/10.1007/s00484-018-1569-4.

Pappaccogli, G., L. Giovannini, D. Zardi, and A. Martilli, 2020: Sensitivity analysis of urban microclimatic conditions and building energy consumption on urban parameters by means of idealized numerical simulations. *Urban Climate*, **34**, 100677, https://doi.org/10.1016/j.uclim.2020.100677.

Paschalis, A., T. C. Chakraborty, S. Fatichi, N. Meili, and G. Manoli, 2021: Urban forests as main regulator of the evaporative cooling effect in cities. *AGU Advances*, **2**, e2020AV000303, https://doi.org/10.1029/2020AV000303.

Peng, S. S., and Coauthors, 2012: Surface urban heat island across 419 global big cities. *Environ. Sci. Technol.*, **46**, 696–703, https://doi.org/10.1021/es2030438.

Peron, F., M. M. De Maria, F. Spinazzè, and U. Mazzali, 2015: An analysis of the urban heat island of Venice mainland. *Sustainable Cities and Society*, **19**, 300–309, https://doi.org/10.1016/j.scs.2015.05.008.

Peterson, T. C., 2003: Assessment of urban versus rural in situ surface temperatures in the contiguous United States: No difference found. *J. Climate*, **16**, 2941–2959, https://doi.org/10.1175/1520-0442(2003)016<2941:AOUVRI>2.0.CO;2.

Peterson, T. C., and R. S. Vose, 1997: An overview of the global historical climatology network temperature database. *Bull. Amer. Meteor. Soc.*, **78**, 2837–2850, https://doi.org/10.1175/1520-0477(1997)078<2837:AOOTGH>2.0.CO;2.

Peterson, T. C., and T. W. Owen, 2005: Urban heat island assessment: Metadata are important. *J. Climate*, **18**, 2637–2646, https://doi.org/10.1175/JCLI3431.1.

Peterson, T. C., K. P. Gallo, J. Lawrimore, T. W. Owen, A. Huang, and D. A. McKittrick, 1999: Global rural temperature trends. *Geophys. Res. Lett.*, **26**, 329–332, https://doi.org/10.1029/1998GL900322.

Piringer, M., and Coauthors, 2002: Investigating the surface energy balance in urban areas–recent advances and future needs. *Water, Air and Soil Pollution: Focus*, **2**, 1–16, https://doi.org/10.1023/A:1021302824331.

Potere, D., and A. Schneider, 2007: A critical look at representations of urban areas in global maps. *GeoJournal*, **69**, 55–80, https://doi.org/10.1007/s10708-007-9102-z.

Priyadarsini, R., W. N. Hien, and C. K. W. David, 2008: Microclimatic modeling of the urban thermal environment of Singapore to mitigate urban heat island. *Solar Energy*, **82**, 727–745, https://doi.org/10.1016/j.solener.2008.02.008.

Quan, J. L., Y. H. Chen, W. F. Zhan, J. F. Wang, J. Voogt, and M. J. Wang, 2014: Multi-temporal trajectory of the urban heat island centroid in Beijing, China based on a Gaussian volume model. *Remote Sensing of Environment*, **149**, 33–46, https://doi.org/10.1016/j.rse.2014.03.037.

Radhi, H., F. Fikry, and S. Sharples, 2013: Impacts of urbanisation on the thermal behaviour of new built up environments: A scoping study of the urban heat island in Bahrain. *Landscape and Urban Planning*, **113**, 47–61, https://doi.org/10.1016/j.landurbplan.2013.01.013.

Radhi, H., S. Sharples, and E. Assem, 2015: Impact of urban heat islands on the thermal comfort and cooling energy demand of artificial islands—A case study of AMWAJ Islands in Bahrain. *Sustainable Cities and Society*, **19**, 310–318, https://doi.org/10.1016/j.scs.2015.07.017.

Rajagopalan, P., K. C. Lim, and E. Jamei, 2014: Urban heat island and wind flow characteristics of a tropical city. *Solar Energy*, **107**, 159–170, https://doi.org/10.1016/j.solener.2014.05.042.

Ramamurthy, P., and E. Bou-Zeid, 2017: Heatwaves and urban heat islands: A comparative analysis of multiple cities. *J. Geophys. Res.*, **122**, 168–178, https://doi.org/10.1002/2016JD025357.

Rasul, A., H. Balzter, and C. Smith, 2016: Diurnal and seasonal variation of surface urban cool and heat islands in the semi-arid city of Erbil, Iraq. *Climate*, **4**, 42, https://doi.org/10.3390/cli4030042.

Rasul, A., H. Balzter, C. Smith, J. Remedios, B. Adamu, J. A. Sobrino, M. Srivanit, and Q. H. Weng, 2017: A review on remote sensing of urban heat and cool islands. *Land*, **6**, 38, https://doi.org/10.3390/land6020038.

Redon, E., A. Lemonsu, and V. Masson, 2020: An urban trees parameterization for modeling microclimatic variables and thermal comfort conditions at street level with the Town Energy Balance model (TEB-SURFEX v8.0). *Geoscientific Model Development*, **13**, 385–399, https://doi.org/10.5194/gmd-13-385-2020.

Ren, G. Y., and Y. Q. Zhou, 2014: Urbanization effect on trends of extreme temperature indices of national stations over mainland China, 1961–2008. *J. Climate*, **27**, 2340–2360, https://doi.org/10.1175/JCLI-D-13-00393.1.

Ren, G. Y., Z. Y. Chu, Z. H. Chen, and Y. Y. Ren, 2007: Implications of temporal change in urban heat island intensity observed at Beijing and Wuhan stations. *Geophys. Res. Lett.*, **34**, L05711, https://doi.org/10.1029/2006GL027927.

Ren, G. Y., Y. Q. Zhou, Z. Y. Chu, J. X. Zhou, A. Y. Zhang, J. Guo, and X. F. Liu, 2008: Urbanization effects on observed surface air temperature trends in North China. *J. Climate*, **21**, 1333–1348, https://doi.org/10.1175/2007JCLI1348.1.

Resler, J., and Coauthors, 2017: PALM-USM v1.0: A new urban surface model integrated into the PALM large-eddy simulation model. *Geoscientific Model Development*, **10**, 3635–3659, https://doi.org/10.5194/gmd-10-3635-2017.

Ribeiro, F. N. D., A. P. de Oliveira, J. Soares, R. M. de Miranda, M. Barlage, and F. Chen, 2018: Effect of sea breeze propagation on the urban boundary layer of the metropolitan region of Sao Paulo, Brazil. *Atmospheric Research*, **214**, 174–188, https://doi.org/10.1016/j.atmosres.2018.07.015.

Rizwan, A. M., L. Y. C. Dennis, and C. Liu, 2008: A review on the generation, determination and mitigation of Urban Heat Island. *Journal of Environmental Sciences*, **20**(1), 120–128, https://doi.org/10.1016/S1001-0742(08)60019-4.

Robaa, S. M., 2013: Some aspects of the urban climates of Greater Cairo Region, Egypt. *International Journal of Climatology*, **33**, 3206–3216, https://doi.org/10.1002/joc.3661.

Rodríguez, L. R., J. S. Ramos, F. J. S. de la Flor, and S. Á. Domínguez, 2020: Analyzing the urban heat Island: Comprehensive methodology for data gathering and optimal design of mobile transects. *Sustainable Cities and Society*, **55**, 102027, https://doi.org/10.1016/j.scs.2020.102027.

Rosenfeld, D., 2000: Suppression of rain and snow by urban and industrial air pollution. *Science*, **287**, 1793–1796, https://doi.org/10.1126/science.287.5459.1793.

Rosenfeld, D., and A. Givati, 2006: Evidence of orographic precipitation suppression by air pollution–induced aerosols in the western United States. *J. Appl. Meteorol. Climatol.*, **45**, 893–911, https://doi.org/10.1175/JAM2380.1.

Rosenfeld, D., U. Lohmann, G. B. Raga, C. D. O'Dowd, M. Kulmala, S. Fuzzi, A. Reissell, and M. O. Andreae, 2008: Flood or drought: How do aerosols affect precipitation. *Science*, **321**, 1309–1313, https://doi.org/10.1126/science.1160606.

Rotach, M. W., 1993a: Turbulence close to a rough urban surface Part I: Reynolds stress. *Bound.-Layer Meteorol.*, **65**, 1–28, https://doi.org/10.1007/BF00708816.

Rotach, M. W., 1993b: Turbulence close to a rough urban surface part II: Variances and gradients. *Bound.-Layer Meteorol.*, **66**, 75–92, https://doi.org/10.1007/BF00705460.

Roth, M., 2000: Review of atmospheric turbulence over cities. *Quart. J. Roy. Meteor. Soc.*, **126**, 941–990, https://doi.org/10.1002/qj.49712656409.

Roth, M., 2007: Review of urban climate research in (sub) tropical regions. *International Journal of Climatology*, **27**, 1859–1873, https://doi.org/10.1002/joc.1591.

Rozenfeld, H. D., D. Rybski, J. S. Andrade Jr., M. Batty, H. E. Stanley, and H. A. Makse, 2008: Laws of population growth. *Proceedings of the National Academy of Sciences of the United States of America*, **105**, 18 702–18 707, https://doi.org/10.1073/pnas.0807435105.

Rozoff, C. M., W. R. Cotton, and J. O. Adegoke, 2003: Simulation of St. Louis, Missouri, land use impacts on thunderstorms. *J. Appl. Meteorol. Climatol.*, **42**, 716–738, https://doi.org/10.1175/1520-0450(2003)042<0716:SOSLML>2.0.CO;2.

Ryu, Y.-H., E. Bou-Zeid, Z.-H. Wang, and J. A. Smith, 2016a: Realistic representation of trees in an urban canopy model. *Bound.-Layer Meteorol. 159*, 193–220, https://doi.org/10.1007/s10546-015-0120-y.

Ryu, Y.-H., J. A. Smith, E. Bou-Zeid, and M. L. Baeck, 2016b: The influence of land surface heterogeneities on heavy convective rainfall in the Baltimore–Washington metropolitan area. *Mon. Wea. Rev.*, **144**, 553–573, https://doi.org/10.1175/MWR-D-15-0192.1.

Sachindra, D. A., A. W. M. Ng, S. Muthukumaran, and B. J. C. Perera, 2016: Impact of climate change on urban heat island effect and extreme temperatures: A case-study. *Quart. J. Roy. Meteor. Soc.*, **142**, 172–186, https://doi.org/10.1002/qj.2642.

Saitoh, T. S., T. Shimada, and H. Hoshi, 1996: Modeling and simulation of the Tokyo urban heat island. *Atmos. Environ.*, **30**, 3431–3442, https://doi.org/10.1016/1352-2310(95)00489-0.

Salamanca, F., and A. Martilli, 2010: A new building energy model coupled with an urban canopy parameterization for urban climate simulations—Part II. Validation with one dimension off-line simulations. *Theor. Appl. Climatol.*, **99**, 345–356, https://doi.org/10.1007/s00704-009-0143-8.

Salamanca, F., A. Krpo, A. Martilli, and A. Clappier, 2010: A new building energy model coupled with an urban canopy parameterization for urban climate simulations—part I. Formulation, verification, and sensitivity analysis of the model. *Theor. Appl. Climatol.*, **99**, 331–344, https://doi.org/10.1007/s00704-009-0142-9.

Salamanca, F., A. Martilli, M. Tewari, and F. Chen, 2011: A study of the urban boundary layer using different urban parameterizations and high-resolution urban canopy parameters with WRF. *J. Appl. Meteorol. Climatol.*, **50**, 1107–1128, https://doi.org/10.1175/2010JAMC2538.1.

Salamanca, F., Y. Z. Zhang, M. Barlage, F. Chen, A. Mahalov, and S. G. Miao, 2018: Evaluation of the WRF-urban modeling system coupled to Noah and Noah-MP land surface models over a semiarid urban environment. *J. Geophys. Res.*, **123**, 2387–2408, https://doi.org/10.1002/2018JD028377.

Sang, J. G., H. P. Liu, H. Z. Liu, and Z. K. Zhang, 2000: Observational and numerical studies of wintertime urban boundary layer. *Journal of Wind Engineering and Industrial Aerodynamics*, **87**, 243–258, https://doi.org/10.1016/S0167-6105(00)00040-4.

Santamouris, M., 2014: Cooling the cities–a review of reflective and green roof mitigation technologies to fight heat island and improve comfort in urban environments. *Solar Energy*, **103**, 682–703, https://doi.org/10.1016/j.solener.2012.07.003.

Santamouris, M., 2015: Analyzing the heat island magnitude and characteristics in one hundred Asian and Australian cities and regions. *Science of the Total Environment*, **512–513**, 582–598, https://doi.org/10.1016/j.scitotenv.2015.01.060.

Santiago, J. L., and A. Martilli, 2010: A dynamic urban canopy parameterization for mesoscale models based on computational fluid dynamics Reynolds-averaged Navier–Stokes microscale simulations. *Bound.-Layer Meteorol.*, **137**, 417–439, https://doi.org/10.1007/s10546-010-9538-4.

Sarangi, C., S. N. Tripathi, Y. Qian, S. Kumar, and L. Ruby Leung, 2018: Aerosol and urban land use effect on rainfall around cities in Indo-Gangetic Basin from observations and cloud resolving model simulations. *J. Geophys. Res.*, **123**, 3645–3667, https://doi.org/10.1002/2017JD028004.

Scheffe, R. D., and R. E. Morris, 1993: A review of the development and application of the urban airshed model. *Atmospheric Environment. Part B. Urban Atmosphere*, **27**, 23–39, https://doi.org/10.1016/0957-1272(93)90043-6.

Scherba, A., D. J. Sailor, T. N. Rosenstiel, and C. C. Wamser, 2011: Modeling impacts of roof reflectivity, integrated photovoltaic panels and green roof systems on sensible heat flux into the urban environment. *Building and Environment*, **46**, 2542–2551, https://doi.org/10.1016/j.buildenv.2011.06.012.

Schickedanz, P. T., 1974: Inadvertent rain modification as indicated by surface raincells. *J. Appl. Meteorol. Climatol.*, **13**, 891–900, https://doi.org/10.1175/1520-0450(1974)013<0891:IRMAIB>2.0.CO;2.

Schlünzen, K., P. Hoffmann, G. Rosenhagen, and W. Riecke, 2010: Long-term changes and regional differences in temperature and precipitation in the metropolitan area of Hamburg. *International Journal of Climatology*, **30**, 1121–1136, https://doi.org/10.1002/joc.1968.

Schmid, P. E., and D. Niyogi, 2017: Modeling urban precipitation modification by spatially heterogeneous aerosols. *J. Appl. Meteorol. Climatol.*, **56**, 2141–2153, https://doi.org/10.1175/JAMC-D-16-0320.1.

Schubert, S., S. Grossman-Clarke, and A. Martilli, 2012: A double-canyon radiation scheme for multi-layer urban canopy models. *Bound.-Layer Meteorol.*, **145**, 439–468, https://doi.org/10.1007/s10546-012-9728-3.

Scott, A. A., D. W. Waugh, and B. F. Zaitchik, 2018: Reduced urban heat island intensity under warmer conditions. *Environmental Research Letters*, **13**, 064003, https://doi.org/10.1088/1748-9326/aabd6c.

Semonin, R. G., 1981: Cloud characteristics. *Metromex: A Review and Summary*, S. A. Changnon Jr., Ed., Springer, 63–73, https://doi.org/10.1007/978-1-935704-29-4_4.

Sen Roy, S., and F. Yuan, 2009: Trends in extreme temperatures in relation to urbanization in the Twin Cities Metropolitan Area, Minnesota. *J. Appl. Meteorol. Climatol.*, **48**, 669–679, https://doi.org/10.1175/2008JAMC1983.1.

Shao, H. Y., J. Song, and H. Y. Ma, 2013: Sensitivity of the East Asian summer monsoon circulation and precipitation to an idealized large-scale urban expansion. *J. Meteor. Soc. Japan*, **91**, 163–177, https://doi.org/10.2151/jmsj.2013-205.

Sharma, A., P. Conry, H. J. S. Fernando, A. F. Hamlet, J. J. Hellmann, and F. Chen, 2016: Green and cool roofs to mitigate urban heat island effects in the Chicago metropolitan area: Evaluation with a regional climate model. *Environmental Research Letters*, **11**, 064004, https://doi.org/10.1088/1748-9326/11/6/064004.

Sharma, A., S. Woodruff, M. Budhathoki, A. F. Hamlet, F. Chen, and H. J. S. Fernando, 2018: Role of green roofs in reducing heat stress in vulnerable urban communities—A multidisciplinary approach. *Environmental Research Letters*, **13**, 094011, https://doi.org/10.1088/1748-9326/aad93c.

Sharma, A., D. J. Wuebbles, R. Kotamarthi, K. Calvin, B. Drewniak, C. E. Catlett, and R. Jacob, 2020: Urban-scale processes in high-spatial-resolution earth system models. *Bull. Amer. Meteor. Soc.*, **101**, E1555–E1561, https://doi.org/10.1175/BAMS-D-20-0114.1.

Shem, W., and M. Shepherd, 2009: On the impact of urbanization on summertime thunderstorms in Atlanta: Two numerical model case studies. *Atmospheric Research*, **92**, 172–189, https://doi.org/10.1016/j.atmosres.2008.09.013.

Shen, L. D., J. N. Sun, and R. M. Yuan, 2018: Idealized large-eddy simulation study of interaction between urban heat island and sea breeze circulations. *Atmospheric Research*, **214**, 338–347, https://doi.org/10.1016/j.atmosres.2018.08.010.

Shepherd, J. M., 2013: Impacts of urbanization on precipitation and storms: Physical insights and vulnerabilities. *Climate Vulnerability*, **5**, 109–125, https://doi.org/10.1016/B978-0-12-384703-4.00503-7.

Shepherd, J. M., H. Pierce, and A. J. Negri, 2002: Rainfall modification by major urban areas: Observations from spaceborne rain radar on the TRMM satellite. *J. Appl. Meteorol. Climatol.*, **41**, 689–701, https://doi.org/10.1175/1520-0450(2002)041<0689:RMBMUA>2.0.CO;2.

Shepherd, J. M., M. Carter, M. Manyin, D. Messen, and S. Burian, 2010: The impact of urbanization on current and future coastal precipitation: A case study for Houston. *Environment and Planning B: Planning and Design*, **37**, 284–304, https://doi.org/10.1068/b34102t.

Simpson, M., S. Raman, R. Suresh, and U. C. Mohanty, 2008: Urban effects of Chennai on sea breeze induced convection and precipitation. *Journal of Earth System Science*, **117**, 897–909, https://doi.org/10.1007/s12040-008-0075-1.

Singh, J., S. Karmakar, D. PaiMazumder, S. Ghosh, and D. Niyogi, 2020: Urbanization alters rainfall extremes over the contiguous United States. *Environmental Research Letters*, **15**, 074033, https://doi.org/10.1088/1748-9326/ab8980.

Sodoudi, S., P. Shahmohamadi, K. Vollack, U. Cubasch, and A. I. Che-Ani, 2014: Mitigating the urban heat island effect in megacity Tehran. *Advances in Meteorology*, **2014**, 547974, https://doi.org/10.1155/2014/547974.

Song, X. M., and Coauthors, 2014: Rapid urbanization and changes in spatiotemporal characteristics of precipitation in Beijing metropolitan area. *J. Geophys. Res.*, **119**, 11 250–11 271, https://doi.org/10.1002/2014JD022084.

Soriano, L. R., and F. de Pablo, 2002: Effect of small urban areas in central Spain on the enhancement of cloud-to-ground lightning activity. *Atmos. Environ.*, **36**, 2809–2816, https://doi.org/10.1016/S1352-2310(02)00204-2.

Soulhac, L., P. Salizzoni, F.-X. Cierco, and R. Perkins, 2011: The model SIRANE for atmospheric urban pollutant dispersion; part I, presentation of the model. *Atmos. Environ.*, **45**, 7379–7395, https://doi.org/10.1016/j.atmosenv.2011.07.008.

Spanton, A. M., and M. L. Williams, 1988: A comparison of the structure of the atmospheric boundary layers in central London and a rural/suburban site using acoustic sounding. *Atmos. Environ.*, **22**, 211–223, https://doi.org/10.1016/0004-6981(88)90029-7.

Stewart, I. D., 2011a: Redefining the urban heat island, PhD dissertation, University of British Columbia.

Stewart, I. D., 2011b: A systematic review and scientific critique of methodology in modern urban heat island literature. *International Journal of Climatology*, **31**, 200–217, https://doi.org/10.1002/joc.2141.

Stewart, I. D., and T. R. Oke, 2012: Local climate zones for urban temperature studies. *Bull. Amer. Meteor. Soc.*, **93**, 1879–1900, https://doi.org/10.1175/BAMS-D-11-00019.1.

Stewart, I. D., T. R. Oke, and E. S. Krayenhoff, 2014: Evaluation of the 'local climate zone' scheme using temperature observations and model simulations. *International Journal of Climatology*, **34**, 1062–1080, https://doi.org/10.1002/joc.3746.

Stoll, M. J., and Brazel, A. J., 1992: Surface-air temperature relationships in the urban environment of Phoenix, Arizona. *Physical Geography*, **13**(2), 160–179, https://doi.org/10.1080/02723646.1992.10642451.

Stone, B., J. J. Hess, and H. Frumkin, 2010: Urban form and extreme heat events: Are sprawling cities more vulnerable to climate change than compact cities. *Environmental Health Perspectives*, **118**, 1425–1428, https://doi.org/10.1289/ehp.0901879.

Su, Y.-F., G. M. Foody, and K.-S. Cheng, 2012: Spatial non-stationarity in the relationships between land cover and surface temperature in an urban heat island and its impacts on thermally sensitive populations. *Landscape and Urban Planning*, **107**, 172–180, https://doi.org/10.1016/j.landurbplan.2012.05.016.

Sun, Y., X. B. Zhang, G. Y. Ren, F. W. Zwiers, and T. Hu, 2016a: Contribution of urbanization to warming in China. *Nature Climate Change*, **6**, 706–709, https://doi.org/10.1038/nclimate2956.

Sun, T., C. S. B. Grimmond, and G.-H. Ni, 2016b: How do green roofs mitigate urban thermal stress under heat waves. *J. Geophys. Res.*, **121**, 5320–5335, https://doi.org/10.1002/2016JD024873.

Sun, Y., T. Hu, X. B. Zhang, C. Li, C. H. Lu, G. Y. Ren, and Z. H. Jiang, 2019: Contribution of global warming and urbanization to changes in temperature extremes in Eastern China. *Geophys. Res. Lett.*, **46**, 11 426–11 434, https://doi.org/10.1029/2019GL084281.

Sun, Y., N. Zhang, S. G. Miao, F. H. Kong, Y. Z. Zhang, and N. N. Li, 2021: Urban morphological parameters of the main cities in China and their application in the WRF model. *Journal of Advances in Modeling Earth Systems*, **13**, e2020MS002382, https://doi.org/10.1029/2020MS002382.

Sun, Y. M., and G. Augenbroe, 2014: Urban heat island effect on energy application studies of office buildings. *Energy and Buildings*, **77**, 171–179, https://doi.org/10.1016/j.enbuild.2014.03.055.

Svoma, B. M., and R. C. Balling Jr., 2009: An anthropogenic signal in Phoenix, Arizona winter precipitation. *Theor. Appl. Climatol.*, **98**, 315–321, https://doi.org/10.1007/s00704-009-0121-1.

Taha, H., 1999: Modifying a mesoscale meteorological model to better incorporate urban heat storage: A bulk-parameterization approach. *J. Appl. Meteorol. Climatol.*, **38**, 466–473, https://doi.org/10.1175/1520-0450(1999)038<0466:MAMMMT>2.0.CO;2.

Taleb, D., and B. Abu-Hijleh, 2013: Urban heat islands: Potential effect of organic and structured urban configurations on temperature variations in Dubai, UAE. *Renewable Energy*, **50**, 747–762, https://doi.org/10.1016/j.renene.2012.07.030.

Taleghani, M., L. Kleerekoper, M. Tenpierik, and A. Van Den Dobbelsteen, 2015: Outdoor thermal comfort within five different urban forms in the Netherlands. *Building and Environment*, **83**, 65–78, https://doi.org/10.1016/j.buildenv.2014.03.014.

Tao, W., and Coauthors, 2015: Effects of urban land expansion on the regional meteorology and air quality of eastern China. *Atmospheric Chemistry and Physics*, **15**, 8597–8614, https://doi.org/10.5194/acp-15-8597-2015.

Tapper, N. J., 1990: Urban influences on boundary layer temperature and humidity: Results from Christchurch, New Zealand. *Atmospheric Environment. Part B. Urban Atmosphere*, **24**, 19–27, https://doi.org/10.1016/0957-1272(90)90005-F.

Tewari, M., H. Kusaka, F. Chen, W. J. Coirier, S. Kim, A. A. Wyszogrodzki, and T. T. Warner, 2010: Impact of coupling a microscale computational fluid dynamics model with a mesoscale model on urban scale contaminant transport and dispersion. *Atmospheric Research*, **96**, 656–664, https://doi.org/10.1016/j.atmosres.2010.01.006.

Theeuwes, N. E., J. F. Barlow, A. J. Teuling, C. S. B. Grimmond, and S. Kotthaus, 2019: Persistent cloud cover over mega-cities linked to surface heat release. *npj Climate and Atmospheric Science*, **2**, 15, https://doi.org/10.1038/s41612-019-0072-x.

Thielen, J., W. Wobrock, A. Gadian, P. G. Mestayer, and J.-D. Creutin, 2000: The possible influence of urban surfaces on rainfall development: A sensitivity study in 2D in the meso-γ-scale. *Atmospheric Research*, **54**, 15–39, https://doi.org/10.1016/S0169-8095(00)00041-7.

Torres-Valcárcel, Á. R., J. Harbor, A. L. Torres-Valcárcel, and C. J. González-Avilés, 2015: Historical differences in temperature between urban and non-urban areas in Puerto Rico. *International Journal of Climatology*, **35**, 1648–1661, https://doi.org/10.1002/joc.4083.

Trusilova, K., B. Früh, S. Brienen, A. Walter, V. Masson, G. Pigeon, and P. Becker, 2013: Implementation of an urban parameterization scheme into the regional climate model COSMO-CLM. *J. Appl. Meteorol. Climatol.*, **52**, 2296–2311, https://doi.org/10.1175/JAMC-D-12-0209.1.

Tsilini, V., S. Papantoniou, D.-D. Kolokotsa, and E.-A. Maria, 2015: Urban gardens as a solution to energy poverty and urban heat island. *Sustainable Cities and Society*, **14**, 323–333, https://doi.org/10.1016/j.scs.2014.08.006.

Turner, D. B., 1964: A diffusion model for an urban area. *J. Appl. Meteorol. Climatol.*, **3**, 83–91, https://doi.org/10.1175/1520-0450(1964)003<0083:ADMFAU>2.0.CO;2.

Ueberham, M., and U. Schlink, 2018: Wearable sensors for multifactorial personal exposure measurements–A ranking study. *Environment International*, **121**, 130–138, https://doi.org/10.1016/j.envint.2018.08.057.

Ulpiani, G., 2021: On the linkage between urban heat island and urban pollution island: Three-decade literature review towards a conceptual framework. *Science of the Total Environment*, **751**, 141727, https://doi.org/10.1016/j.scitotenv.2020.141727.

UN DESA, 2018: *World Urbanization Prospects*: *The 2018 Revision*. United Nations Department of Economic and Social Affairs, Population Division.

Unger, J., Z. Sümeghy, and J. Zoboki, 2001: Temperature cross-section features in an urban area. *Atmospheric Research*, **58**, 117–127, https://doi.org/10.1016/S0169-8095(01)00087-4.

Uno, I., H. Ueda, and S. Wakamatsu, 1989: Numerical modeling of the nocturnal urban boundary layer. *Bound.-Layer Meteorol.*, **49**, 77–98, https://doi.org/10.1007/BF00116406.

Uno, I., S. Wakamatsu, H. Ueda, and A. Nakamura, 1988: An observational study of the structure of the nocturnal urban boundary layer. *Bound.-Layer Meteorol.*, **45**, 59–82, https://doi.org/10.1007/BF00120815.

Uno, I., S. Wakamatsu, H. Ueda, and A. Nakamura, 1992: Observed structure of the nocturnal urban boundary layer and its evolution into a convective mixed layer. *Atmospheric Environment. Part B. Urban Atmosphere*, **26**, 45–57, https://doi.org/10.1016/0957-1272(92)90036-R.

Van Den Heever, S. C., and W. R. Cotton, 2007: Urban aerosol impacts on downwind convective storms. *J. Appl. Meteorol. Climatol.*, **46**, 828–850, https://doi.org/10.1175/JAM2492.1.

Van Hove, L. W. A., C. M. J. Jacobs, B. G. Heusinkveld, J. A. Elbers, B. Van Driel, and A. A. M. Holtslag, 2015: Temporal and spatial variability of urban heat island and thermal comfort within the Rotterdam agglomeration. *Building and Environment*, **83**, 91–103, https://doi.org/10.1016/j.buildenv.2014.08.029.

Van Weverberg, K., K. De Ridder, and A. Van Rompaey, 2008: Modeling the contribution of the Brussels heat island to a long temperature time series. *J. Appl. Meteorol. Climatol.*, **47**, 976–990, https://doi.org/10.1175/2007JAMC1482.1.

Venter, Z. S., T. Chakraborty, and X. Lee, 2021: Crowdsourced air temperatures contrast satellite measures of the urban heat island and its mechanisms. *Science Advances*, **7**, eabb9569, https://doi.org/10.1126/sciadv.abb9569.

Voelkel, J., D. Hellman, R. Sakuma, and V. Shandas, 2018: Assessing vulnerability to urban heat: A study of disproportionate heat exposure and access to refuge by socio-demographic status in Portland, Oregon. *International Journal of Environmental Research and Public Health*, **15**, 640, https://doi.org/10.3390/ijerph15040640.

Vohra, K., and Coauthors, 2021: Long-term trends in air quality in major cities in the UK and India: A view from space. *Atmospheric Chemistry and Physics*, **21**(8), 6275–6296, https://doi.org/10.5194/acp-21-6275-2021.

Voogt, J., 2007: How researchers measure urban heat islands. Preprints, United States Environmental Protection Agency (EPA), State and Local Climate and Energy Program, Heat Island Effect, Urban Heat Island Webcasts and Conference Calls. [Available online at https://swap.stanford.edu/20120109061918/http://www.epa.gov/heatisland/resources/pdf/EPA_How_to_measure_a_UHI.pdf.]

Voogt, J. A., and T. R. Oke, 1998: Effects of urban surface geometry on remotely-sensed surface temperature. *Int. J. Remote Sens.*, **19**, 895–920, https://doi.org/10.1080/014311698215784.

Wang, F., and Q. S. Ge, 2012: Estimation of urbanization bias in observed surface temperature change in China from 1980 to 2009 using satellite land-use data. *Chinese Science Bulletin*, **57**, 1708–1715, https://doi.org/10.1007/s11434-012-4999-0.

Wang, F., Q. S. Ge, S. W. Wang, Q. X. Li, and P. D. Jones, 2015a: A new estimation of urbanization's contribution to the warming trend in China. *J. Climate*, **28**, 8923–8938, https://doi.org/10.1175/JCLI-D-14-00427.1.

Wang, J., J. M. Feng, Z. W. Yan, Y. H. Hu, and G. S. Jia, 2012: Nested high-resolution modeling of the impact of urbanization on regional climate in three vast urban agglomerations in China. *J. Geophys. Res.*, **117**, D21103, https://doi.org/10.1029/2012JD018226.

Wang, J., Z. W. Yan, Z. Li, W. D. Liu, and Y. X. Wang, 2013a: Impact of urbanization on changes in temperature extremes in Beijing during 1978–2008. *Chinese Science Bulletin*, **58**, 4679–4686, https://doi.org/10.1007/s11434-013-5976-y.

Wang, J., J. M. Feng, and Z. W. Yan, 2015b: Potential sensitivity of warm season precipitation to urbanization extents: Modeling study in Beijing-Tianjin-Hebei urban agglomeration in China. *J. Geophys. Res.*, **120**, 9408–9425, https://doi.org/10.1002/2015JD023572.

Wang, J., B. Huang, D. J. Fu, P. M. Atkinson, and X. Z. Zhang, 2016: Response of urban heat island to future urban expansion over the Beijing–Tianjin–Hebei metropolitan area. *Applied Geography*, **70**, 26–36, https://doi.org/10.1016/j.apgeog.2016.02.010.

Wang, J., Z. W. Yan, X.-W. Quan, and J. M. Feng, 2017a: Urban warming in the 2013 summer heat wave in eastern China. *Climate Dyn.*, **48**, 3015–3033, https://doi.org/10.1007/s00382-016-3248-7.

Wang, J., J. M. Feng, and Z. W. Yan, 2018: Impact of extensive urbanization on summertime rainfall in the Beijing region and the role of local precipitation recycling. *J. Geophys. Res.*, **123**, 3323–3340, https://doi.org/10.1002/2017JD027725.

Wang, J. A., L. R. Hutyra, D. Li, and M. A. Friedl, 2017b: Gradients of atmospheric temperature and humidity controlled by local urban land-use intensity in Boston. *J. Appl. Meteorol. Climatol.*, **56**, 817–831, https://doi.org/10.1175/JAMC-D-16-0325.1.

Wang, L., and D. Li, 2019: Modulation of the urban boundary-layer heat budget by a heatwave. *Quart. J. Roy. Meteor. Soc.*, **145**, 1814–1831, https://doi.org/10.1002/qj.3526.

Wang, L., and D. Li, 2021: Urban heat islands during heat waves: A comparative study between Boston and Phoenix. *J. Appl. Meteorol. Climatol.*, **60**, 621–641, https://doi.org/10.1175/JAMC-D-20-0132.1.

Wang, P., and Coauthors, 2021a: Urbanization contribution to human perceived temperature changes in major urban agglomerations of China. *Urban Climate*, **38**, 100910, https://doi.org/10.1016/j.uclim.2021.100910.

Wang, W.-C., Z. M. Zeng, and T. R. Karl, 1990: Urban heat islands in China. *Geophys. Res. Lett.*, **17**, 2377–2380, https://doi.org/10.1029/GL017i013p02377.

Wang, X. M., Z. Y. Wu, and G. X. Liang, 2009: WRF/CHEM modeling of impacts of weather conditions modified by urban expansion on secondary organic aerosol formation over Pearl River Delta. *Particuology*, **7**, 384–391, https://doi.org/10.1016/j.partic.2009.04.007.

Wang, X. X., and Y. G. Li, 2016: Predicting urban heat island circulation using CFD. *Building and Environment*, **99**, 82–97, https://doi.org/10.1016/j.buildenv.2016.01.020.

Wang, Y. P., U. Berardi, and H. Akbari, 2015c: The urban heat island effect in the city of Toronto. *Procedia Engineering*, **118**, 137–144, https://doi.org/10.1016/j.proeng.2015.08.412.

Wang, Y. W., J. J. Wu, Q. Du, and Y. H. Gao, 2013c: Numerical study of the Chongqing high-density buildings environment by the WRF with the different urban canopy schemes. *Acta Meteorologica Sinica*, **71**, 1130–1145. (in Chinese with English abstract)

Wang, Z.-H., E. Bou-Zeid, and J. A. Smith, 2013b: A coupled energy transport and hydrological model for urban canopies evaluated using a wireless sensor network. *Quart. J. Roy. Meteor. Soc.*, **139**, 1643–1657, https://doi.org/10.1002/qj.2032.

Wang, Z. X., J. Y. Song, P. W. Chan, and Y. G. Li, 2021b: The urban moisture island phenomenon and its mechanisms in a high-rise high-density city. *International Journal of Climatology*, **41**, E150–E170, https://doi.org/10.1002/joc.6672.

Ward, K., S. Lauf, B. Kleinschmit, and W. Endlicher, 2016: Heat waves and urban heat islands in Europe: A review of relevant drivers. *Science of the Total Environment*, **569–570**, 527–539, https://doi.org/10.1016/j.scitotenv.2016.06.119.

Weng, Q. H., and P. Fu, 2014: Modeling diurnal land temperature cycles over Los Angeles using downscaled GOES imagery. *ISPRS Journal of Photogrammetry and Remote Sensing*, **97**, 78–88, https://doi.org/10.1016/j.isprsjprs.2014.08.009.

Wienert, U., and W. Kuttler, 2005: The dependence of the urban heat island intensity on latitude A statistical approach. *Meteorologische Zeitschrift*, **14**, 677–686, https://doi.org/10.1127/0941-2948/2005/0069.

Wilby, R. L., 2003: Past and projected trends in London's urban heat island. *Weather*, **58**, 251–260, https://doi.org/10.1256/wea.183.02.

Williams, A. P., and Coauthors, 2015: Urbanization causes increased cloud base height and decreased fog in coastal Southern California. *Geophys. Res. Lett.*, **42**, 1527–1536, https://doi.org/10.1002/2015GL063266.

Winkler, M., B. Steuri, S. Stalder, and F. Antretter, 2020: Evaluating the practicability of the new urban climate model PALM-4U using a living-lab approach. *E3S Web of Conferences*, **172**, 11010, https://doi.org/10.1051/e3sconf/202017211010.

WMO, 2008: *Guide to Meteorological Instruments and Methods of Observation*. World Meteorological Organization.

Wouters, H., M. Demuzere, U. Blahak, K. Fortuniak, B. Maiheu, J. Camps, D. Tielemans, and N. P. M. Van Lipzig, 2016: The efficient urban canopy dependency parametrization (SURY) v1.0 for atmospheric modelling: Description and application with the COSMO-CLM model for a Belgian summer. *Geoscientific Model Development*, **9**, 3027–3054, https://doi.org/10.5194/gmd-9-3027-2016.

Wu, K., and X. Q. Yang, 2013: Urbanization and heterogeneous surface warming in eastern China. *Chinese Science Bulletin*, **58**, 1363–1373, https://doi.org/10.1007/s11434-012-5627-8.

Wu, M. W., Y. L. Luo, F. Chen, and W. K. Wong, 2019: Observed link of extreme hourly precipitation changes to urbanization over coastal South China. *J. Appl. Meteorol. Climatol.*, **58**, 1799–1819, https://doi.org/10.1175/JAMC-D-18-0284.1.

Wu, S. J., P. Wang, X. L. Tong, H. Tian, Y. Q. Zhao, and M. Luo, 2021: Urbanization-driven increases in summertime compound heat extremes across China. *Science of the Total Environment*, **799**, 149166, https://doi.org/10.1016/j.scitotenv.2021.149166.

Wu, X. J., L. C. Wang, R. Yao, M. Luo, S. Q. Wang, and L. Z. Wang, 2020: Quantitatively evaluating the effect of urbanization on heat waves in China. *Science of the Total Environment*, **731**, 138857, https://doi.org/10.1016/j.scitotenv.2020.138857.

Wu, Z. F., and Y. Ren, 2019: A bibliometric review of past trends and future prospects in urban heat island research from 1990 to 2017. *Environmental Reviews*, **27**, 241–251, https://doi.org/10.1139/er-2018-0029.

Wyszogrodzki, A. A., and P. K. Smolarkiewicz, 2009: Building resolving large-eddy simulations (LES) with EULAG. Preprints, *Proc. Academy Colloquium on Immersed Boundary Methods: Current Status and Future Research Directions*, 15–17.

Xu, X. Y., F. Chen, S. H. Shen, S. G. Miao, M. Barlage, W. L. Guo, and A. Mahalov, 2018: Using WRF-urban to assess summertime air conditioning electric loads and their impacts on urban weather in Beijing. *J. Geophys. Res.*, **123**, 2475–2490, https://doi.org/10.1002/2017JD028168.

Yaghoobian, N., and J. Kleissl, 2012: An indoor–outdoor building energy simulator to study urban modification effects on building energy use–Model description and validation. *Energy and Buildings*, **54**, 407–417, https://doi.org/10.1016/j.enbuild.2012.07.019.

Yan, Z.-W., J. Wang, J.-J. Xia, and J.-M. Feng, 2016: Review of recent studies of the climatic effects of urbanization in China. *Advances in Climate Change Research*, **7**, 154–168, https://doi.org/10.1016/j.accre.2016.09.003.

Yang, B., Y. C. Zhang, and Y. Qian, 2012: Simulation of urban climate with high-resolution WRF model: A case study in Nanjing, China. *Asia-Pacific Journal of Atmospheric Sciences*, **48**, 227–241, https://doi.org/10.1007/s13143-012-0023-5.

Yang, B., and Coauthors, 2019a: Modeling the impacts of urbanization on summer thermal comfort: The role of urban land use and anthropogenic heat. *J. Geophys. Res.*, **124**, 6681–6697, https://doi.org/10.1029/2018JD029829.

Yang, J. C., Z. H. Wang, F. Chen, S. G. Miao, M. Tewari, J. A. Voogt, and S. Myint, 2015: Enhancing hydrologic modelling in the coupled weather research and forecasting–urban modelling system. *Bound.-Layer Meteorol.*, **155**, 87–109, https://doi.org/10.1007/s10546-014-9991-6.

Yang, L., F. Q. Tian, J. A. Smith, and H. P. Hu, 2014: Urban signatures in the spatial clustering of summer heavy rainfall events over the Beijing metropolitan region. *J. Geophys. Res.*, **119**, 1203–1217, https://doi.org/10.1002/2013JD020762.

Yang, P., G. Y. Ren, W. Hou, and W. D. Liu, 2013: Spatial and diurnal characteristics of summer rainfall over Beijing Municipality based on a high-density AWS dataset. *International Journal of Climatology*, **33**, 2769–2780, https://doi.org/10.1002/joc.3622.

Yang, Q. Q., X. Huang, and Q. H. Tang, 2019b: The footprint of urban heat island effect in 302 Chinese cities: Temporal trends and associated factors. *Science of the Total Environment*, **655**, 652–662, https://doi.org/10.1016/j.scitotenv.2018.11.171.

Yang, X. C., Y. L. Hou, and B. D. Chen, 2011: Observed surface warming induced by urbanization in east China. *J. Geophys. Res.*, **116**, D14113, https://doi.org/10.1029/2010JD015452.

Yang, X. C., L. R. Leung, N. Z. Zhao, C. Zhao, Y. Qian, K. J.

Hu, X. P. Liu, and B. D. Chen, 2017: Contribution of urbanization to the increase of extreme heat events in an urban agglomeration in east China. *Geophys. Res. Lett.*, **44**, 6940–6950, https://doi.org/10.1002/2017GL074084.

Yang, Z., F. Dominguez, H. Gupta, X. B. Zeng, and L. Norman, 2016: Urban effects on regional climate: A case study in the Phoenix and Tucson "Sun Corridor". *Earth Interactions*, **20**, 1–25, https://doi.org/10.1175/EI-D-15-0027.1.

Yao, R., L. C. Wang, X. Huang, W. Gong, and X. G. Xia, 2019: Greening in rural areas increases the surface urban heat island intensity. *Geophys. Res. Lett.*, **46**, 2204–2212, https://doi.org/10.1029/2018GL081816.

Yao, R., L. C. Wang, S. Q. Wang, L. Z. Wang, J. Wei, J. L. Li, and D. Q. Yu, 2020: A detailed comparison of MYD11 and MYD21 land surface temperature products in mainland China. *International Journal of Digital Earth*, **13**, 1391–1407, https://doi.org/10.1080/17538947.2019.1711211.

Yin, C. H., M. Yuan, Y. P. Lu, Y. P. Huang, and Y. F. Liu, 2018: Effects of urban form on the urban heat island effect based on spatial regression model. *Science of the Total Environment*, **634**, 696–704, https://doi.org/10.1016/j.scitotenv.2018.03.350.

Yokoyama, H., R. Ooka, and H. Kikumoto, 2018: Study of mobile measurements for detailed temperature distribution in a high-density urban area in Tokyo. *Urban Climate*, **24**, 517–528, https://doi.org/10.1016/j.uclim.2017.06.006.

Yoshikado, H., 1992: Numerical study of the daytime urban effect and its interaction with the sea breeze. *J. Appl. Meteorol. Climatol.*, **31**, 1146–1164, https://doi.org/10.1175/1520-0450(1992)031<1146:NSOTDU>2.0.CO;2.

Yu, M., and Y. M. Liu, 2015: The possible impact of urbanization on a heavy rainfall event in Beijing. *J. Geophys. Res.*, **120**, 8132–8143, https://doi.org/10.1002/2015JD023336.

Yu, M., G. R. Carmichael, T. Zhu, and Y. F. Cheng, 2012: Sensitivity of predicted pollutant levels to urbanization in China. *Atmos. Environ.*, **60**, 544–554, https://doi.org/10.1016/j.atmosenv.2012.06.075.

Zarrella, A., E. Prataviera, P. Romano, L. Carnieletto, and J. Vivian, 2020: Analysis and application of a lumped-capacitance model for urban building energy modelling. *Sustainable Cities and Society*, **63**, 102450, https://doi.org/10.1016/j.scs.2020.102450.

Zhan, W. F., W. M. Ju, S. P. Hai, G. Ferguson, J. L. Quan, C. S. Tang, Z. Guo, and F. H. Kong, 2014: Satellite-derived subsurface urban heat island. *Environ. Sci. Technol.*, **48**, 12 134–12 140, https://doi.org/10.1021/es5021185.

Zhang, C. L., F. Chen, S. G. Miao, Q. C. Li, X. A. Xia, and C. Y. Xuan, 2009: Impacts of urban expansion and future green planting on summer precipitation in the Beijing metropolitan area. *J. Geophys. Res.*, **114**, D02116, https://doi.org/10.1029/2008JD010328.

Zhang, D.-L., Y.-X. Shou, and R. R. Dickerson, 2009c: Upstream urbanization exacerbates urban heat island effects. *Geophys. Res. Lett.*, **36**, L24401, https://doi.org/10.1029/2009GL041082.

Zhang, G. J., M. Cai, and A. X. Hu, 2013: Energy consumption and the unexplained winter warming over northern Asia and North America. *Nature Climate Change*, **3**, 466–470, https:/ /doi.org/10.1038/nclimate1803.

Zhang, N., Z. Q. Gao, X. M. Wang, and Y. Chen, 2010: Modeling the impact of urbanization on the local and regional climate in Yangtze River Delta, China. *Theor. Appl. Climatol.*, **102**, 331–342, https://doi.org/10.1007/s00704-010-0263-1.

Zhang, N., X. Y. Wang, and Z. Peng, 2014a: Large-eddy simulation of mesoscale circulations forced by inhomogeneous urban heat island. *Bound.-Layer Meteorol.*, **151**, 179–194, https://doi.org/10.1007/s10546-013-9879-x.

Zhang, P., L. Bounoua, M. L. Imhoff, R. E. Wolfe, and K. Thome, 2014b: Comparison of MODIS land surface temperature and air temperature over the continental USA meteorological stations. *Canadian Journal of Remote Sensing*, **40**, 110–122, https://doi.org/10.1080/07038992.2014.935934.

Zhang, Q. M., M. Zhang, W. Q. Zhou, W. B. Xu, and J. Zhang, 2019a: The influence of different urban and rural selection methods on the spatial variation of urban heat island intensity. Preprints, IGARSS 2019–2019 IEEE Int. Geoscience and Remote Sensing Symposium, Yokohama, Japan, IEEE, 4403–4406, https://doi.org/10.1109/IGARSS.2019.8898794.

Zhang, W., G. Villarini, G. A. Vecchi, and J. A. Smith, 2018: Urbanization exacerbated the rainfall and flooding caused by hurricane Harvey in Houston. *Nature*, **563**, 384–388, https://doi.org/10.1038/s41586-018-0676-z.

Zhang, Y., J. A. Smith, L. F. Luo, Z. F. Wang, and M. L. Baeck, 2014c: Urbanization and rainfall variability in the Beijing metropolitan region. *Journal of Hydrometeorology*, **15**, 2219–2235, https://doi.org/10.1175/JHM-D-13-0180.1.

Zhang, Y. J., D. Li, Z. K. Lin, J. A. Santanello Jr., and Z. Q. Gao, 2019b: Development and evaluation of a long-term data record of planetary boundary layer profiles from aircraft meteorological reports. *J. Geophys. Res.*, **124**, 2008–2030, https://doi.org/10.1029/2018JD029529.

Zhang, Y. J., K. Sun, Z. Q. Gao, Z. T. Pan, M. A. Shook, and D. Li, 2020a: Diurnal climatology of planetary boundary layer height over the contiguous United States derived from AMDAR and reanalysis data. *J. Geophys. Res.*, **125**, e2020JD032803, https://doi.org/10.1029/2020JD032803.

Zhang, Y. J., L. Wang, J. A. Santanello Jr., Z. T. Pan, Z. Q. Gao, and D. Li, 2020b: Aircraft observed diurnal variations of the planetary boundary layer under heat waves. *Atmospheric Research*, **235**, 104801, https://doi.org/10.1016/j.atmosres.2019.104801.

Zhao, L., X. Lee, R. B. Smith, and K. Oleson, 2014: Strong contributions of local background climate to urban heat islands. *Nature*, **511**, 216–219, https://doi.org/10.1038/nature13462.

Zhao, L., X. Lee, and N. M. Schultz, 2017: A wedge strategy for mitigation of urban warming in future climate scenarios. *Atmospheric Chemistry and Physics*, **17**, 9067–9080, https://doi.org/10.5194/acp-17-9067-2017.

Zhao, L., M. Oppenheimer, Q. Zhu, J. W. Baldwin, K. L. Ebi, E. Bou-Zeid, K. Y. Guan, and X. Liu, 2018: Interactions between urban heat islands and heat waves. *Environmental Research Letters*, **13**, 034003, https://doi.org/10.1088/1748-9326/aa9f73.

Zheng, Z. F., and Coauthors, 2018: Relationship between fine-particle pollution and the urban heat island in Beijing, China: Observational evidence. *Bound.-Layer Meteorol.*, **169**, 93–113, https://doi.org/10.1007/s10546-018-0362-6.

Zheng, Z. H., L. Zhao, and K. W. Oleson, 2021: Large model structural uncertainty in global projections of urban heat waves. *Nature Communications*, **12**, 3736, https://doi.org/10.1038/s41467-021-24113-9.

Zhong, S., Y. Qian, C. Zhao, R. Leung, and X.-Q. Yang, 2015: A

case study of urbanization impact on summer precipitation in the Greater Beijing Metropolitan Area: Urban heat island versus aerosol effects. *J. Geophys. Res.*, **120**, 10 903–10 914, https://doi.org/10.1002/2015JD023753.

Zhong, S., and Coauthors, 2017: Urbanization-induced urban heat island and aerosol effects on climate extremes in the Yangtze River Delta region of China. *Atmospheric Chemistry and Physics*, **17**, 5439–5457, https://doi.org/10.5194/acp-17-5439-2017.

Zhong, S., and Coauthors, 2018: Urbanization effect on winter haze in the Yangtze River Delta region of China. *Geophys. Res. Lett.*, **45**, 6710–6718, https://doi.org/10.1029/2018GL077239.

Zhong, T., N. Zhang, and M. Y. Lv, 2021: A numerical study of the urban green roof and cool roof strategies' effects on boundary layer meteorology and ozone air quality in a megacity. *Atmos. Environ.*, **264**, 118702, https://doi.org/10.1016/j.atmosenv.2021.118702.

Zhou, B., D. Lauwaet, H. Hooyberghs, K. De Ridder, J. P. Kropp, and D. Rybski, 2016: Assessing seasonality in the surface urban heat island of London. *J. Appl. Meteorol. Climatol.*, **55**, 493–505, https://doi.org/10.1175/JAMC-D-15-0041.1.

Zhou, B., D. Rybski, and J. P. Kropp, 2017: The role of city size and urban form in the surface urban heat island. *Scientific Reports*, **7**, 4791, https://doi.org/10.1038/s41598-017-04242-2.

Zhou, D. C., S. Q. Zhao, L. X. Zhang, G. Sun, and Y. Q. Liu, 2015a: The footprint of urban heat island effect in China. *Scientific Reports*, **5**, 11160, https://doi.org/10.1038/srep11160.

Zhou, D. C., and Coauthors, 2019: Satellite remote sensing of surface urban heat islands: Progress, challenges, and perspectives. *Remote Sensing*, **11**, 48, https://doi.org/10.3390/rs11010048.

Zhou, L. M., R. E. Dickinson, Y. H. Tian, J. Y. Fang, Q. X. Li, R. K. Kaufmann, C. J. Tucker, and R. B. Myneni, 2004: Evidence for a significant urbanization effect on climate in China. *Proceedings of the National Academy of Sciences of the United States of America*, **101**, 9540–9544, https://doi.org/10.1073/pnas.0400357101.

Zhou, Y. Y., and Coauthors, 2015b: A global map of urban extent from nightlights. *Environmental Research Letters*, **10**, 054011, https://doi.org/10.1088/1748-9326/10/5/054011.

Zhu, X. L., G. H. Ni, Z. T. Cong, T. Sun, and D. Li, 2016: Impacts of surface heterogeneity on dry planetary boundary layers in an urban-rural setting. *J. Geophys. Res.*, **121**, 12 164–12 179, https://doi.org/10.1002/2016JD024982.

Zipper, S. C., J. Schatz, C. J. Kucharik, and S. P. Loheide II, 2017: Urban heat island-induced increases in evapotranspirative demand. *Geophys. Res. Lett.*, **44**, 873–881, https://doi.org/10.1002/2016GL072190.

Ziter, C. D., E. J. Pedersen, C. J. Kucharik, and M. G. Turner, 2019: Scale-dependent interactions between tree canopy cover and impervious surfaces reduce daytime urban heat during summer. *Proceedings of the National Academy of Sciences of the United States of America*, **116**, 7575–7580, https://doi.org/10.1073/pnas.1817561116.

Zonato, A., A. Martilli, S. Di Sabatino, D. Zardi, and L. Giovannini, 2020: Evaluating the performance of a novel WUDAPT averaging technique to define urban morphology with mesoscale models. *Urban Climate*, **31**, 100584, https://doi.org/10.1016/j.uclim.2020.100584.

Zonato, A., A. Martilli, E. Gutierrez, F. Chen, C. L. He, M. Barlage, D. Zardi, and L. Giovannini, 2021: Exploring the role of rooftop urban mitigation strategies in thermal comfort and energy consumption. *Earth and Space Science Open Archive*, **32**, https://doi.org/10.1002/essoar.10506605.
